# Beet red food colourant can be produced more sustainably with engineered *Yarrowia lipolytica*

**DOI:** 10.1038/s41564-023-01517-5

**Published:** 2023-11-29

**Authors:** Philip Tinggaard Thomsen, Samir Meramo, Lorenzo Ninivaggi, Eleonora Pasutto, Mahsa Babaei, Paulo Marcelo Avila-Neto, Marc Cernuda Pastor, Peyman Sabri, Daniela Rago, Tanmay Utsav Parekh, Sara Hunding, Laura Emilie Jul Christiansen, Sumesh Sukumara, Irina Borodina

**Affiliations:** The Novo Nordisk Foundation Center for Biosustainability, Technical University of Denmark, Lyngby, Denmark

**Keywords:** Metabolic engineering, Environmental economics

## Abstract

Synthetic food colourants are widely used in the food industry, but consumer concerns about safety and sustainability are driving a need for natural food-colour alternatives. Betanin, which is extracted from red beetroots, is a commonly used natural red food colour. However, the betanin content of beetroot is very low (~0.2% wet weight), which means that the extraction of betanin is incredibly wasteful in terms of land use, processing costs and vegetable waste. Here we developed a sustainability-driven biotechnological process for producing red beet betalains, namely, betanin and its isomer isobetanin, by engineering the oleaginous yeast *Yarrowia lipolytica*. Metabolic engineering and fermentation optimization enabled production of 1,271 ± 141 mg l^−1^ betanin and 55 ± 7 mg l^−1^ isobetanin in 51 h using glucose as carbon source in controlled fed-batch fermentations. According to a life cycle assessment, at industrial scale (550 t yr^−1^), our fermentation process would require significantly less land, energy and resources compared with the traditional extraction of betanin from beetroot crops. Finally, we apply techno-economic assessment to show that betanin production by fermentation could be economically feasible in the existing market conditions.

## Abstract (web summary)

Sustainable, gram-scale production of beet red food colourant with engineered *Yarrowia lipolytica* is described.

## Main

Colour is an essential feature of food and beverages. Food colour affects consumer perception of flavour, freshness and product quality. Natural and synthetic food dyes are therefore frequently added to processed foods to enhance or correct food colour to meet consumer expectations. Concerns raised by consumers and food authorities alike about synthetic food additives have heightened the demand for natural food colourants. The global market for natural food colours was estimated at US$1.5–1.75 billion in 2022 and is expected to grow^1^. Red colourants are commonly added to beverages, confectionaries, dairy products, meats, cereals and various other foods, and are among the most widely used pigments in the food industry^2^. Betanin is a red colourant that is extracted from red beetroots and marketed as the food additive ‘beetroot red’ (E162). Here beetroot red is typically formulated in water or as a spray dried maltodextrin powder with a betanin concentration of 0.4–1.2% (ref. ^3^). Beetroot red is increasingly being used to replace synthetic pigments, such as Allura Red AC (E129), which is banned in several European Union countries^4,5^. Although natural dyes can be extracted from cultivated plants, extraction is inherently wasteful and inefficient owing to the low content of pigments natively found in these plants^6,7^. For instance, red beets contain ~20–210 mg betanin per 100 g fresh weight, depending on the cultivar, with an average crop yield of 50–70 tons ha^−1^ (refs. ^6,8^). In addition, pigment extraction is challenging, with a typical product recovery of only 60% (ref. ^7^). As consumer demand drives the replacement of synthetic food colourants, the arable land required to satisfy the food industry’s demand for this natural red pigment is predicted only to increase^9^.

Betanin production has been engineered in traditional crops, for example, *Nicotiana benthamiana*, *Solanum lycopersicum* (tomato) and *Oryza sativa japonica* (rice), enabling red beet pigment production of up to 270 mg betanin per 100 g fresh weight—notably more than typically found in red beets^10–12^. However, these plant-based betanin production systems are similarly dependent on arable land, and yields depend on the climate and are subject to seasonal changes. Biosynthetic production of betanin from renewable feedstocks using microbial fermentation could provide a more sustainable and lower-cost alternative to extraction of the pigment from plants. Betanin production has been engineered in *Saccharomyces cerevisiae*, initially at 17 mg l^−1^ (ref. ^13^), then improved to 29 mg l^−1^ (ref. ^14^) and then to 31 mg l^−1^ (ref. ^15^). For microbially produced betanin to be a viable alternative to plant-extracted betanin, it must be cost-competitive, which requires efficient production.

Here we engineered betanin production in the oleaginous yeast *Yarrowia lipolytica*. *Y. lipolytica* is well suited to industrial fermentation as it is Crabtree negative, which means that it has limited overflow metabolism in the presence of excess sugar, making it relatively easy to ferment at large scale^16^. Furthermore, *Y. lipolytica* was previously engineered to produce a remarkably high titre of shikimate-derived resveratrol, indicating a capacity to generate a high flux through the aromatic amino acid biosynthesis pathway—which provides the primary building blocks of the red beet pigments^17^. We also applied life cycle assessment (LCA) and techno-economic assessment (TEA) to evaluate the sustainability performance and economic viability of our fermentation-based betanin production process and report our findings here.

## Results

### Engineering de novo betanin production in *Y. lipolytica*

**Fig. 1:**
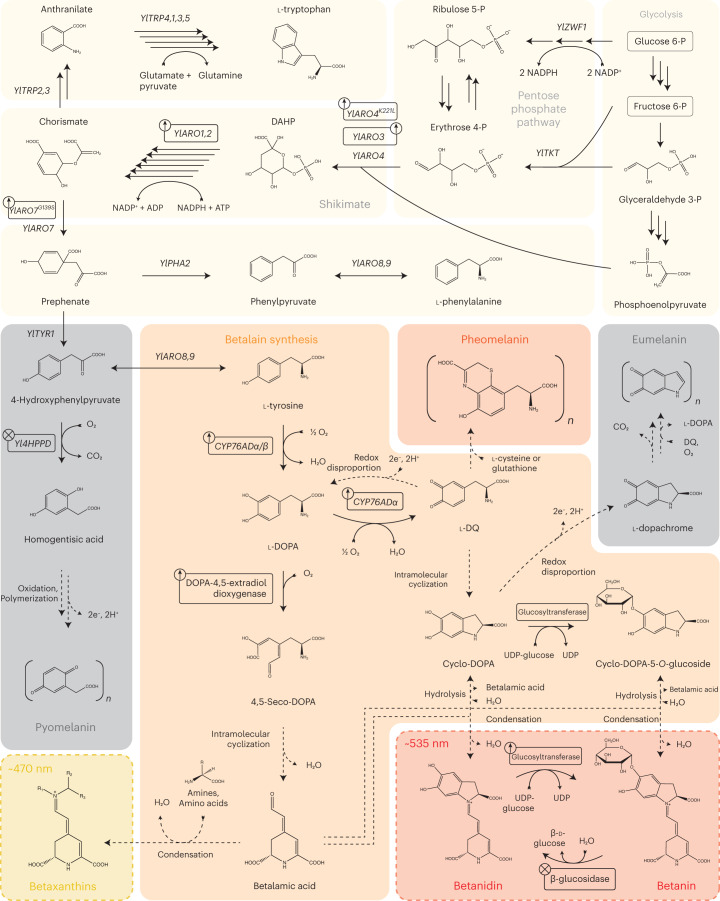
The heterologous betalain biosynthesis pathway in connection with native *Y. lipolytica* metabolism. The primary betalains are depicted in dashed boxes. Multiple arrows represent enzymatic reactions grouped for simplicity. Dashed arrows represent spontaneous reactions. Boxes with ‘up arrows’ indicate gene overexpressions, and boxes with ‘crosses’ indicate gene disruptions.

Betanin is derived from l-tyrosine via the shikimate pathway. Its biosynthesis begins with the hydroxylation of l-tyrosine into 3,4-dihydroxy-l-phenylalanine (l-DOPA) by a bifunctional cytochrome P450 (CYP) monooxygenase of the CYP76ADα family (herein tyrosine hydroxylase (TYH)) (Fig. 1). Hereafter, l-DOPA is oxidized in parallel either by TYH to form l-dopaquinone (DQ) or by a DOPA-4,5-extradiol dioxygenase (herein DOD) to form 4,5-seco-DOPA, which undergoes spontaneous cyclization to form betalamic acid. l-DQ is a highly reactive *ortho*-quinone, which can undergo intramolecular cyclization to form cyclo-DOPA^18^. Once formed, cyclo-DOPA spontaneously condenses with betalamic acid to form betanidin, the labile betanin precursor. Finally, a betanidin glycosyltransferase (GT) attaches a glycosyl group via uridine diphosphate (UDP)-glucose at the 5-hydroxyl position of betanidin, yielding betanin (betanidin-5-*O*-β-glucoside). Alternatively, cyclo-DOPA can be glycosylated before condensation with betalamic acid by a cyclo-DOPA-5-*O*-glycosyltransferase^19^.

**Fig. 2:**
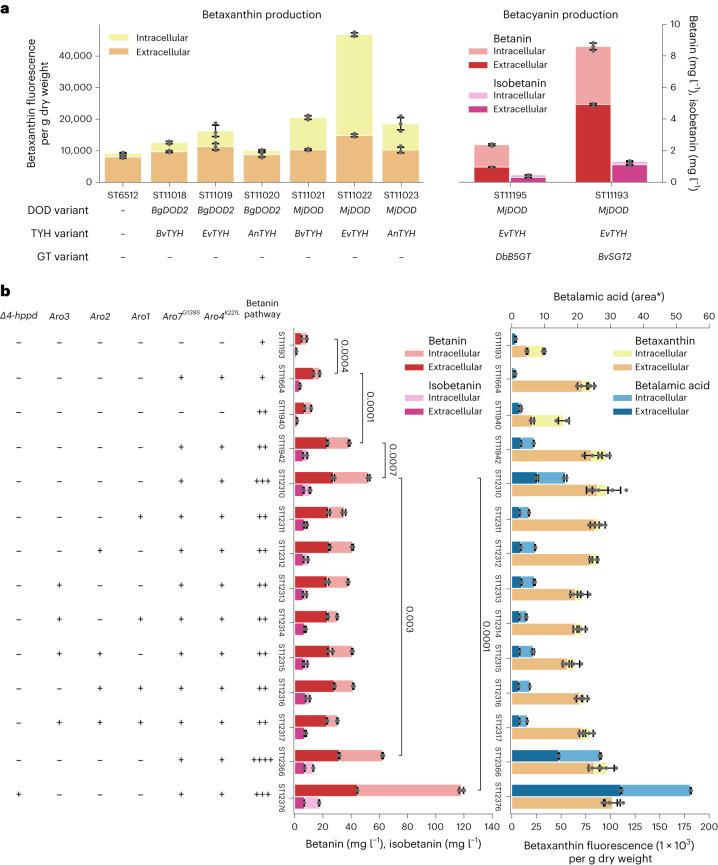
Metabolic engineering of *Y. lipolytica* for betanin production. **a**, Variant testing of betalain biosynthesis enzymes in *Y. lipolytica*. **b**, Metabolic engineering of *Y. lipolytica* for improved betanin production. Betaxanthin production was compared between strains by assessing the fluorescence of the supernatant and cell lysate at the betaxanthin-typical fluorescence profile (excitation, 463 nm; emission, 512 nm) and adjusted for cell dry weight. Betanin and isobetanin production was quantified via HPLC analysis. ‘−’ and ‘+’ symbols indicate the absence and presence, respectively, of the corresponding genetic modification or enzyme class. ‘area*’ indicates HPLC quantification by UV-vis spectra, without internal standards. Strains were inoculated from precultures into mineral media to approximately an OD_660_ of 0.1. Cultivations were carried out in biological triplicate (*n* = 3). Statistical analysis was performed on the total betanin production via Student’s *t*-test (one tailed; paired). The bars indicate mean production titre/fluorescence, and the error bars depict the corresponding standard deviations. Source data

**Extended Data Fig. 1:**
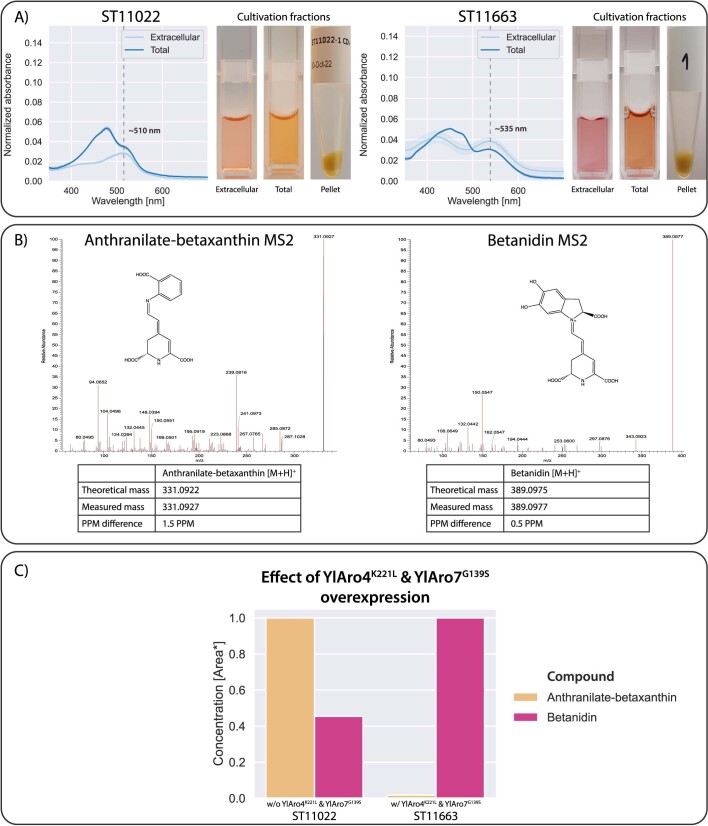
Effect of *YlARO4*^*K221L*^ & *YlARO7*^*G139S*^ overexpression on anthranilic acid-betaxanthin formation. **a)** UvVis spectra of extracellular and total cell fractions from ST11022 (without *YlARO4*^*K221L*^ & *YlARO7*^*G139S*^) and ST11663 (with *YlARO4*^*K221L*^ & *YlARO7*^*G139S*^). The solid lines represent mean absorbance and the shaded areas represent the standard deviations. **b)** MS2 mass spectra of anthranilate-betaxanthin (primarily detected in ST11022) and betanidin. **c)** Comparison of the peak areas of anthranilate-betaxanthin and betanidin in ST11022 and ST11663. Overexpression of *YlARO4*^*K221L*^ & *YlARO7*^*G139S*^ mostly eliminates anthranilate-betaxanthin and greatly boosts betanidin production. Cultivations were carried out in biological triplicate (n = 3), but only a single replicate was chosen for LC-MS analysis as exact quantification was not the goal, since an authentic analytical standard is not available for betanidin. Source data

To establish betanin biosynthesis in *Y. lipolytica*, we applied results from our previous work in which we screened TYH and DOD variants in *S. cerevisiae* in a high-throughput, combinatorial manner to identify catalytically favourable enzyme variants and combinations^15^. From that screen, we selected *BgDOD2* from *Bougainvillea glabra*, *BvTYH* from *Beta vulgaris*, *EvTYH* from *Ercilla volubilis* and *AnTYH* from *Abronia nealleyi* to test in *Y. lipolytica*. In addition, we included the engineered version of *B. vulgaris* CYP76AD1 (herein referred to as *BvTYH*), containing the W13L mutation found to increase catalytic activity, and *MjDOD* from *Mirabilis jalapa*^20,21^. The TYH- and DOD-encoding codon-optimized genes were expressed combinatorically in *Y. lipolytica*, and betaxanthin production was evaluated by fluorescence (Fig. 2a, left). We found that the combination of *MjDOD* and *EvTYH* (ST11022) outperformed other combinations by more than twofold. In addition, we observed that the resulting cultivation broth was orange (peak absorption wavelength, *λ*_max_ = 510 nm), and liquid chromatography–mass spectrometry (LC–MS) analysis of the supernatant revealed an accumulation of an anthranilate–betaxanthin conjugate (Extended Data Fig. 1a).

Having confirmed via LC–MS analysis that the *Y. lipolytica* strain expressing *MjDOD* and *EvTYH* (ST11022) produced betanidin, an unstable conjugate of betalamic acid and cyclo-DOPA (Extended Data Fig. 1b,c), we searched for a glycosyltransferase capable of efficiently converting betanidin into the far more stable pigment betanin^22^. We tested the *B. vulgaris* scopoletin glycosyltransferase (*BvSGT2*) that we have previously reported as highly active in *S. cerevisiae*^15^ against a betanidin glycosyltransferase from *Dorotheanthus bellidiformis* (*DbB5GT*) (Fig. 2a, right)^23^. Completing the betanin biosynthesis pathway with *BvSGT2* resulted in ~9.9 mg l^−1^ betacyanins (8.6 mg l^−1^ betanin, 1.3 mg l^−1^ isobetanin), nearly threefold more than with *DbB5GT*. Unexpectedly, we observed that betanin was present both in the cells (~40%) and in the supernatant (~60%), while isobetanin was mainly found in the supernatant. As isobetanin exhibits identical chromatic properties to betanin, and can account for up to 45% of the betacyanins in beetroot red (E162), we will, for the purpose of this paper, consider them both as desired products, but present them separately for the sake of accuracy^3^.

### Engineering increased betanin production in *Y. lipolytica*

To improve betanin production in *Y. lipolytica*, we introduced feedback-insensitive alleles of 3-deoxy-d-arabinoheptulosonate 7-phosphate (DAHP) synthase and chorismate mutase, namely, *YlARO4*^*K221L*^ and *YlARO7*^*G141S*^, which in combination have been shown to improve the production of l-tyrosine-derived aromatics in *Y. lipolytica*^17,24^. Expressing *YlARO4*^*K221L*^ and *YlARO7*^*G141*^ in a *Y. lipolytica* strain containing only *EvTYH* and *MjDOD* resulted in a shift in the cultivation supernatant’s *λ*_max_ from 510 nm to 540 nm, indicative of betanidin rather than anthranilate–betaxanthin accumulation (Extended Data Fig. 1). This shift was confirmed by LC–MS analysis. When *YlARO4*^*K221L*^ and *YlARO7*^*G141*^ were expressed in a betanin-producing strain containing the glucosyltransferase *BvSGT2* (ST11664), betacyanin production was improved 2.1-fold to ~21.7 mg l^−1^ (17.7 mg l^−1^ betanin, 4.0 mg l^−1^ isobetanin).

**Extended Data Fig. 2:**
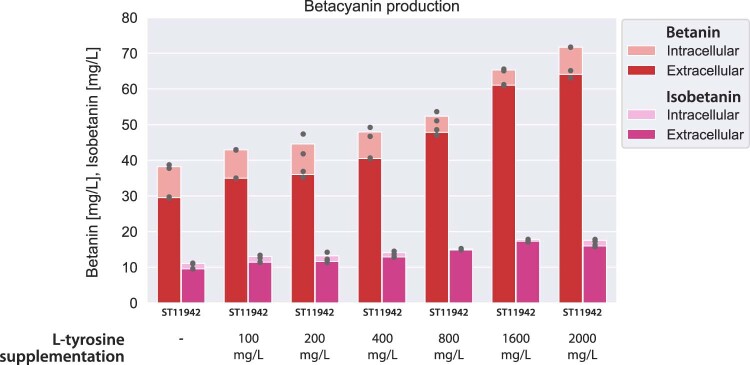
L-tyrosine supplementation improves betanin production. L-tyrosine supplemented at various degrees to mineral media via a highly concentrated (50 g/L) L-tyrosine stock, to prevent media dilution. The pH was back-adjusted to its original value by adding equimolar 1M NaOH. Cultivations were carried out in biological duplicate (n = 2). The bars indicate mean production titres. Source data

**Extended Data Fig. 3:**
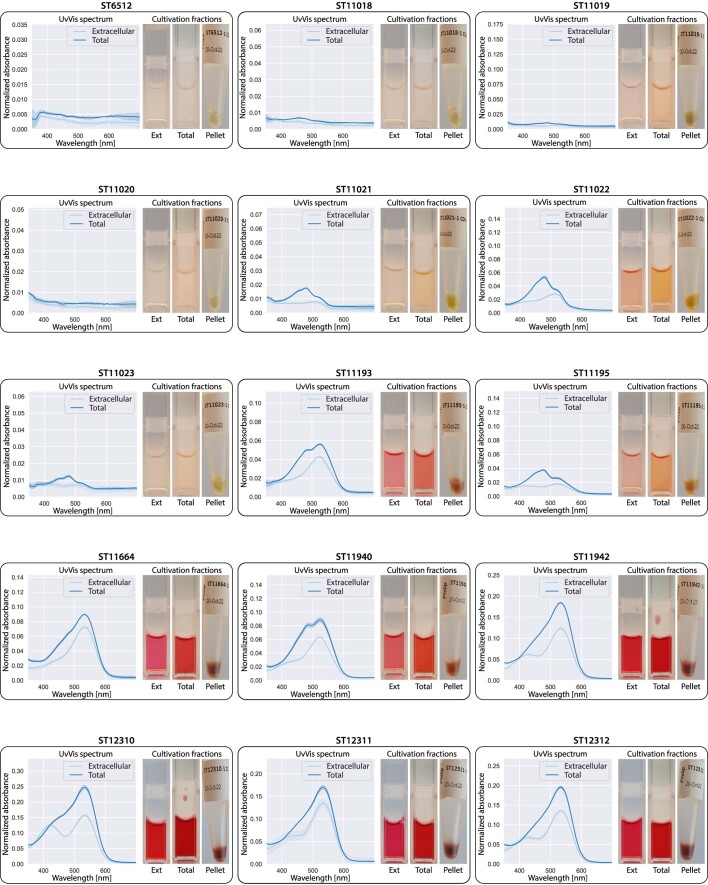
UV-vis spectra of the extracellular and total fractions from the betalain-producing *Y. lipolytica* strains. UV-vis spectra of 20X-diluted extracellular and total samples displayed on the left, with the corresponding non-diluted fractions displayed on the right. The pellet was derived from the 0.5 mL cell culture spun down to acquire the extracellular sample (non-disrupted). Cultivations were carried out in biological triplicate (n = 3), with the solid lines representing averages and shaded areas representing the standard deviations. Source data

**Extended Data Fig. 4:**
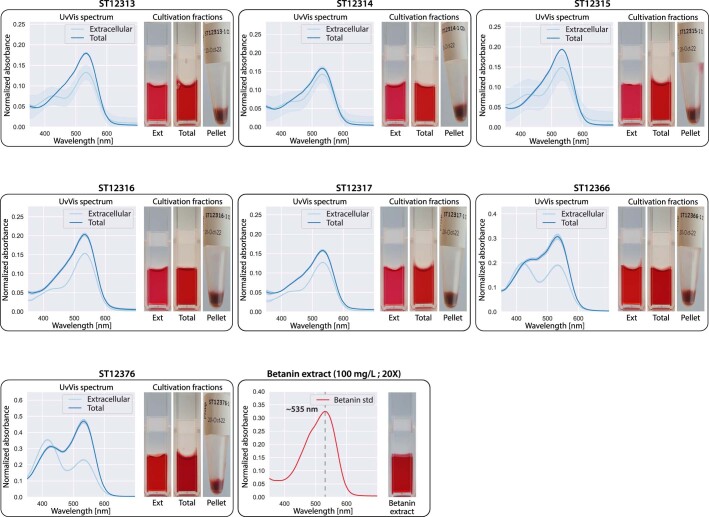
UV-vis spectra of the extracellular and total fractions from the betalain-producing *Y. lipolytica* strains (continued). UvVis spectra of 20X-diluted extracellular and total samples displayed on the left, with the corresponding non-diluted fractions displayed on the right. The pellet was derived from the 0.5 mL cell culture spun down to acquire the extracellular sample (non-disrupted). For comparison, the UV-vis spectra of 20X diluted betanin extract and a cuvette with non-diluted extract is displayed as the last figure. Cultivations were carried out in biological triplicate (n = 3), with the solid lines representing averages and shaded areas representing the standard deviations. Source data

To identify additional flux-controlling steps, we carried out sequential optimization campaigns of either the betanin precursor pathway(s) (l-tyrosine) or the heterologous pathway (pathway copy number) (Fig. 2b). Initially, we implemented a second copy of the betanin pathway in ST11193 and ST11664, hereby testing whether the activity of the heterologous pathway was efficiently driving betanin production. While a second pathway copy only marginally improved betacyanin production in the absence of *YlARO4*^*K221L*^ and *YlARO7*^*G141S*^ (ST11940), adding a second copy in a strain containing the feedback-insensitive alleles (ST11942) improved production 2.2-fold, resulting in 47.5 mg l^−1^ betacyanin (38.6 mg l^−1^ betanin, 8.9 mg l^−1^ isobetanin). Before initiating another round of metabolic engineering, we sought to probe the potential of further engineering the precursor supply. Here we found that while supplementing the mineral media (MM) with increasing concentrations of l-tyrosine did indeed improve betanin production (Extended Data Fig. 2), the conversion rate was quite poor, with 2 g l^−1^ l-tyrosine required to double betacyanin production (71.7 mg/ l^−1^ betanin, 17.5 mg l^−1^ isobetanin). Several studies have shown that improved chorismate production can be obtained in *Y. lipolytica* and *S. cerevisiae* by introducing additional copies of the pentafunctional aromatic polypeptide (*YlARO1*), the bifunctional chorismate synthase (*YlARO2*) and the DAHP synthase isoform (*YlARO3*)^25–27^. Accordingly, we combinatorically introduced copies of *YlARO1–3* in ST11942 and, in parallel, introduced an additional copy of the heterologous betanin pathway. While no combination of *YlARO1–3* led to considerable improvement, adding a third pathway copy (ST12310) increased betacyanin production 33% (52.5 mg l^−1^ betanin, 10.8 mg l^−1^ isobetanin)—indicating that considerable flux control still resided with the betanin biosynthetic enzymes. Ultraviolet–visible (UV–vis) spectra of the extracellular and total fractions for all engineered strains can be found in Extended Data Figs. 3 and 4.

### Eliminating side product formation improves betanin production

**Extended Data Fig. 5:**
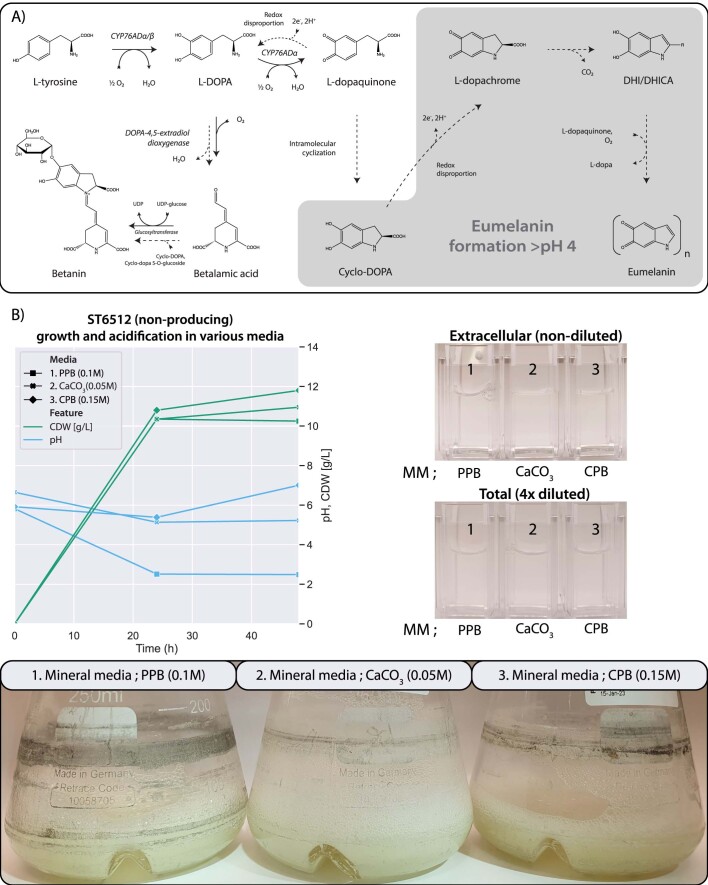
Effect of cultivation media pH on browning/eumelanin formation in non-producing *Y. lipolytica*. **a)** Proposed pathway for spontaneous eumelanin formation in connection to the heterologous betanin pathway. The betalain biosynthesis pathway shares many similarities to the well-described Raper-Mason-Prota pathway for melanogenesis^18^. In the absence of thiol-containing compounds, L-dopaquinone undergoes intramolecular cyclization to form cyclo-DOPA. Cyclo-DOPA is a pivotal intermediate in betanin biosynthesis – containing the hydroxyl group that upon glycosylation stabilizes the pigment molecule – but rapidly decomposes into the eumelanin precursors 5,6-dihydroxyindole (DHI, R = H) and 5,6-dihydroxyindole-2-carboxylic acid (DHICA, R=COOH) at pH values above 4^30^. The oxidative polymerization of DHI into eumelanin can effectively be mediated by L-dopaquinone. **b)** Non-producing *Y. lipolytica* cultivated for 48 h in three different mineral medias with varying buffering capacity (pH 6) containing 20 g/L D-glucose. Notably, mineral media buffered with potassium phosphate buffer (PPB) is unable to retain the target pH 6. Regardless of media pH, browning is not observed in non-producing *Y. lipolytica*. Cultivations were carried out in biological duplicate (n = 2). PPB: potassium phosphate buffer. CPB: citrate phosphate buffer. Source data

**Extended Data Fig. 6:**
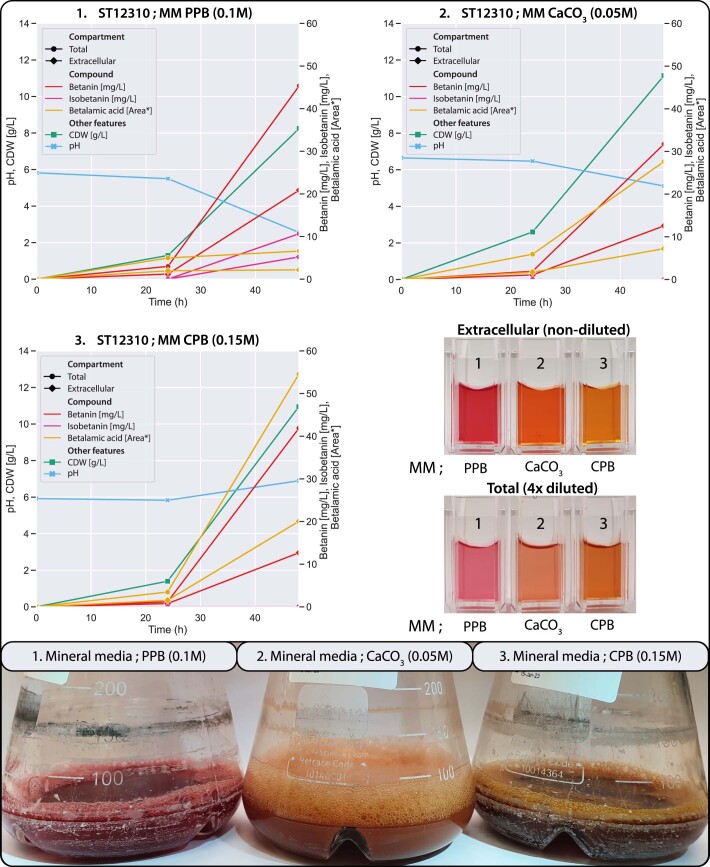
Effect of cultivation media pH on browning/eumelanin formation in ST12310 (with *4-HPPD*). ST12310 cultivated for 48 h in three different mineral medias with varying buffering capacity (pH 6) containing 20 g/L D-glucose. The browning effect appears to be correlated with the buffering capacity of the media – and by extension media pH. Consistent with cyclo-DOPA decomposition at pH above 4, browning was not observed in media buffered with potassium phosphate as the pH here quickly fell to ~2.5-3.0. In media buffered with calcium carbonate or citrate phosphate a large amount of betalamic acid / betaxanthin is produced, likely due to the unavailability of cyclo-DOPA at this pH. Cultivations were carried out in biological duplicate (n = 2). PPB: potassium phosphate buffer. CPB: citrate phosphate buffer. Source data

**Extended Data Fig. 7:**
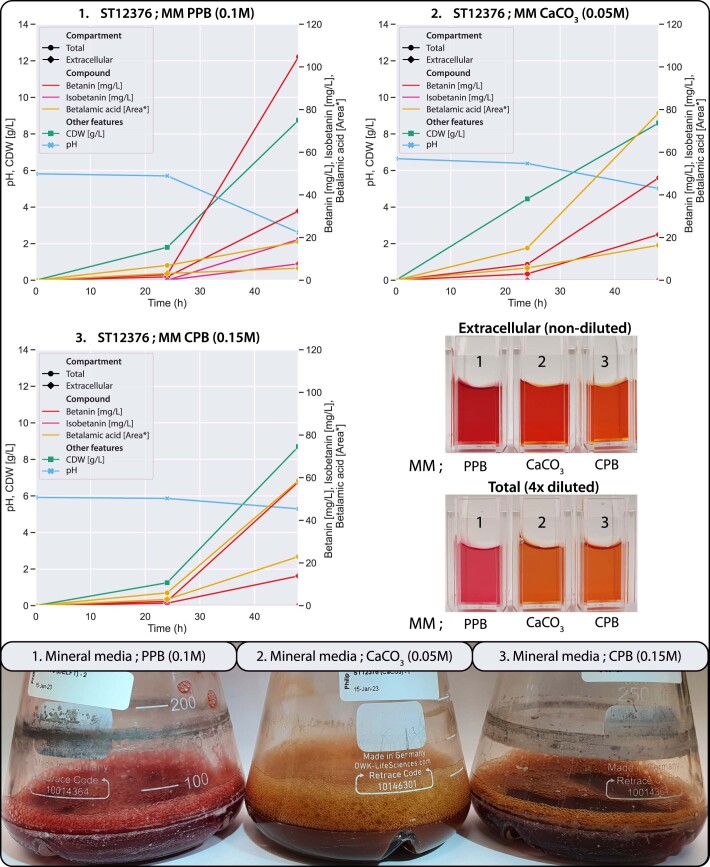
Effect of cultivation media pH on browning/eumelanin formation in ST12376 (*Δ4-hppd*). ST12376 cultivated for 48 h in three different mineral medias with varying buffering capacity (pH 6) containing 20 g/L D-glucose. The browning effect appears to be correlated with the buffering capacity of the media – and by extension media pH. Notably, the browning of the media occurs regardless of the presence or absence of *4-HPPD* – indicating that it is likely not due to pyomelanin formation. Additionally, even in *Y. lipolytica* strains engineered specifically for pyomelanin production (*4-HPPD* overexpressed), browning does not occur before 96 h^4^. Cultivations were carried out in biological duplicate (n = 2). PPB: potassium phosphate buffer. CPB: citrate phosphate buffer. Source data

**Extended Data Fig. 8:**
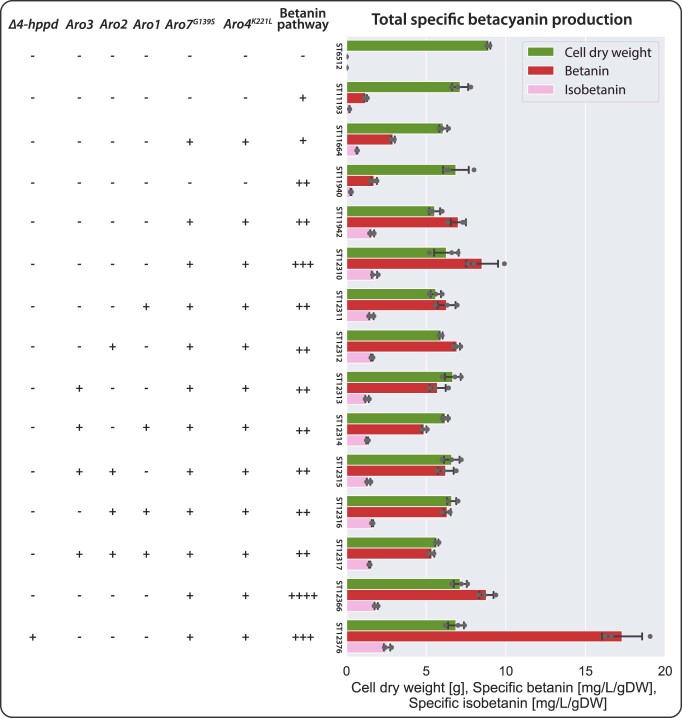
Cell dry weights and total specific betanin production. Cell dry weights and the specific betanin/isobetanin production in the lysed samples corresponding to the strains generated in Fig. 2. The best-performing strain (ST12376) has a ~30% reduction in growth compared to the non-producing parental strain (ST6512), but it grows similarly to the strain containing only one pathway copy (ST11193). Cultivations were carried out in biological triplicate (n = 3), with bars indicating averages and the error bars the corresponding standard deviations. Source data

We cultivated strain ST12310 in shake flasks in MM buffered with either potassium phosphate (0.1 M), calcium carbonate (0.05 M) or citrate phosphate (0.15 M). The cultivations buffered with calcium carbonate or citrate phosphate maintained a pH of around 5–6 as intended, while in the potassium phosphate buffer, the pH dropped to 2.5 towards the end of the cultivation. In cultivations with pH maintained at ~5–6, the medium turned brown and increased amounts of betalamic acid were detected (Extended Data Figs. 5 and 6). The browning of *Y. lipolytica* is often attributed to its l-tyrosine catabolism—resulting in the formation of melanins^28^. Here a tyrosine aminotransferases (*YlARO8*, *YlARO9*) convert l-tyrosine into 4-hydroxyphenylpyruvate, which is subsequently oxygenated by a 4-hydroxyphenylpyruvate dioxygenase (*Yl4HPPD*), yielding homogentisic acid (HGA). Once HGA accumulates sufficiently in the extracellular environment, it autoxidizes and polymerizes into a subclass of melanins called pyomelanin^28,29^. Accordingly, we disrupted *Yl4HPPD* in ST12310 as this has been shown to be an effective strategy for eliminating pyomelanin formation in *Y. lipolytica*^29^. Upon deletion of *Yl4HPPD*, we noticed that the resulting colonies (ST12376) were redder, indicative of increased betanin production; however, the browning persisted in the buffered media (Extended Data Figs. 5 and 7). Assessment of the betanin production revealed that disruption of *Yl4HPPD* increased betacyanin production 2.1-fold (118.1 mg l^−1^ betanin, 17.4 mg l^−1^ isobetanin) (Fig. 2b and Extended Data Fig. 8). In comparison, further enhancing flux through the heterologous pathway by integrating an additional pathway gene copy in ST12310 (ST12366) resulted in only 19% titre improvement (62.4 mg l^−1^ betanin, 13.2 mg l^−1^ isobetanin). Probably, considerable flux was diverted towards HGA production rather than l-tyrosine formation. We hypothesize that the browning exclusively observed in media with enhanced buffering capacity was due to eumelanin formation (Fig. 1), resulting from cyclo-DOPA and l-dopachrome decomposition at pH > 4 (refs. ^30,31^).

### Fed-batch fermentation enables gram-scale betanin production

**Fig. 3:**
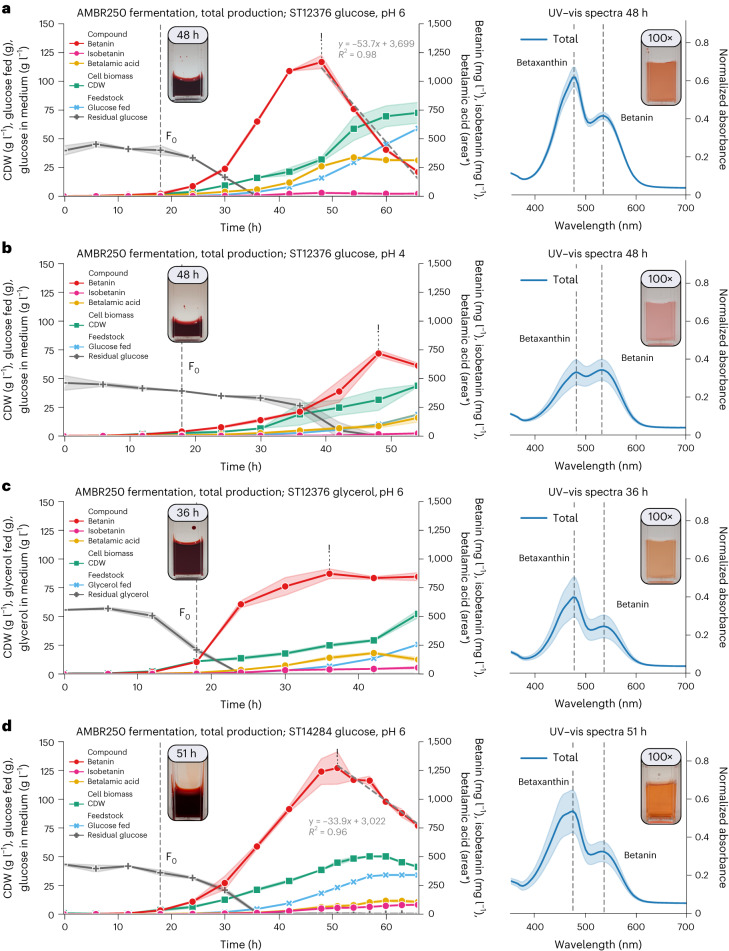
Fed-batch fermentations in bioreactor. **a**–**c**, Bioreactor cultivations with ST12376 performed in duplicate with either d-glucose as feedstock (pH 6; **a**), d-glucose as feedstock (pH 4; **b**) or glycerol as feedstock (pH 6**; c**). **d**, ST14284 was fermented with d-glucose as feedstock (pH 6). Solid lines indicate the average from both bioreactors, and shaded areas represent the corresponding standard deviations. Dashed grey lines in the fermentation graphs of **a** and **d** represent betanin degradation kinetics. ‘area*’ indicates HPLC quantification by UV-vis spectra, without internal standards. Illustrations of physiologically relevant parameters from the fermentations can be found in Extended Data Fig. 9. As whole-cell samples were frozen directly after sampling, only the total betacyanin production could be accurately determined. A picture of undiluted sample at peak (‘!’) betanin production is shown in the fermentation graph. The corresponding diluted sample (100×) and UV–vis spectra (400×) can be found to the right of the fermentation graphs. F_0_, feed initiation. Source data

To assess the potential of fermentation-based betanin production, controlled fed-batch fermentations were performed with the optimized strain (ST12376) in MM with either glucose or glycerol as the sole carbon source. Fermentations with glucose were carried out at pH 4 and pH 6 to evaluate the effect of pH on product degradation and by-product formation. The highest betanin titre was obtained in fermentations with glucose at pH 6. Here we produced 1,197 ± 42 mg l^−1^ betacyanin (1,168 mg l^−1^ betanin, 29 mg l^−1^ isobetanin) in 48 h (Fig. 3a). The titre obtained at pH 4 was lower, with only 734 ± 22 mg l^−1^ of betacyanin (718 mg l^−1^ betanin, 15 mg l^−1^ isobetanin) produced in 48 h, but the fermentation broth contained noticeably less betaxanthins (*λ*_max_ = 475 nm) and betalamic acid (Fig. 3b). The pH optimum of betanin is reported to be between pH 4 and pH 6 (ref. ^22^); however, cyclo-DOPA—key to betanin formation—is prone to degradation at pH values above 4 (ref. ^30^), explaining the reduced betaxanthin formation. When glycerol was used as feedstock, production peaked at 911 ± 31 mg l^−1^ betacyanin (872 mg l^−1^ betanin, 39 mg l^−1^ isobetanin) already after 36 h (Fig. 3c). Noticing betanin degradation shortly after peak production, we hypothesized that native *Y. lipolytica* beta-glucosidases were responsible. We identified and disrupted 14 putative beta-glucosidases in wild-type *Y. lipolytica* and performed a degradation assay (Extended Data Fig. 10), which led to the identification of two beta-glucosidases (YALI1_B18845g and YALI1_B18887g) that seemed highly involved in betanin deglycosylation. Disruption of these two beta-glucosidases in our production strain (resulting in ST14284) allowed us to retain 3.6-fold more betanin at 66 h (Fig. 3d). In addition, we achieved a record betacyanin titre of 1,326 ± 148 mg l^−1^ (1,271 mg l^−1^ betanin, 55 mg l^−1^ isobetanin) in 51 h. This shows that high-level betanin production can be achieved in engineered *Y. lipolytica*, but additional strain and process optimization is required to further improve production and eliminate by-product formation.

### LCA of fermentation-based betanin production

**Fig. 4:**
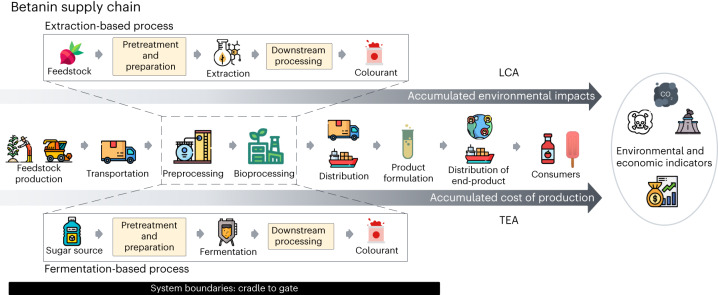
Overview of the betanin supply chain and assessed system boundary (cradle to gate) for LCA and TEA. Comparison of the supply chain of extraction-based betanin production (top) with that of the proposed fermentation-based process (bottom). Credit: icons, Flaticon.com.

Environmental sustainability performance and economic viability of the proposed fermentation process compared with those of the traditional extraction-based method for betanin production were assessed using LCA and TEA, respectively. A systems engineering approach was used to quantify environmental impacts and economic costs across a cradle-to-gate boundary by applying LCA and TEA (Fig. 4). LCA and TEA are strategic tools in process design used to guide innovation from the inception point^32^. In research and development, applying TEA allows foreseeing economic pitfalls and lowers risk related to low-maturity technology, thus enabling the development of relevant process design parameters^33^. The assessment provides insight into the potential of optimizing the process to realize an economically competitive product. Meanwhile, LCA identifies hotspots for improving the environmental sustainability of the process^34^.

**Fig. 5:**
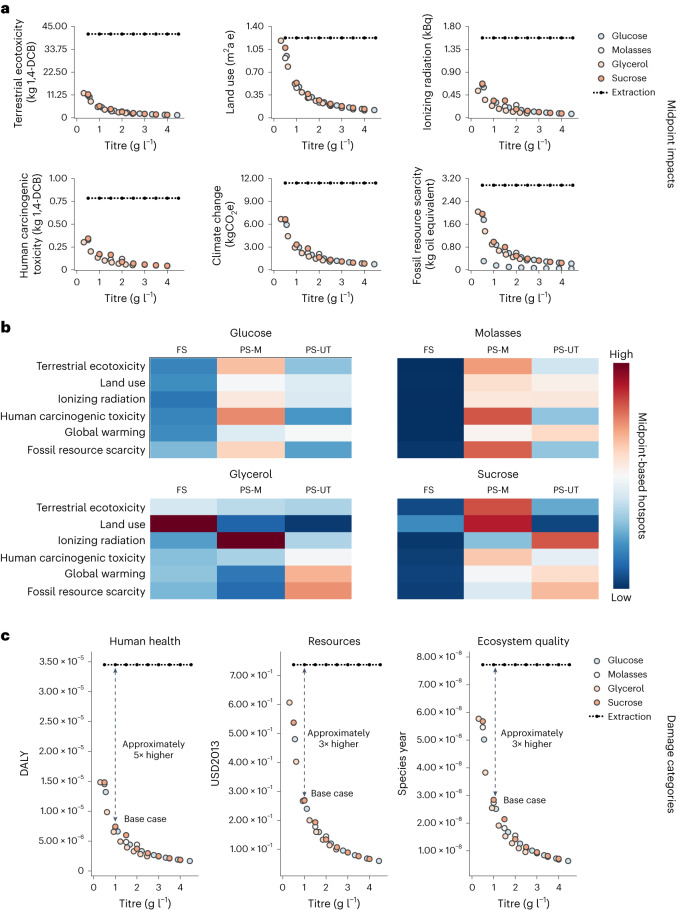
LCA results for fermentation-based betanin production. **a**, Midpoint results of relevant impact categories following the ReCiPe 2016 (H) methodology, comparing the fermentation-based process with the extraction-based process. Dicholorobenzene (kg 1,4-DCB) is used as a reference unit for toxicity, area in square metres multiplied by years occupied equivalent (m^2^a e) is a reference unit for land use and ionizing radiation is measured in kilobequerel of ^60^Co equivalent (kBq). Climate change measured in kgCO_2_-equivalent (kgCO_2_e). **b**, Hotspot analysis for each feedstock, where ‘red’ indicates higher impact and ‘blue’, lower impact. **c**, Sensitivity analysis for varying titre with each feedstock given in end-points, where ‘DALY’ refers to disability-adjusted life years as a reference unit for human toxicity, ‘USD2013’ is the reference unit for resource scarcity and ‘Species year’ refers to local species loss integrated over time as a reference unit for ecosystem quality. Base-case titre is indicated with feedstock names. Source data

The ReCiPe 2016 methodology, using the hierarchist (H) perspective, was applied to quantify the environmental impacts at both midpoint and end-point levels^35^. The methodology section describes all the assumptions for the LCA study. The LCA results for microbially produced betanin from different feedstocks, including glucose, molasses, glycerol and sucrose, can be found in Fig. 5. The LCA midpoint results indicate that the categories most distinct from beetroot extraction were terrestrial ecotoxicity, land use, ionizing radiation, human carcinogenic toxicity, global warming and fossil resource scarcity. The midpoint dataset can be found in Supplementary File 1. Five scenarios were formulated based on variations from a −50% base-case titre to a fourfold increase—in 50% stepwise increments to assess changes in environmental performance against the extraction-based process. The midpoint results for the different feedstocks and titre scenarios show that fermentation-based betanin processes have a superior environmental sustainability performance compared with the extraction-based process (Fig. 5a). In contrast to the other midpoints, impacts on land use were similar between the extraction process and low-titre fermentation scenarios (although slightly favouring fermentation). This outcome is due to the maltodextrin carrier agent in the E162 formulation and carbon source requirement in the fermentation scenarios. The contribution of process stages to environmental impacts was analysed using midpoint-based hotspots (Fig. 5b). The processing material (PS-M) flows section, which includes material inputs required by the process, was observed as the main driver of impacts on most categories across the different feedstocks. Overall, impacts from feedstock (FS) production were not meaningful compared with the PS-M stage contribution, except for the glycerol process. When glycerol was used as feedstock, impacts on land use and terrestrial ecotoxicity increased compared with the other feedstocks, driven by rapeseed oil production in the upstream processing. Process utilities (PS-UT), primarily related to electricity consumption, contributed the most to climate change impacts in all fermentation processes.

The midpoint impacts reveal that the fermentation process might benefit from process optimization. Therefore, a sensitivity analysis was performed to estimate the improvements in the environmental performance of fermentation-based betanin, as a function of titre (Fig. 5c). This approach accounts for early-stage processes and inherent uncertainty issues and robustness of the LCA model. Here the results indicate that optimizing the fermentation titre could reduce the environmental impacts substantially across all categories, and using glucose as feedstock showed the best performance at 4.4 g l^−1^ betanin titre. Conversely, glycerol scenarios showed the worst profile among the evaluated feedstocks, although titre optimization could lower the impacts overall. Sucrose and molasses showed a similar performance, somewhere in between the best (glucose) and worst (glycerol) cases. Moreover, the results revealed that the impacts of the traditional betanin extraction process are approximately five times higher for human health, and three times higher for both ecosystem quality and resources, compared with the impacts of fermentation. Hereby, the proposed biotechnological process, which is far from mature, already outperforms the traditional betanin production method. This sensitivity analysis was complemented by assessing production locations for Germany, Brazil and China (Supplementary File 1). Glucose fermentation stands out as the best-performing scenario, and increasing titre will further benefit the environmental profile.

### TEA of fermentation-based betanin production

**Fig. 6:**
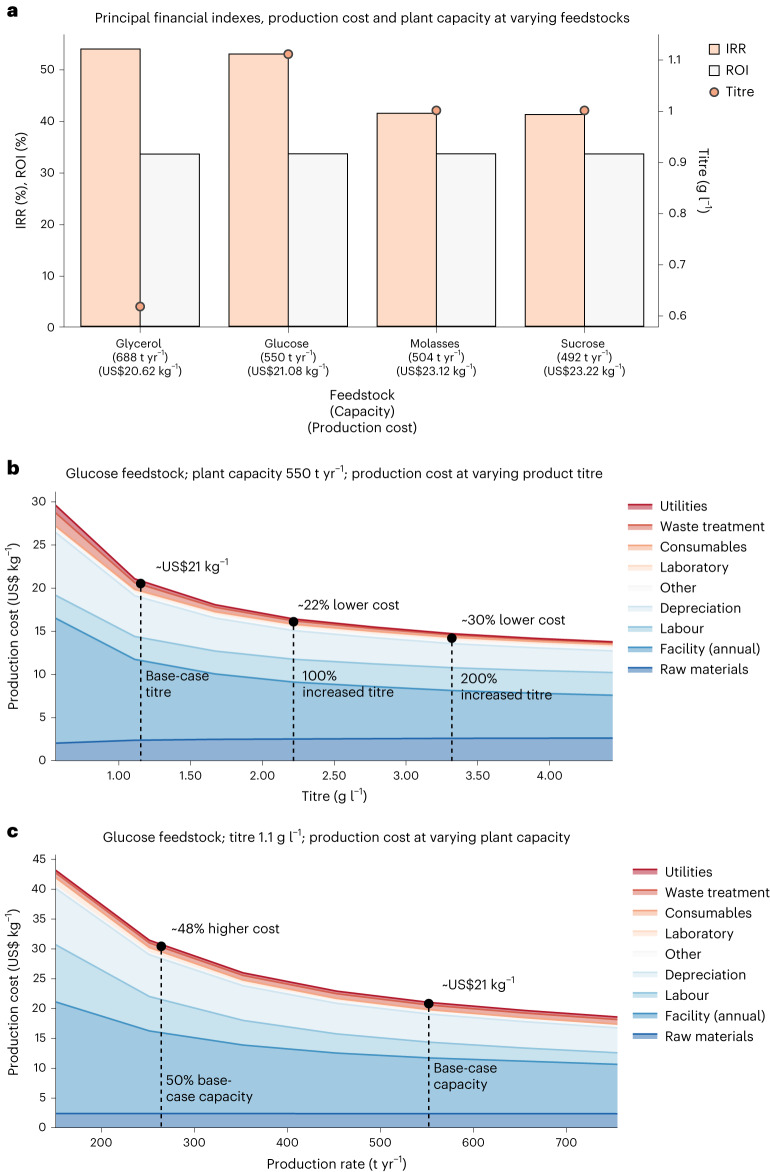
TEA of fermentation-based betanin production using *Y. lipolytica*. **a**, Principal financial indexes and base-case titre for the four feedstock scenarios in the fermentative betanin-colourant production. A payback period of 3 years, selling price of US$34.75 kg^−1^ E162 and production capacity of less than 755 t yr^−1^ were the three major constraints applied. The base-case production cost and calculated capacity are indicated on the *x*-axis. **b**, Sensitivity and breakdown of the production cost as a function of fermentation titre for the scenario using glucose as the feedstock. The titre was varied from 0.56 g l^−1^ to 4.44 g l^−1^. **c**, The cost of betanin production is illustrated as a function of the production capacity with glucose as the feedstock. Source data

While LCA results indicated that fermentation-based betanin overall has a superior environmental sustainability profile compared with betanin from the extraction-based route, this technology will have little societal impact if the microbially produced betanin cannot compete in the current food colourant market owing to a high production cost. We evaluated the economic performance of a fermentation-based process for betanin production using four feedstocks and estimated the cost of production of 1 kg of E162 colourant at commercial production scale. E162 is the common commercial form of betanin as a food colourant, formulated with maltodextrin as the carrier agent, containing a minimum of 0.4% betanin to be compliant with European Union regulations^3^. The production process consists of a fermentation step (upstream) and a downstream step, the latter comprising a biomass separation phase, an evaporation phase and a drying step with carrier agent addition (Supplementary File 1 and Supplementary Fig. 4). A baseline scenario was set for each feedstock with a target payback period of 3 years, a limit in production capacity of 755 t yr^−1^ and an E162 selling price of US$34.75 kg^−1^. Financial parameters and production costs were calculated across several feedstocks, namely, glucose, glycerol, sucrose and molasses. The titres selected for glucose and glycerol were 1.1 g l^−1^ and 0.617 g l^−1^, respectively, corresponding to the concentration of betanin achieved at 42 h and 24 h (at peak productivity), as indicated in Fig. 3a,c. For sucrose and molasses, the titre was arbitrarily set at 1 g l^−1^, reached in 42 h (comparable to glucose). Here we found that glycerol was the best financially performing feedstock, with an internal rate of return (IRR) of 53.7%, enabling betanin colourant (E162) production at US$20.62 kg^−1^. This, however, is tied to an overall higher production capacity of 688 t yr^−1^ (a 40% increase compared with the worst-performing scenario; Fig. 6a). In addition, we found that the costs are distributed equally between upstream and downstream. The downstream cost ranges from 50% of the total production cost with glycerol as feedstock (baseline scenario) to 51.6% when molasses is used as the carbon source.

Hereafter, we investigated the impact on production cost as a function of titre when using glucose as feedstock. Scenarios were simulated by varying from −50% to 400% of the baseline titre (1.1 g l^−1^ for glucose; Fig. 6b). Here we found that increasing the titre twofold results in cost reductions of ~22%, while increasing the titre threefold would reduce production costs by ~34.5%. Such improvements in betanin titre are easily achievable by further metabolic engineering or fermentation optimization, and cost reductions appear to be largely invariant to feedstock (Supplementary File 1 and Supplementary Fig. 7). As the market volume and production capacity estimation are highly uncertain, we lastly investigated the colourant production cost as a function of yearly betanin productivity using glucose as feedstock (Fig. 6c). Here the economies-of-scale effect is apparent, with production costs increasing 48% at half the baseline production capacity while reducing ~11% with an increase of ~35% in productivity. The selling price of betanin colourant is also uncertain, varying between US$8.5 kg^−1^ and US$61 kg^−1^ (Supplementary File 1); however, the sensitivity analysis indicates that for a financially viable venture, the minimum expected selling price must be at least US$21.5 kg^−1^ formulated betanin product.

## Discussion

With growing consumer concerns about the sustainability and safety of synthetic food colours, natural colours extracted from plants are increasingly used in the food industry. While plant cultivation for the extraction of natural dyes is land and resource demanding, fermentation-based processes leveraging genetically modified microbes are promising alternative sources of food additives. To this end, we engineered the yeast *Y. lipolytica* to produce red beet betalains via fermentation at gram scale in bioreactors—resulting in an ~42-fold improvement compared with previous attempts in *S. cerevisiae*^13–15^. The key metabolic engineering strategies that improved betanin production were the screening of betanin biosynthetic enzymes and optimizing their copy number, improving the l-tyrosine supply, eliminating the side flux to HGA by deleting the *4-HPPD* gene and reducing betanin degradation by deleting beta-glucosidases. *Y. lipolytica* is an attractive host for the scale-up of biotechnological processes as it is Crabtree negative, genetically stable and robust to stress factors unavoidable in large-scale fermentations^16^. While in this work we focused on betanin, the structural diversity of the naturally occurring red–purple betalains is large, with additional decorations of betanin via acylation or further glycosylation leading to colour variants with different hues and stability properties^8,13^. This presents an opportunity to expand our work to the biotechnological production of other plant betalains that currently are not commercially available owing to their low native content in the respective plants^36^.

In this work, we also assessed the environmental impacts and economic outlook of our fermentation process to guide further optimization. This initial assessment ensures that resources are not wasted on developing processes that, even when fully optimized, would never be able to compete on sustainability or economics with the process they were meant to replace. As is unfortunately common, sustainability and economics often do not align, as we observed when modelling the feedstock scenarios. *Y. lipolytica* can use various feedstocks, such as sugars and glycerol^16^, and while LCA suggested that glycerol is a less sustainable feedstock among those modelled, TEA indicated that it might be one of the more profitable ones. That said, the main driver for improving the sustainability and economics of our fermentation-based process, as revealed by LCA and TEA, would be increasing the betanin titre and productivity. A mere twofold improvement in betanin titre, easily achievable by further strain engineering or process optimization, considerably improved the modelled sustainability profile and economics.

## Methods

### Synthetic genes and DNA materials

Heterologous genes were synthesized as synthetic gene strings by GeneArt (Thermo Fisher Scientific) and codon optimized for *Y. lipolytica* using the codon-optimization tool provided by GeneArt. The corresponding amino acid and nucleotide sequences can be found in Supplementary File 1. Single-stranded oligonucleotides were obtained from Integrated DNA Technologies, and a list of all oligonucleotides used in this work can be found in Supplementary Data 1.

### Strains and media

All *Y. lipolytica* strains generated in this study were derived from ST6512, a W29/CLIB89 (NRRL Y-63746) strain containing a Cas9 expression cassette in the *KU70* locus^37^. Depending on the strain design and cultivation purpose, yeast strains were maintained in media prepared according to the following recipes. Standard liquid yeast peptone dextrose medium was prepared with 10 g l^−1^ yeast extract and 20 g l^−1^ peptone. MM buffered with potassium phosphate (KH_2_PO_4_) was prepared using 7.5 g l^−1^ (NH_4_)_2_SO_4_, 14.4 g l^−1^ KH_2_PO_4_ and 0.5 g l^−1^ MgSO_4_7H_2_O, and the appropriate growth factors (trace metals and vitamins), and adjusted to pH 6.0 with NaOH^38^. Unless stated otherwise, cultivations for betalain production were conducted using MM buffered with potassium phosphate, as we found this resulted in the highest betanin titres (Extended Data Figs. 5–7). MM buffered with CaCO_3_ was prepared identically but was supplemented with 5 g l^−1^ of CaCO_3_. MM buffered with citrate phosphate was likewise prepared identically, but the 14.4 g l^−1^ KH_2_PO_4_ was replaced with 16.5 g l^−1^ Na_2_HPO_4_ and 8 g l^−1^ citric acid. Unless stated otherwise, separately heat-sterilized d-glucose was added to all cultivation media to reach a concentration of 20 g l^−1^. When required, media were supplemented with the antibiotic nourseothricin (250 mg l^−1^) for selection of the guide RNA (gRNA) vector or hygromycin B (400 mg l^−1^) for selection of gene disruptions. Unless stated otherwise, yeast strains were cultivated at 30 °C and 250 rpm. *Escherichia coli* DH5α was used for all plasmid cloning and propagation. *E. coli* strains were grown in Luria–Bertani media supplemented with 100 mg l^−1^ ampicillin. Agar plates were prepared using the media described above supplemented with 20 g l^−1^ agar.

### Plasmid and strain construction

Plasmid constructions and gene insertions were performed in accordance with the EasyCloneYALI toolkit^39^. USER-compatible biobricks, encoding promoters or genes of interest, were PCR amplified with primers containing the uracil base, and assembled with PCR-linearized EasyCloneYALI integration vectors in *E. coli*^17^. Integration vectors generated for gene insertions were sequence verified using Sanger sequencing (Eurofins Genomics). Gene disruptions were mediated by unassisted homologous recombination of repair templates containing a hygromycin marker flanked by 500–600 bp homology arms targeting the upstream and downstream of the gene of interest. Here the upstream and downstream homology arms were PCR amplified, generating USER overhangs compatible with a hygromycin cassette flanked by *loxP* sites (Cre recombinase recognition elements). The hygromycin-containing repair template was looped out via the episomal expression of the site-specific Cre recombinase, and hygromycin-sensitive colonies were identified via replica plating on non-selective and selective media. Alternatively, gene disruptions were carried out marker-free by generating repair templates as described above, but without the hygromycin cassette, and assisted with gRNAs cloned in accordance with the EasyCloneYali toolkit^39^. Yeast transformations were carried out according to the lithium acetate method^39,40^.

### Small-scale cultivations

To assess small-scale betalain production, a single-colony pre-culture was prepared by inoculating cells from a glycerol stock in 2 ml MM. The strains were incubated for 48 h in 24 deep-well plates with air-penetrable lids (EnzyScreen). Hereafter, pre-cultured cells were inoculated into 2 ml of fresh MM (pABA^−^)—a modified mineral medium without *para*-aminobenzoic acid, which was excluded owing to this amino acid’s ability to condense with betalamic acid, interfering with betaxanthin quantification—to a starting optical density (OD_660_) of 0.1 and incubated for 48 h at 30 °C with shaking at 250 rpm. ODs were measured at 660 nm owing to betanin’s absorbance spectrum (*λ*_max_ = 535 nm) overlapping to a larger extent at 600 nm. After 48 h of cultivation, OD_660_ and betaxanthin fluorescence (excitation, 463 nm; emission, 512 nm) were measured in a plate reader (BioTek Elx 8089). In addition, 0.5 ml of the cultivation broth was spun down (11,000 × *g*, 5 min) in pre-dried and pre-weighed 1.5 ml Eppendorf tubes. The supernatant was analysed for extracellular betalain content; the pellet was washed in phosphate-buffered saline (pH 7.5) and then dried for 48 h at 60 °C. Once dry, the cell dry weight (CDW) was determined as the difference between the pre-dried Eppendorf tube and the dried cell pellet. To assess the total betalain content, 1 ml of cultivation broth was transferred to a 2 ml screw-cap microtube containing 0.5 ml acid-washed glass beads (425–600 μm) and the cells were lysed using the Precellys 24 homogenizer (Bertin Technologies). After cell disruption, the debris was spun down (11,000 × *g*, 10 min) in a pre-cooled (4 °C) centrifuge and the betalain content in the supernatant analysed. For both the extracellular and total samples, the betaxanthin fluorescence and betacyanin fluorescence (excitation, 521 nm; emission, 575 nm) were measured, in addition to their UV–vis spectra (BioTek Elx 8089). For l-tyrosine supplementation, a 50 g l^−1^ l-tyrosine stock was made in 1 M HCl owing to the poor solubility of this amino acid in water, and the appropriate amount was added to the MM. The pH was rebalanced by adding equimolar 1 M NaOH. For the betanin deglycosylation assay, pre-cultured cells were prepared as described above and inoculated into 2 ml MM supplemented with 100 mg l^−1^ betanin from red beet extract. Samples were collected after 24 h, and extracellular betanidin was quantified via high-performance liquid chromatography (HPLC).

### Fed-batch fermentations in bioreactor

To prepare seed cultures for fed-batch fermentations, *Y. lipolytica* ST12376 or ST14284 was streaked from cryostocks onto yeast peptone dextrose agar plates and incubated at 30 °C for 48 h. A single colony was then inoculated into 2 ml of corresponding batch MM in a 13 ml pre-culture tube and incubated at 30 °C with shaking at 250 rpm for 24 h. Next, a baffled shake flask containing 50 ml of corresponding MM was inoculated to an initial OD_660_ of 0.1 and incubated at 30 °C with shaking at 250 rpm for another 24 h. The flasks’ contents were centrifuged for 5 min at 5,000 × *g*, washed twice in MM and concentrated to a 5 ml volume. This concentrated cell suspension was used to inoculate the bioreactors to an initial OD_660_ of 0.1.

**Extended Data Fig. 9:**
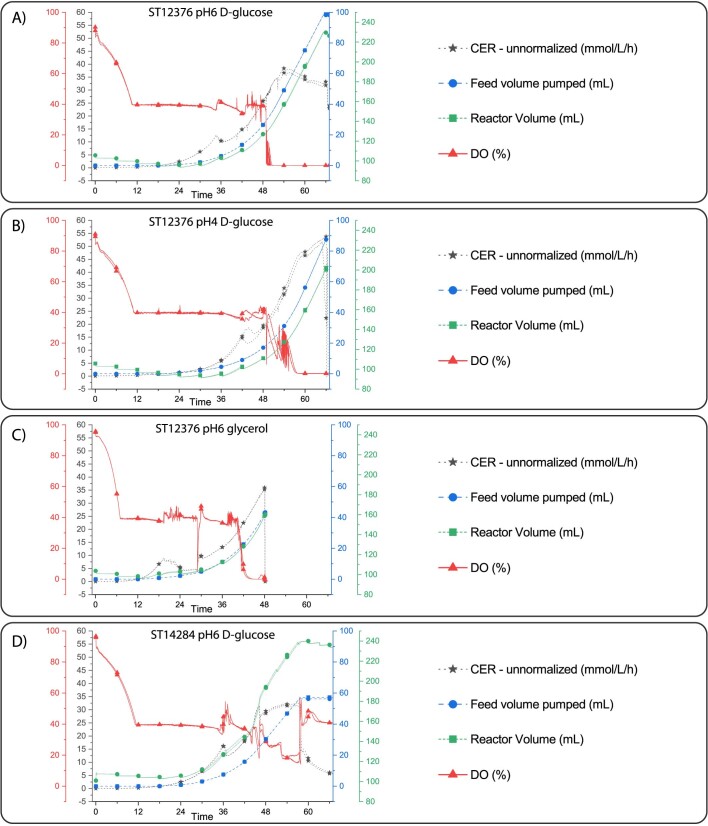
Relevant parameters from fed-batch fermentations. Carbon Evolution Rate – CER (mmol/L/h), Feed volume (mL), reactor total volume (mL) and Dissolved Oxygen – DO (%) from **a)** ST12376 fermentation with glucose at pH 6, **b)** ST12376 fermentation with glucose at pH 4, **c)** ST12376 fermentation with glycerol at pH 6, and **d)** ST14284 fermentation with glucose at pH 6. CER and D.O. from 2 independent biological replicates show that replicates were not physiologically different from each other. CER drop at **a**, 36 h **b**, 42 h **c**, 24 h and **d**, 36 h coincide with glucose limitation, when all residual glucose was consumed. Exponential fed-batch at 0.1 h^−1^ rapidly lead to carbon and oxygen limitations after 48 h, for glucose fed-batch, and after 40 h, for glycerol fed-batch, which may have negatively impacted the production of betanin. Source data

Four exponential fed-batch fermentations in duplicate were carried out in single-use 250 ml reactors using the AMBR250 system (Sartorius AG). Medium compositions and detailed operational fermentation parameters can be found in Supplementary Data 2 along with the raw online data (Extended Data Fig. 9).

Samples (2.5 ml) were automatically collected by the AMBR250 robotic arm every 6 h and immediately frozen at −14 °C. For ST14284, samples were taken every 3 h after 48 h to improve resolution and better assess the degradation profile. Betaxanthin quantification was not the goal of the fermentation, so *para*-aminobenzoic acid was included in the bioreactor media. To prevent possible eumelanin formation and browning of the media, or betanin degradation upon carbon depletion, feeding was initiated before carbon depletion, which differs from common practice.

### Analytical methods

To quantify betanin and isobetanin, the cultivation and fermentation supernatant was analysed via HPLC using the Dionex Ultimate 3000 HPLC system (Thermo Fisher Scientific). For HPLC analysis, 10 μl of sample was injected into a Zorbax Eclipse Plus C18 reverse-phased column (particle size, 3.5 μm; pore size, 95 Å; 4.6 × 100 mm). The column oven temperature was set to 30 °C, and the flow rate was 1.0 ml min^−1^. Solvent A was water plus 0.1% formic acid, and solvent B was 100% acetonitrile. The solvent composition was initially set to A = 98.0% and B = 2.0%, and kept steady for 2 min. Hereafter, the solvent composition was adjusted following a linear gradient, until reaching A = 90.0% and B = 10.0% at 5.0 min. Then, the slope of the gradient was decreased until reaching A = 85.0% and B = 15.0% at 8.0 min. The column was then flushed by setting A = 2.0% and B = 98% at 8.2 min. These conditions were kept steady for 9.5 min and were then returned to the initial conditions of A = 98.0% and B = 2.0% at 9.6 min, at which point the solvent composition remained unchanged until the end of the run at 11.5 min. Betanin and isobetanin were detected at a wavelength of 540 nm, and their retention times were 5.68 min and 6.10 min, respectively. The UV–vis detector captured data at 390 nm, 410 nm, 480 nm and 540 nm. As pure betanin standard is not commercially available, peaks corresponding to betanin and isobetanin were identified by comparison to prepared red beet extract diluted with dextrin (product identification: 901266-5 G)^41^. As reported^15^, this commercial red beet extract contains an equimolar ratio of betanin and isobetanin, and by using the Beer–Lambert equation assuming a molar extinction coefficient of *ε* = 6.5 × 10^4^ M^−1^ cm^−1^ for betanin, we calculated that 1 g l^−1^ of this extract contains 0.837 mg l^−1^ of betanin and isobetanin. The Chromeleon 7 software (Thermo Fisher Scientific) was used to analyse HPLC results and generate standard curves.

For identification of anthranilate–betaxanthin and betanidin, the supernatant was subject to untargeted LC–UV–tandem mass spectrometry (MS/MS) analysis. Here a 1 μl sample was injected in an ultra-high-performance liquid chromatography (Ultimate 3000) system coupled to a diode array detector (heated electrospray ionization) Orbitrap Fusion mass spectrometer (Thermo Fisher Scientific). Chromatographic separation was achieved using a Waters ACQUITY BEH C18 guard column (2.1 mm × 100 mm, particle size 1.7 μm) kept at 40 °C with a flow rate of 0.35 ml min^−1^. Solvent A was MilliQ water plus 0.1% formic acid, and solvent B was 0.1% formic acid. The solvent composition was, initially, A = 98% and B = 2% and kept steady for 0.8 min. Hereafter, solvent composition followed a linear gradient to A = 95% and B = 5% for 3.3 min. Then, solvent composition was changed to A = 0% and B = 100 over 10 min and held steady for 2 min before returning to the initial conditions. Re-equilibration time was 2.7 min. The diode array detector settings were as follows: data collection rate, 10 Hz; wavelength range, 190–600 nm; and bandwidth, 2 nm. The MS/MS measurement was done in positive-heated electrospray ionization mode with a voltage of 3,500 V acquiring in full MS/MS spectra (data-dependent acquisition-driven MS/MS) with a m/z range of 70–1,000. The MS1 resolution was set at 120,000, and the MS2 resolution was set at 30,000. Precursor ions were fragmented by stepped high-energy collision dissociation.

### Sustainability assessment

A sustainability assessment of the fermentation-based betanin production was performed by quantifying its economic and environmental performance. Thus, LCA^42^ and TEA^43^ methodologies were applied, and the results were benchmarked with the current extraction-based process. The fermentative betanin process was designed and evaluated for four feedstocks (glucose, sucrose, molasses and glycerol). Data used in TEA and LCA are based on the information taken from experiments and literature. Scaling-up principles and fundamental assumptions were applied, and computer-aided process engineering tools were used along with engineering rules of thumb. Import–export data prices from recent years were used to retrieve the commodity prices of the main raw materials using the online database Import Genius. The extraction and fermentation processes’ mass and energy balances were modelled using SuperPro Design software, generating inventories for the LCA. The economic assessment was performed using the built-in functions in SuperPro Design and manually customizing the equipment cost estimation functions according to in-house experience, engineering rules of thumb and region-specific financial parameters (Supplementary File 1). The LCA was completed in SimaPro software (under license). All the inputs and assumptions are described in detail in Supplementary File 1.

### Description of process simulation

The conventional plant-based extraction process was modelled assuming a 0.07% betanin content in beetroot, which is processed through a sequence of upstream and downstream units following the setup given in a previous study^7^ and confirmed by the European Food Safety Authority (EFSA)^3^. To attain a powder form, betanin is sprayed on a carrier agent (maltodextrin) to achieve the target 0.4% wt./wt. formulation. This product formulation was set according to minimum limits given by EFSA^3^ for E162. The fermentation process was modelled using a modified *Y. lipolytica* (NRRL Y-63746) strain as the host. As no data on the fermentative betanin downstream process was found, this section was modelled on extraction-based downstream steps. The equipment costs were calculated using the parameters provided in a previous study^44^. The production capacity in the base-case scenario ranges from 151 t yr^−1^ to 755 t yr^−1^ (depending on feedstock) and was derived targeting a positive net present value and a payback period of 3 years. These targets are reasonably expected in innovative biotechnology projects at a low maturity level^45^. The upper and lower production capacity thresholds were calculated from the market analysis as described in Supplementary File 1. Lastly, several TEA parameters were regionalized including the electricity price, labour cost, carbon dioxide emission costs and the major financial assumptions, namely, inflation, interest rate and corporate taxation assuming Germany as a base-case scenario. The full dataset used for the TEA and associated calculations can be found in Supplementary File 1.

### LCA

The LCA is a standardized methodology based on International Organization for Standardization (ISO) 14040 and 14044 (ref. ^46^) standards. The LCA consists of four main phases: (1) goal and scope, (2) life cycle inventory (LCI), (3) life cycle impact assessment (LCIA) and (4) interpretation of results^47^. The software SimaPro and the Ecoinvent 3.8 database (Supplementary Data 3) were used to perform the assessment. The LCA input data can be found in Supplementary File 1 and Supplementary Data 3.

### Goal and scope definition

The goal is to assess and compare the environmental sustainability performance of fermentation-based betanin production against the traditional extraction process. The functional unit was defined as 1 kg of colourant, with a betanin concentration of 0.4%. In this formulation, the product is a natural colour authorized as a food additive in the European Union in accordance with Annex II to Regulation (EC) No. 1333/2008 (ref. ^3^). The plant is assumed to be in northern Germany. This LCA study covers a cradle-to-gate system boundary, including the following major stages: biomass production and resource extraction, betanin synthesis and product formulation. The gate-to-gate stage, which refers to the unit process from the reception of the raw materials to the completion of the production process, is divided into (1) materials and processing and (2) utilities. End-of-life management is not included as the product is physically integrated into other supply chains, unidentifiable due to further physical–chemical processing^48^.

### LCI

Various sources of LCI information were used to populate inventory flows. Information sources included literature, simulation results, experiments and industrial reports, among others^49^. The inventory contains the input and output exchanges within the boundaries defined for the product system. The main source of background data is the Ecoinvent 3.8 database (Supplementary Data 3), and the foreground data are provided by process simulation. The inventory flows were normalized based on the defined functional unit. Linking process design and simulation with LCA methodology was crucial to estimate the environmental sustainability performance of these processes^50^.

### LCIA

The selected impact assessment methodology was the ReCiPe 2016 Midpoint. The LCIA was performed with SimaPro v.8.5. The evaluation covered all impact categories included in the ReCiPe 2016 methodology, using H. Evaluated midpoint impact categories included climate change, ozone depletion, ionizing radiation, fine matter, eutrophication, human toxicity, land use and water consumption, among others^35^. Furthermore, end-point categories consist of human health, ecosystems and resources. Hotspot analysis in this work covers the most significant impacts related to impact magnitude and process stage.

### Interpretation of results

Different scenarios were formulated to evaluate the robustness of LCA results owing to variations in the input parameters^51^. The analysis involved analysing various feedstock: (1) glucose, (2) molasses, (3) glycerol and (4) sucrose for the fermentation process. A second sensitivity focused on regionality and plant location, which is linked with the energy mix of the country or region and supply chain issues. For the four fermentation cases, three different plant locations were assessed (Germany, Brazil and China). Uncertainty analysis was performed by Monte Carlo simulations. The uncertainty is estimated for the process(es) output impacts by using the Pedigree matrix approach^52^ and 1,000 iterations. Some background processes already included geometric standard deviations assigned to the respective flows (Ecoinvent data; Supplementary Data 3).

### TEA of bio-based process

TEA has gained attention in emerging biochemical and biotechnological process technologies as a decision-making tool^43,53^. This methodology is especially relevant to innovative processes or products that need assessment of commercialization opportunities^33^. The assessment presented here is a preliminary examination, with an outcome accuracy between ±30% and ±50% (ref. ^54^). The accuracy greatly depends on the quality and uncertainty of the input data, both experimental and from literature. Integrating engineering rules of thumb, experience and process simulation plays a key role in generating mass and energy balances (inventories), and further equipment sizing and costing^55^. TEA included estimating the fixed capital investment^56^ and operating cost, including utility, raw materials and labour cost, among others. Details about TEA calculations are documented in Supplementary File 1. This enabled a breakdown cost analysis and determining the cost drivers of the process^57^. The revenues for the base-case scenarios were estimated using an average product price of US$34.75 kg^−1^. The global production rate was calculated using market-size data^1,58^ and product prices reported by vendors (Supplementary File 1) as no direct global production quantification was found. The described calculation methodology allowed for estimating the payback period, the IRR, the return on investment (ROI) and the net present value, among other key parameters. The TEA included a sensitivity analysis to examine variations in production cost by function of feedstock, production capacity and titre^59^. Due to uncertainty on the product price, found ranging between US$8.5 kg^−1^ and US$61 kg^−1^, a sensitivity to evaluate profitability at different product prices was performed. The minimum price used in this study was recalculated from US$8.5 kg^−1^ to US$21.5 kg^−1^ until convergence to the financial targets, within the given market volume boundaries, was reached. In addition, uncertainty analysis was performed owing to the variability of the feedstock prices found for the three major raw material cost contributors. All related data can be found in Supplementary File 1.

### Statistics and reproducibility

The sample size was not statistically predetermined by any statistical method. The sample size was typically set at three (*n* = 3) for main experiments to achieve acceptable statistical power in a cost- and time-efficient manner. Measurements were taken from distinct samples. Statistical analysis was generally performed with Student’s *t*-test (one tailed; paired), using means and standard deviations. For all statistical tests, data distribution was assumed to be normal, but this was not formally tested. No data points were excluded from analysis. Microsoft applications (Excel and VBA) were used for the analysis of economic and environmental assessment results. Open-source python libraries (Plotly (5.16.1), Seaborn (0.11.2), Matplotlib (3.5.1), Pandas (1.4.2) and Numpy (1.21.5)) were used for data analysis and plotting.

### Reporting summary

Further information on research design is available in the Nature Portfolio Reporting Summary linked to this article.

### Supplementary information

Supplementary Information Supplementary Tables 1–33, Figs. 1–11 and detailed descriptions of LCA, TEA, and nucleotide and amino acid sequences.

Reporting Summary

Supplementary Data 1 A list of all biobricks, plasmids, strains and oligonucleotides used and/or generated in this work.

Supplementary Data 2 Fed-batch fermentation media composition, feed calculations and operational parameters.

Supplementary Data 3 Emission inventory collected from the Ecoinvent 3.8 database.

### Source data

Source Data Fig. 2 Statistical source data for metabolic engineering campaign.

Source Data Fig. 3 Statistical source data for fed-batch fermentations.

Source Data Fig. 5 Statistical source data for LCA.

Source Data Fig. 6 Statistical source data for TEA.

Source Data Extended Data Fig. 1 Statistical source data for UV–vis spectra and LC–MS.

Source Data Extended Data Fig. 2 Statistical source data for l-tyrosine supplementation cultivation.

Source Data Extended Data Fig. 3 Statistical source data for UV–vis spectra.

Source Data Extended Data Fig. 4 Statistical source data for UV–vis spectra.

Source Data Extended Data Fig. 5 Statistical source data for shake flask cultivations with different buffered media.

Source Data Extended Data Fig. 6 Statistical source data for shake flask cultivations with different buffered media.

Source Data Extended Data Fig. 7 Statistical source data for shake flask cultivations with different buffered media.

Source Data Extended Data Fig. 8 Statistical source data for metabolic engineering campaign with specific titres and cell dry weight.

Source Data Extended Data Fig. 9 Statistical source data for relevant fermentation parameters.

**Extended Data Fig. 10:**
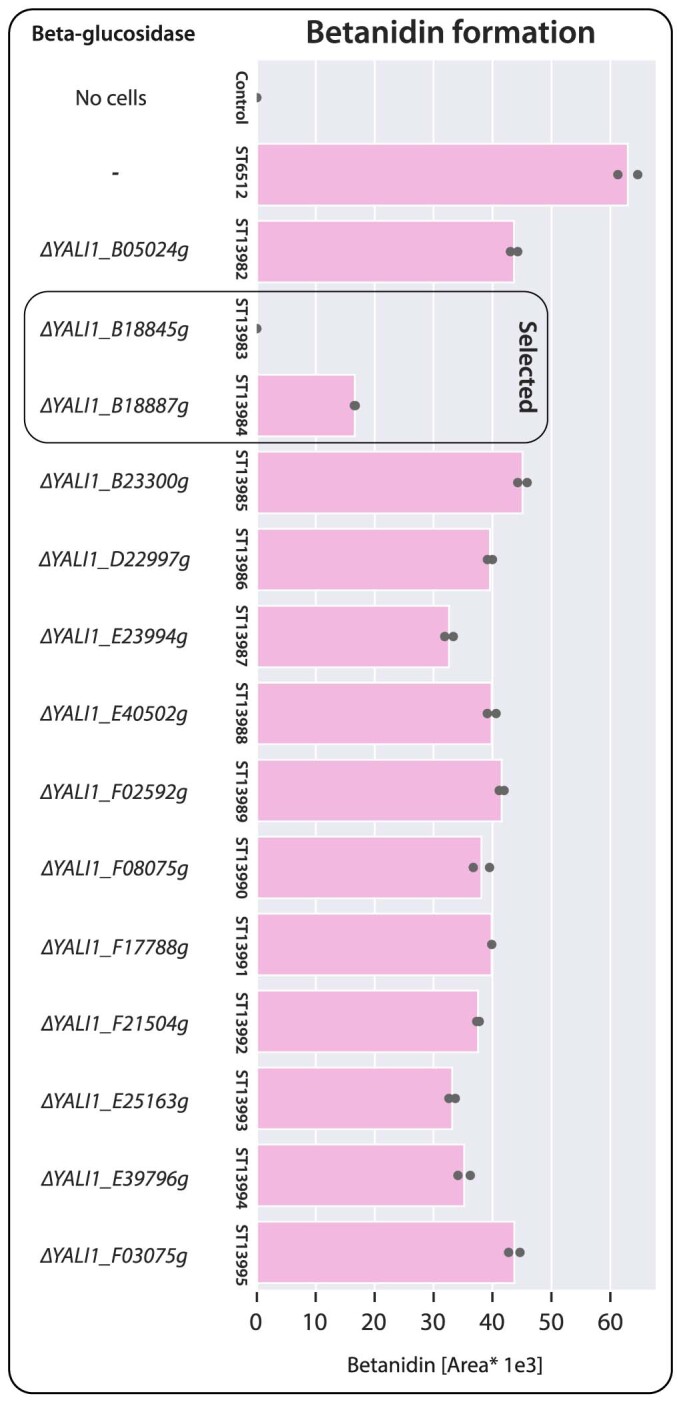
Betanin deglycosylation assay. The effect of beta-glucosidase disruption on betanidin formation was assessed by cultivating non-producing *Y. lipolytica* strains containing the individual beta-glucosidase disruptions in mineral media containing 100 mg/L betanin. Samples were collected after 24 h, and the betanidin formation assessed via HPLC. The control contained no inoculum, representing the spontaneous betanidin formation from betanin. The disruption of *YALI1_B18845g* and *YALI1_B18887g* greatly reduced betanidin formation, and thereby likely contribute to *Y. lipolytica*’s ability to degrade betanin by deglycosylation. Cultivations were carried out in biological duplicate (n = 2), with bars indicating averages. ‘area*’ indicates HPLC quantification by UV-vis spectra, without internal standards. Source data

Source Data Extended Data Fig. 10 Statistical source data for betanin deglycosylation assay.

## Data Availability

Data used, generated or analysed are available in the supplementary files. The nucleotide and amino acid sequences for all heterologous and native *Y. lipolytica* genes used for engineering can be found in Supplementary File 1. A list of all biobricks, plasmids, strains and oligonucleotides (primers) used and/or generated in this work can be found in Supplementary Data 1. The medium composition for the fermentations, as well as the operational parameters, can be found in Supplementary Data 2, along with the raw online data. The emissions inventory collected from the Ecoinvent 3.8 database used for the LCA can be found in Supplementary Data 3. All other data collected and used for the LCA and TEA can be found in Supplementary File 1. Biological materials used in this study are available upon reasonable request from the corresponding authors. Source data are provided with this paper.
