# Supplementary material for: Beet red food colourant can be produced more sustainably with engineered Yarrowia lipolytica
Source: Nat Microbiol. 2023 Nov 29;8(12):2290–303. doi: 10.1038/s41564-023-01517-5 (PMC10686825; doi:10.1038/s41564-023-01517-5)
Supplement: Supplementary file 1 — Supplementary Tables 1–33, Figs. 1–11 and detailed descriptions of LCA, TEA, and nucleotide and amino acid sequences. [file 41564_2023_1517_MOESM1_ESM.pdf]

nature microbiology

Article

https://doi.org/10.1038/s41564-023-01517-5

Beet red food colourant can be produced more sustainably with engineered *Yarrowia lipolytica*

In the format provided by the authors and unedited

Contents

1. Nucleotide and amino acid sequences for all native *Y. lipolytica* genes and heterologous genes used for engineering 4

Supplementary Table S1: Amino acid sequences and the corresponding *Yarrowia lipolytica* codon-optimized nucleotide sequences for the heterologous genes used in this study. 4

Supplementary Table S2: Amino acid sequences and the corresponding nucleotide sequences for the native *Yarrowia lipolytica* genes modulated in this study. 8

2. Overview of the sustainability assessment methodology applied to the betanin production 24

Supplementary Figure S1. Methodology applied for the sustainability assessment of betanin-based colorant 24

3. Market data and analysis of betanin production 24

Supplementary Table S3. Summary of market data, estimated from the aggregated food colorant market. 24

Supplementary Table S4. Wholesale price of E162, or "B2B" price. 25

Supplementary Table S5. Retail price of E162, or “B2C”, and the calculated B2B price assumed as one third of B2C. 25

Supplementary Table S6. B2B prices, market volume boundaries and upper and lower plant capacities assumed for the analysis. 26

Supplementary Figure S2. Global Import-export quantities in t/y (a) and Value in Million USD (b) from 2000 to 2019. 27

Supplementary Table S7. Top Exporter and Importer countries in terms of value in year 2018 27

4. Techno-economic assessment supporting data 28

Supplementary Figure S3. Block Flow Diagram of the traditional betanin production process. Minor streams, utilities and CIP, SIP waste is not reported. 28

Supplementary Figure S4. Block Flow Diagram of the fermentation based betanin production process. Minor streams, utilities and CIP (Cleaning-In-Place), SIP (Steam-in-Place) waste flows are not reported. 30

Supplementary Figure S5. Cleaning steps in the food industry 30

Supplementary Table S8. Equipment design parameters for the extraction-based process. 31

Supplementary Table S9. Process design parameters for the extraction-based process. 31

Supplementary Table S10. Equipment design parameters for the fermentation-based process, valid for all the different feedstock scenarios 33

Table S11. Process design parameters for the fermentation-based process, valid for all the different feedstock scenarios 34

Supplementary Table S12. Process reactions used as input in the stoichiometric reactor 35

Supplementary Table S13. Batch medium composition, valid for all the four feedstock scenarios 35

Supplementary Table S14. Batch and Fed-Batch medium composition of the main carbon source for each scenario 35

Supplementary Table S15. Prices of the raw materials used in the TEA 36

Supplementary Table S16. Cost of other utilities and waste treatment for Germany 36

Table S17. Financial parameters and their baseline values for Germany 37

Table S18. Process plant parameters and their baseline values 37

5. Techno-economic assessment results 38

Supplementary Table S19. Summary of results for the economic analysis – baseline scenarios 38

Supplementary Figure S6. Payback period (PBP) at three prices (high, mid, and low) by changes in fermentation titer for different feedstocks. 39

Supplementary Figure S7. Sensitivity of payback period to feedstock and titer variations 39

Supplementary Figure S9. Sensitivity of production cost by changes in production rate for different feedstocks. 40

Supplementary Figure S9. Uncertainty analysis of operating product cost, bars and error bars represent the mean of production cost (USD/kg) and standard deviation of each feedstock scenario, and the overlaying dots (n=3) represent the cost datapoints. 41

6. Life Cycle Assessment supporting data 41

Supplementary Figure S10. System boundaries of betanin-colorant production 42

Supplementary Table S20. Process inventory for extraction- and fermentation-based processes for 1 kg of product (base cases) 42

Supplementary Table S21. Pedigree matrix criteria for assigning geometric means. 43

Supplementary Table S22. Process assembly inventory for betanin production via extraction 44

Supplementary Table S23. Process assembly inventory for betanin production via glucose fermentation 44

Supplementary Table S24. Process assembly inventory for betanin production via glycerol fermentation 44

Supplementary Table S25. Process assembly inventory for betanin production via molasses fermentation 45

Supplementary Table S26. Process assembly inventory for betanin production via sucrose fermentation 45

Supplementary Table S27. Sources of background system data 45

Supplementary Table S28. normalized inventory of sensitivity scenarios for glucose process 46

Supplementary Table S29. normalized inventory to 1 kg of product for glycerol sensitivity scenarios 47

Supplementary Table S30. normalized inventory of sensitivity scenarios for molasses process..... 48

Supplementary Table S31. normalized inventory of sensitivity scenarios for sucrose process 49
5.2. Life Cycle Impact Assessment results 50
Supplementary Table S32. Midpoint results of fermentation-based betanin production 50
Supplementary Figure S11. Normalized midpoint impacts and uncertainty of fermentation-based scenarios varying feedstock (glucose, molasses, glycerol, and sucrose) compared with the extraction-based process. Data given in bars and error bars represent the mean and standard deviation of evaluated scenarios, dots represent the normalized impact points with n=1,000 simulations generated in the built-in Monte Carlo algorithm in SimaPro software. 51
Supplementary Table S33. Midpoint results of fermentation-based betanin production 52
Supplementary Figure S11. Heatmaps of endpoint impacts by country sensitivity for different feedstocks. 52

1. Nucleotide and amino acid sequences for all native *Y. lipolytica* genes and heterologous genes used for engineering

Supplementary Table S1: Amino acid sequences and the corresponding *Yarrowia lipolytica* codon-optimized nucleotide sequences for the <u>heterologous</u> genes used in this study.

Gene	Sequence	Source
BvTYH (AET43289.1)	MDHATLAMILAI***L***FISFHFIKLLFSQQTTKLLPPGPKPLPIIGNILEVGKKPHRSFANLAKIHGPLISLRLGSVT<br>TIVVSSADVAKEMFLKKDHPLSNRTIPNSVTAGDHHKLTMSWLPVSPKWRNFRKITAVHLLSPQRLDAC<br>QTFRHAKVQQLYEYVQECAQKGQAVDIGKAAFTTSLNLLSKLFFSVELAHHKSHTSQEFKELIWNIMEDI<br>GKPNYADYFPILGCVDPSGIRRRLACSFDKLIAVFQGIICERLAPDSSTTTTTTTDDVLDVLLQLFKQNELT<br>MGEINHLLVDIFDAGTDTTSSTFEWVMTELIRNPEMMEKAQEEIKQVLGKDKQIQESDIINLPYLQAIIKE<br>TLRLHPPTVFLLPRKADTDVELYGYIVPKDAQILVNLWAIGRDPNAWQNADIFSPERFIGCEIDVKGRDF<br>GLLPFGAGRRICPGMNLAIRMLTLMLATLLQFFNWKLEGDISPKDLDMDEKFGIALQKTKPLKLIPIPRY*	[1]
BvTYH_Yl	ATGGACCACGCTACCCTGGCCATGATCCTGGCTATCCTGTTCATCTCGTTCCACTTCATCAAGCTGCT<br>GTTCTCTCAGCAGACCACTAAGCTGCTGCCTCCAGGACCTAAGCCTCTGCCTATCATCGGCAACATC<br>CTCGAGGTGGGCAAGAAGCCCCACCGATCTTTCGCCAACCTGGCCAAGATTCACGGACCCCTGATC<br>TCCCTGCGACTGGGCTCTGTGACTACCATCGTGGTGTCCTCTGCCGACGTGGCCAAGGAAATGTTCC<br>TGAAGAAGGACCATCCTCTGTCTAACCGAACCATTCCTAACTCTGTGACCGCTGGCGACCACCACAA<br>GCTGACCATGTCTTGGCTGCCCGTGTCTCCCAAGTGGCGAAACTTCCGAAAGATCACCGCCGTGCAT<br>CTGCTGTCTCCCCAGCGACTGGACGCCTGCCAGACCTTCCGACACGCCAAGGTGCAGCAGCTGTAC<br>GAGTACGTGCAAGAGTGCGCCCAAAAGGGCCAAGCCGTGGACATCGGCAAGGCCGCCTTCACCAC<br>CTCGCTGAACCTGCTGTCTAAGCTGTTCTTCTCGGTCGAGCTGGCTCACCACAAGTCTCACACCTCTC<br>AAGAGTTCAAGGAACTGATCTGGAACATCATGGAAGATATCGGCAAGCCCAACTACGCCGACTACT<br>TCCCCATCCTGGGCTGCGTGGACCCCTCTGGCATCCGACGACGACTGGCCTGCTCTTTCGACAAGCT<br>GATCGCCGTGTTCCAGGGCATCATCTGCGAGCGACTGGCTCCCGACTCTTCTACCACCACTACTACC<br>ACTACCGACGACGTGCTGGACGTCCTGCTGCAGCTGTTCAAGCAGAACGAGCTGACTATGGGCGA<br>GATCAACCACCTCCTGGTGGACATCTTCGACGCCGGCACCGACACCACCTCTTCCACCTTCGAGTGG<br>GTGATGACCGAGCTGATTCGAAACCCCGAGATGATGGAAAAGGCCCAAGAGGAAATCAAGCAGGT<br>CCTCGGCAAGGACAAGCAGATCCAAGAGTCTGACATCATCAACCTGCCTTACCTGCAGGCCATCATC<br>AAGGAAACCCTCCGACTGCACCCTCCTACCGTGTTTCTGCTGCCCCGAAAGGCCGACACCGACGTC<br>GAGCTGTACGGCTACATCGTGCCCAAGGACGCCCAGATCCTGGTGAACCTGTGGGCCATCGGACG<br>AGATCCCAACGCCTGGCAGAACGCCGACATCTTCTCGCCCGAGCGATTCATCGGCTGCGAGATCGA<br>CGTGAAGGGCCGAGACTTCGGACTGCTGCCCTTCGGAGCCGGCCGACGAATCTGCCCCGGCATGA<br>ACCTCGCCATCCGAATGCTGACCCTGATGCTGGCCACTCTGCTGCAGTTCTTCAACTGGAAGCTCGA<br>GGGCGACATCTCTCCCAAGGACCTGGACATGGACGAGAAGTTCGGAATCGCCCTGCAAAAGACTA<br>AGCCCCTGAAGCTGATCCCCATTCCTCGATACTGA	
EvTYH (AKI33945.1)	MDHTTLAMILSAIFLLYNLAKAIFSHSNTKLPPGPKPVPIFGNIFELGEKPHRSFANLAKIHGPLITLKLGSV<br>TTIVVSSAEVAKEMFLKNDLPLANRNVPNSVTAGDHHKLTMSWLPVSPKWKTFRKITAVHLLSPQRLDS<br>CQALRHTKVKQLHQYVQECAKRGEPVDIGKAAFTTSLNLLSNLFFSVELANHTSSSSQEFKELIWEIMEDI<br>GKPNYADYFPILKCVDPWGIRRRLASNFDKLIEVFQGFIRKRLSTGSFSAITPNDVLDVLLNLLKEKELNM<br>GEINHLLVDIFDAGTDTTSSTFEWAMAELVRNQEMMKKAQDEIEQVLGKDAIIQESDIPKMPYLQAIIKE<br>TLRLHPPTVFLLPRKATSNVELYGYVVPKNAQILVNLWAIGRDPKVWDNPNMFSPERFLNSEIDVKGRD<br>FGLLPFGAGRRICPGMNLAYRMLTLMLATLLQSFDWKLGDGVNPKDLDMEEKFGIALQKTKPLQVIPVL<br>KY*	
EvTYH_Yl	AGTGACCACACCACTCTGGCCATGATCCTGTCTGCCATCTTCCTGCTGTACAACCTGGCCAAGGCCA<br>TCTTCTCTCACTCTAACACCAAGCTGCCTCCAGGACCTAAGCCTGTGCCTATCTTCGGCAACATCTTC<br>GAGCTGGGCGAGAAGCCCCACCGATCTTTCGCCAACCTGGCTAAGATTCACGGACCCCTGATCACC<br>CTGAAGCTGGGCTCTGTGACTACCATCGTGGTGTCCTCTGCCGAGGTGGCCAAGGAAATGTTCCTG<br>AAGAACGACCTGCCTCTGGCCAACCGAAACGTGCCCAACTCTGTGACCGCTGGCGACCACCACAAG<br>CTGACCATGTCTTGGCTGCCCGTGTCTCCCAAGTGGAAGACCTTCCGAAAGATCACCGCCGTGCATC	

	TGCTGTCTCCCCAGCGACTGGACTCTTGTCAGGCCCTGCGACACACCAAGGTGAAGCAGCTGCACC<br>AGTACGTGCAAGAGTGCGCCAAGCGAGGCGAGCCCGTGGACATCGGCAAGGCCGCCTTCACCACC<br>TCGCTGAACCTGCTGTCTAACCTGTTCTTCTCGGTCGAGCTGGCTAACCACACCTCTTCGTCCTCTCA<br>AGAGTTCAAGGAACTGATCTGGGAGATCATGGAAGATATCGGCAAGCCCAACTACGCCGACTACTT<br>CCCCATCCTGAAGTGCGTGGACCCCTGGGGCATCCGACGACGACTGGCCTCTAACTTCGACAAGCT<br>GATCGAGGTGTTCCAGGGCTTCATCCGAAAGCGACTGTCTACCGGCTCTTTCTCTGCCATCACTCCC<br>AACGACGTGCTGGACGTGCTCCTGAACCTCCTGAAGGAAAAGGAACTCAACATGGGCGAGATCAA<br>CCACCTCCTGGTGGACATTTTCGACGCCGGCACCGACACCACCTCTTCTACCTTCGAGTGGGCCATG<br>GCTGAGCTGGTGCGAAACCAAGAGATGATGAAGAAGGCCCAGGACGAGATCGAGCAGGTCCTCG<br>GAAAGGACGCCATCATCCAAGAGTCTGACATCCCCAAGATGCCCTACCTGCAGGCCATCATCAAGG<br>AAACCCTGCGACTGCACCCTCCTACCGTGTTTCTGCTGCCCCGAAAGGCCACCTCTAACGTCGAGCT<br>GTACGGCTACGTGGTGCCCAAGAACGCCCAGATCCTGGTGAACCTGTGGGCCATCGGTCGAGATCC<br>CAAGGTGTGGGACAACCCCAACATGTTCTCGCCCGAGCGATTCCTGAACTCCGAGATCGACGTGAA<br>GGGCCGAGACTTCGGACTGCTGCCCTTCGGAGCCGGCCGACGAATCTGCCCCGGCATGAACCTGG<br>CCTACCGAATGCTGACCCTGATGCTGGCTACCCTGCTGCAGTCTTTCGACTGGAAGCTCGGCGACG<br>GCGTGAACCCCAAGGACCTGGACATGGAAGAGAAGTTCGGCATTGCCCTGCAAAAGACCAAGCCT<br>CTGCAGGTCATCCCCGTGCTGAAGTACTAA	
AnTYH<br>*(AKI33952.1)*	MDQTTLAMLLSALYLLYNLYKVIFTQSNSKLPPGPKPLPIFGNISELGAKPHRSFANLAKIHGPLITLKLGSV<br>TTIVVSSAKVAEEMFLKNDLPLANRNVPNSVTAGDHHKLTMSWLPVSPKWKTFRKITAVHLLSPQRLDA<br>CQALRHAKVKQLYEYVYDCAKKGEAVDIGKAAFTTSLNLLSNLFFSVELAQHTSTSSQHFKQLIWDIMED<br>IGKPNYADYFPALKCVDPWGIRRRLAANFERLIDVFQDFIRPRLSMNPSSVTSASDVLDVLLNLYKEKELN<br>MGEVNHLLVDIFDAGTDTTSSTFEWAMAELVRHPETMKKAQDEIEQVLGKDATIQEADIPKMPYLQAII<br>KETLRLHPPTVFLLPRKAATNVELYGYVVPKDAQILVNLWAIGRDPLVWDQPNVFSPERFLNSDVDVKG<br>RDFGLLPFGAGRRICPGMNLAYRMLTLMLATLLQSFEWKVENGEKAEDLDMDEKFGIALQKTKPLQIIP<br>VLKYC*	2
AnTYH_Yl	ATGGACCAGACCACTCTGGCCATGCTGCTGTCTGCCCTGTACCTGCTGTACAACCTGTACAAGGTGA<br>TCTTCACCCAGTCTAACTCTAAGCTGCCTCCTGGACCTAAGCCTCTGCCTATCTTCGGCAACATCTCT<br>GAGCTGGGCGCTAAGCCCCACCGATCTTTCGCCAACCTGGCCAAGATTCACGGACCCCTGATCACC<br>CTGAAGCTGGGCTCTGTGACTACCATCGTGGTGTCCTCTGCCAAGGTGGCCGAGGAAATGTTCCTG<br>AAGAACGACCTGCCTCTGGCCAACCGAAACGTGCCCAACTCTGTGACCGCTGGCGACCACCACAAG<br>CTGACCATGTCTTGGCTGCCCGTGTCTCCCAAGTGGAAGACCTTCCGAAAGATCACCGCCGTGCATC<br>TGCTGTCTCCCCAGCGACTGGACGCCTGTCAGGCCCTGCGACACGCCAAGGTGAAGCAGCTGTACG<br>AGTACGTGTACGACTGCGCCAAGAAGGGCGAAGCCGTCGACATCGGCAAGGCCGCCTTCACCACC<br>TCGCTGAACCTGCTCTCGAACCTGTTCTTCTCGGTCGAGCTGGCCCAGCACACCTCTACCTCTTCGCA<br>GCACTTCAAGCAGCTGATCTGGGACATCATGGAAGATATCGGCAAGCCCAACTACGCCGACTACTT<br>CCCCGCTCTGAAGTGCGTGGACCCCTGGGGCATCCGACGACGACTGGCCGCCAACTTCGAGCGACT<br>GATCGACGTGTTCCAGGACTTCATTCGACCCCGACTGTCTATGAACCCCTCTTCTGTGACCTCTGCCT<br>CTGACGTGCTGGACGTGCTCCTGAACCTCTACAAGGAAAAGGAACTGAACATGGGCGAAGTGAAC<br>CACCTCCTGGTGGACATCTTCGACGCCGGCACCGACACCACCTCTTCTACCTTCGAGTGGGCCATGG<br>CTGAGCTGGTGCGACACCCCGAGACTATGAAGAAGGCCCAGGACGAGATCGAGCAGGTCCTCGGA<br>AAGGACGCCACCATCCAAGAGGCCGACATTCCCAAGATGCCCTACCTGCAGGCCATCATCAAGGAA<br>ACCCTGCGACTGCACCCTCCTACCGTGTTTCTGCTGCCCCGAAAGGCCGCTACCAACGTCGAGCTGT<br>ACGGCTACGTCGTGCCCAAGGACGCCCAGATCCTGGTGAACCTGTGGGCTATCGGTCGAGATCCCC<br>TGGTGTGGGACCAGCCTAACGTGTTCTCGCCCGAGCGATTCCTGAACTCCGACGTGGACGTGAAGG<br>GCCGAGACTTCGGACTGCTGCCCTTCGGAGCCGGCCGACGAATCTGCCCCGGCATGAACCTGGCCT<br>ACCGAATGCTGACCCTGATGCTGGCTACCCTGCTGCAGTCTTTCGAGTGGAAGGTCGAGAACGGCG<br>AGAAGGCTGAGGACCTGGACATGGACGAGAAGTTCGGCATTGCCCTGCAAAAGACTAAGCCCCTG<br>CAGATCATCCCCGTGCTGAAGTACTGCTAA	
BgDOD2<br>*(*BAG80687.1*)*	MGGEKKMKGTYYIAHGDPIMYINKSIKLRHFLEEWKENVVMEKPICILVISAHWDTDVPTVNLVEHCDT<br>IHDFDDYPDPLYQIKYPAPGAPKLAMKVQELLKGGGFKCEVDTKRGLDHAAWFPLMLMYPEADIPICEL<br>SVQTNKDGTHHYNLGKALSPLLNDDVLIIGSGGAVHPSDDTPHCPNGVAPWALQFDNWLEDALLSGR<br>YEDVKEFKKMAPNWEISHPGQEHLYPLHVALGAAGNNVKTELIHQTWAANGVFGYSSYKFTST*	2

BgDOD2_Yl	ATGGGCGGCGAGAAGAAGATGAAGGGCACCTACTACATTGCCCACGGCGACCCCATCATGTACAT<br>CAACAAGTCTATCAAGCTGCGACACTTCCTGGAAGAGTGGAAGGAAAACGTGGTGATGGAAAAGC<br>CCATCTGCATCCTGGTGATCTCTGCCCACTGGGACACCGACGTGCCCACCGTGAACCTGGTCGAGC<br>ACTGCGACACCATCCACGACTTCGACGACTACCCCGATCCTCTGTACCAGATCAAGTACCCCGCTCC<br>TGGCGCTCCCAAGCTGGCCATGAAGGTGCAAGAGCTGCTGAAGGGCGGAGGCTTCAAGTGCGAG<br>GTGGACACCAAGCGAGGCCTGGACCACGCCGCATGGTTCCCTCTGATGCTGATGTACCCCGAGGCC<br>GACATTCCCATCTGCGAGCTGTCTGTGCAGACCAACAAGGACGGCACCCACCACTACAACCTCGGC<br>AAGGCTCTGTCTCCCCTGCTGAACGACGACGTGCTGATCATCGGCTCTGGCGGCGCTGTGCACCCCT<br>CTGACGACACCCCTCACTGCCCCAACGGCGTGGCTCCCTGGGCTCTGCAGTTCGACAACTGGCTCG<br>AGGACGCCCTGCTGTCTGGCCGATACGAGGACGTGAAGGAATTCAAGAAGATGGCTCCCAACTGG<br>GAGATCTCTCACCCCGGCCAAGAGCATCTGTACCCTCTGCACGTGGCCCTGGGAGCCGCCGGAAAC<br>AACGTCAAGACCGAGCTGATCCACCAGACCTGGGCCGCTAACGGCGTGTTCGGCTACTCTTCTTAC<br>AAGTTCACCTCTACCTAA
MjDOD<br>*(B6F0W8.1)*	MKGTYYINHGDPLMYLKKHIKLRQFLEGWQENVVIEKPKSILIISAHWDTNVPTVNFVEHCDTIHDFDDY [3]<br>PDPLYQIQYRAPGAPNLAKKVEELLKESGMECEIDTKRGLDHAAWFPLMFMYPEANIPICELSVQPSKD<br>GIHHYNVGKALSPLLQQGVLIIGSGGTVHPSDDTPHCPNGVAPWAIEFDNWLEDALLSGRYEDVNNFK<br>KLAPNWEISHPGQEHLYPLHVALGAAGKNPKTQLIHRSWAANGVFGYSTYNFTPTTQKTDV*
MjDOD_Yl	ATGAAGGGCACCTACTACATCAACCACGGCGACCCTCTGATGTACCTGAAGAAGCACATCAAGCTG<br>CGACAGTTTCTGGAAGGCTGGCAAGAGAACGTGGTGATCGAGAAGCCCAAGTCTATCCTGATCATC<br>TCTGCCCACTGGGACACCAACGTGCCCACCGTGAACTTCGTCGAGCACTGCGACACCATCCACGACT<br>TCGACGACTACCCCGATCCTCTGTACCAGATCCAGTACAGAGCCCCTGGCGCTCCCAACCTGGCCAA<br>GAAGGTCGAGGAACTGCTGAAGGAATCTGGCATGGAATGCGAGATCGACACCAAGCGAGGCCTG<br>GACCACGCCGCATGGTTCCCTCTCATGTTCATGTACCCCGAGGCCAACATTCCCATCTGCGAGCTGT<br>CTGTGCAGCCCTCTAAGGACGGCATCCACCACTACAACGTCGGCAAGGCTCTGTCTCCCCTGCTCCA<br>GCAGGGCGTGCTGATCATCGGCTCTGGCGGCACCGTGCATCCCTCTGACGACACCCCTCACTGCCC<br>CAACGGCGTGGCTCCCTGGGCCATCGAGTTCGACAACTGGCTCGAGGACGCTCTGCTGTCTGGCCG<br>ATACGAGGACGTGAACAACTTCAAGAAGCTGGCTCCCAACTGGGAGATCTCTCACCCCGGCCAAGA<br>GCATCTGTACCCTCTGCACGTGGCCCTGGGCGCTGCCGGCAAGAACCCCAAGACTCAGCTGATCCA<br>CCGATCTTGGGCCGCTAACGGCGTGTTCGGCTACTCTACCTACAACTTCACCCCTACCACTCAAAAG<br>ACCGACGTGTAA
DbB5GT<br>*UGT73A5*<br>*(CAB56231.1)*	MGTHSTAPDLHVVFFPFLAHGHMIPSLDIAKLFAARGVKTTIITTPLNASMFTKAIEKTRKNTETQMEIEV [4]<br>FSFPSEEAGLPLGCENLEQAMAIGANNEFFNAANLLKEQLENFLVKTRPNCLVADMFFTWAADSTAKF<br>NIPTLVFHGFSFFAQCAKEVMWRYKPYKAVSSDTEVFSLPFLPHEVKMTRLQVPESMRKGEETHFTKRT<br>ERIRELERKSYGVIVNSFYELEPDYADFLRKELGRRAWHIGPVSLCNRSIEDKAQRGRQTSIDEDECLKWL<br>NSKKPDSVIYICFGSTGHLIAPQLHEIATALEASGQDFIWAVRGDHGQGNSEEWLPPGYEHRLQGKGLII<br>RGWAPQVLILEHEATGGFLTHCGWNSALEGISAGVPMVTWPTFAEQFHNEQLLTQILKVGVAVGSKK<br>WTLKPSIEDVIKAEDIEKAVREVMVGEEGEERRRRAKKLKEMAWRAIEEGGSSYSDLSALIEELKGYHTSE<br>KE*
DbB5GT_Yl	ATGGGCACCCACTCTACTGCTCCCGACCTGCACGTGGTGTTCTTCCCTTTCCTGGCTCACGGCCACAT<br>GATCCCTTCTCTGGACATTGCCAAGCTGTTCGCCGCTCGAGGCGTCAAGACCACCATCATCACCACT<br>CCTCTGAACGCCTCTATGTTCACCAAGGCCATCGAAAAGACCCGAAAGAACACCGAGACTCAGATG<br>GAAATCGAGGTGTTCTCGTTCCCCTCGGAGGAAGCCGGACTGCCCCTGGGCTGCGAGAACCTCGA<br>GCAGGCCATGGCTATCGGCGCCAACAACGAGTTCTTCAACGCCGCCAACCTGCTGAAGGAACAGCT<br>CGAGAACTTCCTGGTCAAGACCCGACCTAACTGCCTGGTGGCCGACATGTTCTTCACCTGGGCCGCT<br>GACTCTACCGCCAAGTTCAACATCCCCACTCTGGTGTTCCACGGCTTCTCGTTCTTCGCCCAGTGCGC<br>CAAGGAAGTGATGTGGCGATACAAGCCCTACAAGGCCGTGTCCTCTGACACCGAGGTGTTTTCTCT<br>GCCCTTCCTGCCTCACGAGGTGAAGATGACCCGACTGCAGGTCCCCGAGTCTATGCGAAAGGGCGA<br>AGAGACTCACTTCACCAAGCGAACCGAGCGAATCCGAGAGCTGGAACGAAAGTCTTACGGCGTGA<br>TCGTGAACTCTTTCTACGAGCTTGAGCCCGACTACGCCGACTTCCTGCGAAAGGAACTGGGCCGAC<br>GAGCCTGGCACATCGGCCCCGTGTCTCTGTGCAACCGATCTATCGAGGACAAGGCCCAGCGAGGCC

	GACAGACCTCTATCGACGAGGACGAGTGCCTGAAGTGGCTGAACTCTAAGAAGCCCGACTCTGTCA<br>TCTACATCTGCTTCGGCTCTACCGGCCACCTGATCGCTCCCCAGCTGCACGAGATCGCCACCGCTCTC<br>GAGGCCTCTGGCCAGGACTTCATTTGGGCCGTGCGAGGCGACCACGGCCAGGGCAACTCTGAGGA<br>ATGGCTGCCTCCTGGCTACGAGCACCGACTGCAAGGCAAGGGCCTGATCATCCGAGGCTGGGCTCC<br>CCAGGTGCTGATCCTCGAGCACGAGGCCACCGGCGGCTTTCTGACCCACTGCGGCTGGAACTCTGC<br>CCTGGAAGGCATCTCTGCCGGCGTGCCCATGGTGACCTGGCCTACCTTCGCCGAGCAGTTCCACAA<br>CGAACAGCTGCTGACCCAGATCCTGAAGGTGGGCGTCGCCGTGGGATCTAAGAAGTGGACCCTGA<br>AGCCTTCCATCGAGGACGTGATCAAGGCCGAGGACATCGAGAAGGCCGTCCGAGAGGTGATGGTC<br>GGCGAGGAAGGTGAGGAAAGACGACGACGAGCCAAGAAGCTCAAGGAAATGGCCTGGCGAGCC<br>ATTGAGGAAGGCGGATCTTCTTACTCTGACCTGTCTGCCCTGATCGAGGAACTGAAGGGCTACCAC<br>ACCTCTGAGAAGGAATAG
BvSGT2<br>*UGT73A36*<br>*(KMT01176.1)*	MDDKSQQLHIVLFPFMAHGHMIPTLDIARLFAARGVKTTLITTPRNAPTFLTAIEKGNKSGAPTINVEVF 2<br>NFQAQSFGLPEGCENLEQALGPGIRDRFFKAAAMLRDQLEHFLEKTRPNCLVADMFFPWATDSAAKF<br>NIPRLVFHGHCLFALCALEIIRLHEPYNNASSDEEPFLLPHLPHEIELTRLQFSEELWKNGGDSDYKERSKAI<br>KESELKCYGVLVNSFYELEPDYAEYFRKDLGRRAWNIGPVSLYNRSNEEKAQRGKQASIDEHECLKWLNS<br>KKPNSVIYICFGSTMHMIPSQLNEIAMGLEASGKDFIWVVRNEDDLGEFEHRMEGKGLIIRGWAPQVLI<br>LEHEVIGAFVTHCGWNSTIEGIAAGVPMVTWPVFAEQFLNEKLITRVLRIGIPVGAKKWDCKPCEEYVV<br>KKNDIEKALREVMEGNEAEERRTRAKEYKEMAWKALQEGGSSYSDLSALIDELRGLST*
BvSGT2_Yl	ATGGACGACAAGTCTCAGCAGCTGCACATCGTGCTGTTCCCTTTCATGGCCCACGGCCACATGATCC<br>CCACTCTGGACATTGCCCGACTGTTCGCCGCTCGAGGCGTCAAGACCACTCTGATCACCACTCCTCG<br>AAACGCTCCCACCTTCCTGACCGCCATCGAGAAGGGCAACAAGTCTGGCGCCCCTACCATCAACGTC<br>GAGGTGTTCAACTTCCAGGCTCAGTCTTTCGGACTGCCCGAGGGCTGCGAGAACCTCGAGCAGGCT<br>CTCGGACCCGGCATCCGAGATCGATTCTTCAAGGCCGCTGCCATGCTGCGAGATCAGCTCGAGCAC<br>TTCCTGGAAAAGACCCGACCTAACTGCCTGGTGGCCGACATGTTCTTCCCTTGGGCCACCGACTCTG<br>CCGCCAAGTTCAACATCCCTCGACTGGTGTTCCACGGACACTGCCTGTTCGCCCTGTGCGCCCTCGA<br>GATCATCCGACTGCACGAGCCCTACAACAACGCCTCTTCTGACGAGGAACCCTTCCTGCTGCCTCAT<br>CTGCCCCACGAGATCGAGCTGACCCGACTGCAGTTCTCTGAGGAACTGTGGAAGAACGGCGGCGA<br>CTCTGACTACAAGGAACGATCTAAGGCCATCAAGGAATCTGAGCTGAAGTGCTACGGCGTGCTGGT<br>GAACTCTTTCTACGAGCTGGAACCCGACTACGCCGAGTACTTCCGAAAGGACCTGGGCCGACGAGC<br>TTGGAACATCGGCCCCGTGTCTCTGTACAACCGATCTAACGAGGAAAAGGCCCAGCGAGGCAAGC<br>AGGCCTCTATCGACGAGCACGAGTGCCTGAAGTGGCTGAACTCTAAGAAGCCCAACTCTGTCATCT<br>ACATCTGCTTCGGCTCTACCATGCACATGATTCCCTCGCAGCTGAACGAGATCGCCATGGGACTCGA<br>GGCCTCTGGCAAGGACTTCATCTGGGTCGTGCGAAACGAGGACGACCTGGGCGAGTTCGAGCACC<br>GAATGGAAGGCAAGGGCCTGATCATTCGAGGCTGGGCTCCCCAGGTGCTGATCCTGGAACACGAG<br>GTGATCGGCGCCTTCGTGACCCACTGCGGCTGGAACTCTACCATCGAGGGCATTGCCGCTGGCGTG<br>CCCATGGTGACCTGGCCTGTGTTCGCCGAGCAGTTTCTCAACGAGAAGCTGATCACCCGAGTCCTG<br>CGAATCGGCATCCCCGTGGGCGCCAAGAAGTGGGACTGCAAGCCCTGCGAGGAATACGTGGTGAA<br>GAAGAACGACATTGAGAAGGCTCTGCGAGAGGTGATGGAAGGTAACGAGGCCGAGGAACGACGA<br>ACCCGAGCCAAGGAATACAAGGAAATGGCCTGGAAGGCCCTGCAAGAAGGCGGATCTTCTTACTC<br>TGACCTGTCTGCCCTGATTGACGAACTGCGAGGCCTGTCTACCTAA

102

Supplementary Table S2: Amino acid sequences and the corresponding nucleotide sequences for the <u>native</u> *Yarrowia lipolytica* genes modulated in this study.

Gene	Sequence	Source
ARO4^{K221L}	MSRSSSPNASSAEDVRILGYDPLLAPALLQTEVASTKNARETVSKGRKDSIDVITGKSDKLLCIVGPC<br>SLHDPKAAMEYAQRLKELSDKLSGELVIVMRAYLEKPRTTVGWKGLINDPDMDESFNINKGLRLSR<br>KVFCDLTDLGLPIASEMLDTISPQFLADLLSLGAIGARTTESQLHRELASGLSFPVGFKNGTDGTLGV<br>AVDAVQAASHPHHFMGVT***L***QGVAAITTTKGNENCFIILRGGKKGTNYDAESVAECKKATESMLM<br>VDCSHGNSNKDYRNQPKVSKAVAEQVAAGEKKIIGVMIESNIHEGNQKVPKEGPSALKYGVSITD<br>ACVSWETTVDMLTELANAVKERRNKN*	5
YALI1_C09308g	AGTTCCCGTTCCTCCTCTCCCAACGCCTCCTCTGCTGAGGACGTGCGAATTCTGGGCTACGACC<br>CCCTCCTCGCTCCCGCTCTTCTCCAGACTGAGGTTGCCTCCACCAAAAACGCCCGAGAGACCGT<br>CTCCAAGGGCCGAAAGGACTCCATTGATGTCATCACCGGCAAGTCCGACAAGTTGCTGTGCAT<br>TGTCGGTCCCTGCTCCCTCCACGACCCCAAGGCCGCCATGGAGTACGCCCAGCGACTCAAGGA<br>GCTGTCTGACAAGCTGTCTGGTGAGCTCGTCATCGTTATGCGAGCCTACCTCGAGAAGCCCCG<br>AACCACCGTTGGCTGGAAGGGTCTGATCAACGACCCCGACATGGACGAGTCTTTCAACATCAA<br>CAAGGGTCTGCGACTCTCCCGAAAGGTCTTCTGCGACCTTACCGATCTGGGTCTGCCCATTGCC<br>TCCGAGATGCTCGATACCATTTCTCCCCAGTTCCTGGCCGACCTGCTCTCCCTGGGTGCCATTG<br>GTGCTCGAACCACCGAGTCCCAGCTGCACCGAGAGCTCGCCTCCGGTCTGTCTTTCCCCGTTG<br>GTTTCAAGAACGGAACCGACGGTACTCTGGGTGTTGCCGTTGATGCTGTCCAGGCCGCCTCTC<br>ACCCTCACCACTTCATGGGTGTCACC***CT***GCAGGGTGTTGCCGCCATCACCACCACCAAGGGTA<br>ACGAGAACTGCTTCATCATTCTGCGAGGAGGTAAGAAGGGCACCAACTACGACGCCGAGTCC<br>GTCGCCGAGTGCAAGAAGGCCACCGAGTCCATGCTCATGGTTGACTGCTCTCACGGCAACTCC<br>AACAAGGACTACCGAAACCAGCCCAAGGTTTCCAAGGCCGTTGCTGAGCAGGTTGCTGCTGG<br>CGAGAAGAAGATCATCGGTGTCATGATCGAGAGTAATATCCACGAGGGCAACCAGAAGGTCC<br>CCAAGGAGGGCCCCTCTGCCCTTAAATACGGTGTCTCCATCACCGACGCCTGTGTCTCTTGGG<br>AGACCACCGTGGACATGCTCACCGAGCTGGCCAACGCCGTCAAGGAGCGACGAAACAAGAAC<br>TAA	
ARO7^{G139S}	MDFTKADTVLDLANIRDSLVRMEDTIVFNLIERAQFCRSEFVYKAGNSDIPGFKGSYLDWFLQESE<br>KVHAKLRRYAAPDEQAFFPDDLPEAILPPIDYAPILAPYSKEVSVNDEIKKIYTDDIVPLVCAGTGDQ<br>PENY***S***SVMVCDIETLQALSRRIHFGKFVAESKFLSETERFTELIKNKDIAGIEAAITNSKVEETILARLG<br>EKALAYGTDPTLRWSQRTQGKVDSEVVKRIYKEWVIPLTKKVEVDYLLRRLE*	5
YALI1_E20751g	ATGGACTTCACTAAAGCCGACACCGTTCTGGATCTCGCCAACATCCGAGACTCGCTGGTCCGA<br>ATGGAGGACACTATTGTCTTCAATCTGATTGAGCGGGCTCAGTTCTGCCGTTCCGAGTTTGTGT<br>ACAAGGCCGGCAACTCGGACATTCCCGGCTTCAAGGGCTCTTACCTCGACTGGTTTCTGCAGG<br>AGTCGGAAAAGGTGCACGCCAAACTGCGTCGGTACGCTGCCCCGGACGAGCAGGCCTTCTTC<br>CCCGACGATCTACCCGAGGCCATTCTGCCCCCCATCGATTATGCGCCAATTCTGGCACCCTACA<br>GCAAGGAGGTGAGCGTCAACGACGAGATTAAAAAGATTTACACCGACGACATTGTGCCCCTG<br>GTGTGTGCTGGCACTGGAGATCAGCCCGAGAACTAT***TCC***TCGGTCATGGTGTGCGACATCGA<br>GACGCTGCAGGCGCTGTCGCGACGAATCCACTTTGGCAAGTTTGTGGCCGAGTCCAAGTTTCT<br>GAGTGAAACCGAGCGATTCACCGAGCTCATCAAGAACAAGGACATTGCTGGTATTGAGGCGG<br>CCATCACAAACTCCAAGGTGGAAGAGACGATTCTGGCCCGGCTGGGAGAAAAGGCACTGGCC<br>TACGGCACAGACCCCACTCTCCGGTGGTCGCAGAGAACCCAGGGAAAGGTTGATTCCGAGGT<br>TGTCAAGCGAATCTACAAGGAGTGGGTGATTCCACTCACCAAGAAGGTCGAGGTGGACTACC<br>TGCTCCGGCGGTTGGAGTAG	
ARO1	MFAEGQIQKVPILGKESIHIGYKMQDHIVSEIVANIKSSTYILVTDTNIEDLGYVESLKTKFEAAFAKD<br>GIKSRLLTYTVAPGETSKSRATKAAIEDWMLSKGCTRDTVILAVGGGVIGDMIGYVAATFMRGVRF<br>VQIPTTLLAMVDSSIGGKTAIDTPLGKNLVGAFWQPVNIFIDTSFLETLPVREFINGMAEVIKTAAFY<br>DAEEFTRLESASEIFLSTIKKRDAKDPRRVDLSPITDTIGRIVLGSARIKAAVVSADEREGGLRNLLNFG<br>HSIGHAYEAILTPYILHGECVAIGMVKEAELSRYLGILSPVAVARLAKCIKAYELPVSLDDATVKARSH	6

	GKKCPVDDLLRIMGVDKKNDGSTKKIVILSAIGKTHEQKASSVADKDIRFVLSEEVIVGEAPVGDKK<br>SYTVTPPGSKSISNRAFVLTALGKGPCKLRNLLHSDDTQHMLEAIELLGGASFEWEADGETLLVTGN<br>GGKLTAPAQELYLGNAGTASRFLTTAATLVQKGDKDHVILTGNKRMQERPIGPLVDALRSNGADI<br>AFQNAEGSLPLKIEAGVGLKGGLIEVAATVSSQYVSSLLMCAPYAQTPVTLSLVGGKPISQFYIDMTI<br>AMMADFGVVVTKDETKEHTYHIPQGVYTNPEEYVVESDASSATYPLAYAAMTGHTVTVPNIGSK<br>SLQGDARFAIDVLKAMGCTVEQTATSTTVTGVPNLKAIAVDMEPMTDAFLTACVVAAVSEGTTVI<br>TGIANQRVKECNRIEAMRVQLAKYGVVCRELEDGIEVDGISRSDLKTPVSVHSYDDHRVAMSFSLL<br>SSIMAAPVAIEERRCVEKTWPGWWDVLSGVFNVPLEGVTLAKTVSKAESGLSKPSIFIVGMRGAG<br>KTHLGAQAANHLGYEFIDLDQLLEKDLDTTIPQLIADKGWDHFRAEELRLLKQCLNDKSEGYVISCG<br>GGVVETPAARDALQTFKGVGGIVLHVHRPVSRILEYLNKDQSRPAFVDDLEAVWQRRKELYRSVS<br>SNVFFAPHCDSAEATAKVQQMLGAFLDRVTGKSEFVIPHKDQFTSFLSLTFPDVSIAATMLPSLSEG<br>CSALELRVDLLNENDEAIPSEEYVLSQLAILRQNVDLPILYTVRTKAQGGRFPDDKPVELANLVNLGL<br>KTAVELLDVELTYPAELVSSVGASRGYTKLLGSHHDFPGALNWSSLEWENMYARAEAVPVDVVKL<br>VGMAKSFSDNFALENFREAHTSSPLLAINMGSHGQLSRVTNTLLTPVTHADLPVAAAPGQLSVEEI<br>NQTRSTIGMFNKNLSFFIVGTPIGHSKSPILHNTMFKKLGLPYEYSRFKTDDAAAVNAKARALLAQG<br>NLGGISVTIPLKQDIIPFLDEVSPLAQQIGAVNTIIPGPNGTLKGDNTDILGLVNALTRFGANSLDKKT<br>ALIVGAGGTSLAAVHGLRSLGFAKILIANRTLSKAEAIADKFDNVEAVTLDSFVANKYTPSVIVSCVP<br>ATTFSMLDESNKLVSAALAASPKGLVLEAAYSAEATPLLKQVMDVEGWEFISGLYMLTEQGFEQF<br>RLWTGIPAPKEVGEKAVLGN*
YALI1_F16984g	ATGTTTGCCGAGGGTCAGATCCAAAAGGTCCCGATTCTGGGCAAGGAGTCCATCCACATTGGC<br>TACAAGATGCAGGACCACATTGTGTCTGAAATCGTGGCCAACATCAAGTCGTCCACCTACATT<br>CTGGTAACCGACACCAACATTGAGGATCTGGGCTACGTTGAGTCTCTTAAGACGAAATTCGAG<br>GCTGCGTTTGCCAAGGACGGCATCAAGAGCCGACTGCTGACCTACACCGTTGCGCCCGGAGA<br>AACCTCCAAGTCCAGAGCCACCAAGGCCGCCATTGAAGACTGGATGCTGTCCAAGGGCTGCA<br>CCCGAGATACGGTGATCCTGGCTGTGGGAGGCGGAGTGATCGGAGACATGATCGGCTACGT<br>GGCCGCCACCTTCATGCGAGGAGTGCGGTTTGTCCAGATCCCCACCACTCTGCTTGCCATGGT<br>TGACTCGTCCATTGGAGGCAAGACCGCCATTGATACCCCTCTGGGCAAGAATCTGGTCGGCGC<br>CTTCTGGCAGCCCGTCAACATTTTCATCGACACTTCTTTCCTCGAGACTCTGCCCGTTCGAGAGT<br>TCATTAACGGTATGGCGGAGGTCATTAAGACTGCGGCATTCTACGACGCCGAAGAGTTCACAC<br>GGCTCGAGTCCGCGTCGGAAATCTTCCTGTCCACTATCAAGAAGCGAGACGCCAAGGACCCCC<br>GACGAGTCGATCTGTCCCCCATCACCGACACCATTGGCCGAATTGTTCTCGGTTCTGCTAGAAT<br>CAAGGCCGCCGTTGTCTCTGCCGACGAGCGAGAGGGCGGTTTGCGAAACCTGCTCAACTTTG<br>GCCACTCCATTGGCCACGCCTACGAGGCCATTCTCACTCCTTACATTCTGCACGGCGAGTGTGT<br>GGCTATCGGTATGGTCAAGGAGGCCGAGCTGTCTCGATACCTGGGAATTCTCTCTCCCGTTGC<br>TGTGGCTCGTCTGGCCAAGTGCATCAAGGCCTACGAGCTGCCCGTGTCTCTGGACGACGCTAC<br>AGTCAAGGCCCGAAGCCACGGCAAGAAGTGCCCCGTTGATGATCTGCTTCGAATCATGGGCG<br>TCGACAAGAAGAACGACGGCTCCACTAAGAAGATTGTCATTCTGAGCGCCATTGGCAAGACC<br>CACGAGCAGAAGGCGTCTTCTGTGGCAGACAAGGACATTAGATTCGTTCTTTCCGAGGAGGT<br>CATTGTTGGAGAGGCTCCTGTTGGCGACAAAAAGTCCTACACCGTCACTCCACCTGGATCCAA<br>GTCCATTTCCAACCGAGCCTTTGTTCTGACTGCCCTGGGTAAGGGTCCTTGCAAGCTGCGAAA<br>CCTGCTGCATTCGGACGACACCCAGCACATGCTCGAGGCCATTGAGCTGCTTGGTGGCGCATC<br>GTTCGAGTGGGAGGCCGACGGTGAGACTCTGCTTGTCACCGGAAATGGCGGCAAGCTCACTG<br>CTCCCGCCCAGGAGTTGTACCTAGGAAACGCCGGTACTGCTTCTCGATTCCTTACCACCGCTGC<br>TACTCTGGTTCAGAAGGGAGACAAGGACCACGTGATCCTTACCGGAAACAAGCGAATGCAGG<br>AGCGGCCCATTGGGCCTCTTGTGGACGCTCTTCGATCCAACGGAGCCGACATTGCTTTCCAGA<br>ACGCCGAAGGCTCTCTCCCCCTCAAGATCGAGGCCGGTGTTGGACTCAAGGGTGGCTTGATTG<br>AGGTGGCTGCTACTGTTTCTTCTCAGTATGTCTCTTCTCTACTTATGTGTGCCCCCTACGCACAG<br>ACACCCGTCACTCTGTCTCTGGTTGGAGGCAAGCCCATCTCGCAGTTCTACATTGACATGACCA<br>TTGCCATGATGGCCGACTTCGGTGTGGTTGTCACCAAGGACGAGACCAAGGAGCACACCTAC<br>CACATTCCTCAGGGTGTGTACACCAACCCTGAGGAGTACGTGGTGGAGTCGGATGCTTCTTCA<br>GCCACCTACCCTCTTGCTTACGCTGCCATGACTGGCCACACCGTCACTGTTCCCAACATTGGCA<br>GCAAGTCTCTGCAGGGAGACGCCCGATTTGCCATTGATGTTCTCAAGGCTATGGGCTGCACCG<br>TTGAGCAGACCGCTACCTCGACCACTGTTACTGGTGTGCCCAACCTCAAGGCTATCGCTGTTG<br>ATATGGAGCCTATGACTGACGCATTCCTCACCGCCTGTGTCGTTGCCGCTGTGTCCGAGGGCA

	CCACCGTCATCACCGGCATTGCCAACCAGCGAGTAAAGGAGTGCAACCGAATCGAGGCCATG<br>CGAGTGCAACTGGCCAAGTACGGCGTCGTTTGCCGAGAGCTTGAGGACGGCATTGAGGTCGA<br>CGGAATTTCCCGATCAGATCTCAAGACCCCCGTCTCTGTGCACTCTTACGATGACCACCGAGTT<br>GCTATGTCTTTCTCGCTGCTCTCTTCTATCATGGCTGCTCCCGTGGCTATTGAGGAGCGACGAT<br>GTGTCGAAAAGACCTGGCCGGGATGGTGGGACGTGCTCTCCGGCGTGTTCAACGTTCCTCTG<br>GAGGGCGTAACGCTGGCCAAGACCGTTTCCAAGGCCGAGTCCGGACTTTCCAAGCCCTCCATC<br>TTCATTGTGGGCATGCGAGGTGCCGGAAAGACCCATCTGGGCGCCCAGGCCGCCAACCATCT<br>TGGCTACGAGTTCATTGATCTCGACCAGCTACTCGAAAAGGATCTGGATACCACCATTCCTCA<br>GCTGATTGCGGACAAGGGCTGGGACCATTTCCGTGCCGAGGAGCTGCGTCTGCTCAAGCAGT<br>GTCTCAATGACAAGTCCGAGGGCTACGTCATTTCCTGCGGTGGCGGAGTTGTTGAGACTCCTG<br>CTGCTCGAGACGCTCTTCAGACCTTCAAGGGTGTTGGTGGTATTGTTCTGCATGTCCACCGACC<br>CGTTTCCAGAATCCTCGAGTACCTCAACAAGGACCAGTCTCGACCTGCATTTGTGGACGATCT<br>GGAGGCGGTGTGGCAGCGACGAAAGGAGCTGTACCGATCTGTCTCTTCCAATGTCTTCTTTGC<br>TCCTCACTGCGACTCTGCCGAGGCCACTGCCAAGGTGCAGCAGATGCTGGGCGCCTTCCTGGA<br>CCGAGTCACCGGCAAGTCCGAGTTTGTGATTCCCCACAAGGACCAATTCACTTCCTTCCTGTCG<br>CTAACCTTCCCCGACGTGTCTATTGCCGCAACCATGCTCCCCTCGCTGTCTGAGGGTTGTTCTG<br>CTCTGGAGCTGCGAGTCGATCTGCTCAATGAGAATGACGAGGCCATTCCCTCCGAAGAGTAC<br>GTTCTCTCTCAGTTGGCGATTCTGCGACAGAATGTCGACCTCCCCATTCTTTACACGGTGCGAA<br>CCAAGGCTCAGGGAGGCCGTTTCCCCGACGACAAACCTGTGGAGCTGGCCAACCTGGTCAAC<br>CTGGGTCTCAAGACCGCCGTTGAGCTGCTGGACGTTGAGCTGACCTACCCTGCCGAGCTTGTT<br>TCGTCCGTCGGAGCTTCCAGAGGCTACACCAAGCTGCTGGGCTCTCACCACGACTTTCCCGGC<br>GCTCTCAACTGGTCTTCTCTCGAGTGGGAGAACATGTACGCCCGAGCCGAGGCTGTGCCTGTG<br>GACGTGGTCAAGCTCGTCGGTATGGCCAAGTCTTTCTCCGACAACTTTGCTCTGGAGAACTTCC<br>GAGAGGCCCACACCTCTTCTCCTCTTCTGGCTATCAACATGGGCTCCCATGGTCAGCTGTCTCG<br>AGTCACCAACACTCTGCTGACTCCCGTGACTCACGCTGACTTGCCTGTGGCTGCTGCTCCTGGC<br>CAGCTTTCCGTGGAGGAGATCAACCAGACCCGGTCGACCATCGGCATGTTCAACAAGAACCTG<br>TCCTTCTTCATTGTCGGCACTCCCATCGGCCACTCCAAGTCGCCCATTTTGCACAACACCATGTT<br>CAAGAAGCTGGGTCTGCCCTACGAGTACTCGCGGTTCAAGACTGATGATGCTGCTGCCGTCAA<br>CGCTAAGGCCCGTGCTCTGCTTGCCCAGGGCAACCTTGGAGGTATCAGTGTGACCATTCCTCT<br>CAAGCAGGACATTATTCCTTTCCTTGACGAGGTGTCTCCCCTCGCTCAGCAGATTGGAGCCGTC<br>AACACCATCATTCCTGGACCTAACGGGACTCTCAAGGGCGACAACACCGATATTCTCGGTCTG<br>GTGAACGCTCTGACTCGGTTTGGAGCCAACTCGCTGGACAAGAAGACTGCTCTGATTGTCGGA<br>GCCGGTGGCACATCTCTGGCTGCCGTGCACGGACTGCGATCGCTCGGCTTCGCCAAGATTCTC<br>ATTGCCAACCGAACTCTGTCCAAGGCCGAGGCCATTGCTGACAAGTTTGATAACGTGGAGGCT<br>GTGACTTTGGACTCGTTTGTGGCCAACAAGTACACTCCGTCTGTGATTGTGTCTTGCGTGCCAG<br>CCACCACTTTCTCGATGCTGGATGAGTCCAACAAGCTGGTGTCTGCCGCTCTGGCTGCTTCTCC<br>CAAGGGTCTGGTTCTTGAGGCTGCCTATTCTGCCGAGGCCACTCCTCTGCTCAAGCAGGTGAT<br>GGACGTCGAGGGCTGGGAGTTCATCTCCGGGCTCTACATGCTCACCGAGCAGGGCTTTGAGC<br>AGTTCCGGCTGTGGACCGGAATTCCCGCCCCCAAGGAGGTTGGAGAGAAGGCTGTTCTTGGT<br>AACTAA	
ARO2	MSTFGTLFKVTTYGESHCKSVGCIVDGVPPGMDLDESDIQPQLTRRRPGQSALTTPRNERDAVAI<br>QSGTEYGKTLGTPIAMLVQNKDQRPHDYSEMDDYPRPSHADYTYQEKYGIKASSGGGRSSARETI<br>GRVAAGAIADKYLAAVNDIEIVAFVSQVGDVSMDRSPSNEQWISTLEGVTREGIDSTGPMRCPDL<br>ALGEKMVKIVEEHRDSHDSVGGVVTCVIRNCPVGLGEPCFDKLEATLAHAMMSIPATKGFEFGSG<br>FAGAAMSGSKHNDMFYKDVASGRFRTRTNYSGGVQGGISNGENIYFNIAFKPPATISQEQATATY<br>AGKDGVLAAKGRHDPNVVPRAVPIVEAMAALVIADAHLIQESRRGAKSFF*	6
YALI1_D22237g	ATGAGCACTTTCGGCACGCTTTTCAAAGTCACCACCTACGGCGAGTCGCACTGCAAATCTGTG<br>GGCTGCATTGTTGACGGAGTTCCGCCCGGAATGGATCTCGATGAGTCCGATATTCAGCCCCAG<br>CTGACCCGTCGAAGACCTGGCCAGTCGGCTCTGACCACTCCTCGAAACGAGCGAGATGCGGT<br>TGCTATCCAGAGTGGAACCGAGTACGGCAAGACTCTGGGTACCCCCATTGCCATGCTGGTTCA<br>GAACAAGGACCAGCGACCCCACGACTACTCGGAGATGGACGACTACCCCCGACCTTCTCATGC<br>CGACTACACCTACCAGGAGAAGTACGGAATCAAGGCCTCTTCTGGAGGCGGCCGATCTTCTGC<br>ACGAGAGACTATTGGCCGAGTTGCTGCTGGAGCTATTGCCGACAAGTACCTGGCTGCTGTCAA	

	CGACATTGAGATTGTTGCCTTTGTGTCCCAGGTCGGCGATGTTTCCATGGACCGATCTCCCAGC<br>AACGAGCAGTGGATTTCCACTCTGGAGGGCGTCACTCGAGAGGGCATTGATTCCACCGGCCC<br>CATGCGATGTCCCGATCTGGCCCTCGGAGAGAAGATGGTCAAGATTGTGGAGGAGCACAGA<br>GACTCGCATGACTCCGTTGGAGGAGTTGTCACCTGTGTGATCCGGAACTGCCCCGTCGGTCTC<br>GGCGAGCCCTGTTTCGACAAGCTGGAAGCCACTCTCGCCCACGCCATGATGTCCATCCCCGCC<br>ACCAAGGGCTTTGAGTTTGGTTCTGGCTTTGCCGGAGCTGCCATGTCTGGATCCAAGCACAAC<br>GACATGTTCTACAAGGACGTGGCCTCCGGCCGGTTCCGAACCCGAACCAACTACTCCGGAGGT<br>GTCCAGGGCGGAATCTCCAACGGCGAGAACATCTACTTCAACATTGCCTTCAAGCCCCCGGCC<br>ACCATTTCTCAGGAGCAGGCCACTGCCACCTACGCCGGTAAGGACGGTGTGCTGGCTGCCAA<br>GGGCCGACACGATCCCAACGTTGTGCCCCGAGCCGTTCCCATCGTTGAGGCCATGGCCGCTCT<br>GGTCATTGCTGACGCCCACCTCATTCAGGAGTCCAGAAGGGGCGCAAAGTCCTTTTTCTAA	
ARO3	MPAMHNASNAQGDRNRTEDWRIRGYNPLTAPDLLQHEIPLTKQSKATILKGRQDACDILDGKDD<br>RIIVVVGPCSIHDPKAAMEYAERLKQISDKLSGELLIVMRAYLEKPRTTVGWKGLINDPDMDGSFKI<br>NKGLRVARDLFVKLTELNPIASELLDTISPQFLADLFSVGAIGARTTESQLHRELASGLSFPVGFKNGT<br>DGGIKVALDAIQAAAHPHHFLSVTKPGVVAIVGTDGNEDCFLILRGGSKGPNYDAEHVAEVKKQV<br>GETKGPRIMVDCSHGNSSKNHKNQPLVASNVAQQIAAGEKSICGLMIESNIHEGRQDICDNKED<br>MKYGVSVTDACINWEDTEKVLEELAQAVKTRRG*	6
YALI1_B26153g	ATGCCCGCTATGCACAACGCTTCTAACGCTCAGGGAGACCGAAACCGGACCGAGGACTGGCG<br>AATCCGGGGCTACAACCCTCTCACAGCCCCCGATCTGCTCCAGCATGAGATCCCTCTGACCAA<br>GCAGTCCAAGGCCACCATTCTCAAGGGCCGACAGGACGCCTGTGATATTCTGGATGGTAAGG<br>ACGACCGAATCATTGTTGTGGTTGGCCCCTGTTCCATCCATGACCCCAAGGCTGCCATGGAGT<br>ACGCCGAGCGACTCAAGCAGATCTCTGACAAGCTGTCTGGCGAGCTTCTGATCGTCATGCGAG<br>CCTACCTCGAAAAGCCTCGAACCACCGTTGGCTGGAAGGGCCTTATTAATGATCCTGACATGG<br>ACGGCTCTTTCAAGATCAACAAGGGCCTGCGAGTCGCTAGAGATCTCTTCGTGAAGCTCACCG<br>AGCTCAACCCCATTGCTTCTGAGCTTCTGGACACCATTTCTCCCCAGTTCCTGGCTGATCTCTTC<br>TCTGTCGGAGCCATTGGTGCTCGAACCACCGAGTCCCAGCTTCACCGAGAGCTTGCCTCTGGT<br>CTGTCTTTCCCCGTTGGTTTCAAGAACGGTACTGACGGAGGTATTAAGGTGGCTCTGGACGCC<br>ATCCAGGCCGCCGCCCACCCCCACCACTTCCTTTCCGTCACCAAGCCCGGTGTGGTTGCCATTG<br>TCGGCACCGACGGAAACGAGGACTGCTTCCTGATTCTGCGAGGAGGTTCCAAGGGCCCCAAC<br>TACGATGCTGAGCACGTCGCCGAGGTCAAGAAGCAGGTTGGAGAGACCAAGGGTCCCCGAA<br>TCATGGTAGACTGCTCGCACGGCAACTCATCAAAGAACCACAAGAACCAGCCTCTGGTCGCCT<br>CTAACGTGGCTCAGCAGATTGCTGCTGGTGAGAAGTCCATTTGCGGTCTTATGATTGAGTCCA<br>ACATCCACGAGGGTCGACAGGACATCTGCGATAACAAGGAGGACATGAAGTACGGTGTGTCT<br>GTCACCGATGCCTGCATCAACTGGGAGGACACTGAGAAGGTGCTCGAGGAGCTGGCCCAGG<br>CCGTCAAGACTCGACGAGGTTAA	
4-HPPD	MSPSVEVTPAHTPTSYEVTNSLDSYRGYDHVHWYVGNAKQAASFYITRMGFSPIAYKGLETGSRD<br>VTTHVVGNGQVRFAFSSALRTGEPQADEIHAHLVKHGDAVKDVAFEVDNVEQLFSAAVKKGVRV<br>ISEPKVLKDAHGSVTYAVISTYGDTTHTLIERGSYEGAFLPGFVDTSANKDPIAAFLPNIELMHIDHC<br>VGNQDWNEMDNACKYYEETLGFHRFWSVDDKDICTEFSALKSVVMASPNEKIKMPVNEPAVGK<br>KKSQIEEYIDFYDGPGIQHIALRTDCILDTVRDLRARGVEFISVPGSYYENMKERLAKSSLKLEEKFEDI<br>QALNILIDFDEGGYLLQLFTKPLMDRPTVFIEIIQRRNFEGFGAGNFKSLFEAIEREQAKRGNL*	7
YALI1_B28454g	ATGTCACCTTCCGTCGAAGTCACCCCTGCACACACACCCACCTCGTACGAGGTGACCAACTCGC<br>TAGACAGCTATCGGGGCTATGACCACGTCCACTGGTACGTTGGTAATGCCAAGCAGGCCGCCT<br>CCTTCTACATCACCCGAATGGGATTCTCTCCCATCGCCTACAAGGGTCTTGAGACTGGCTCTCG<br>AGACGTGACCACCCATGTCGTGGGCAACGGCCAGGTCAGATTTGCATTCTCGTCTGCCCTGAG<br>AACCGGAGAGCCCCAAGCCGACGAGATCCACGCCCATCTGGTCAAGCACGGTGACGCCGTCA<br>AGGATGTGGCCTTTGAGGTCGACAATGTGGAGCAGCTTTTCTCTGCTGCTGTCAAGAAGGGC<br>GTCCGAGTGATTTCCGAGCCCAAGGTGCTCAAGGACGCACATGGCTCCGTCACCTACGCCGTC<br>ATCTCTACCTACGGAGATACCACTCACACTCTGATTGAGCGAGGCAGCTACGAGGGCGCCTTT<br>CTTCCCGGATTTGTCGACACCTCCGCCAACAAGGACCCCATCGCGGCCTTCCTGCCCAACATTG<br>AGCTCATGCACATCGACCACTGCGTTGGAAACCAGGATTGGAACGAGATGGATAACGCATGC	

	AAGTACTACGAGGAGACTCTGGGCTTCCACCGGTTCTGGTCGGTCGACGATAAGGACATTTGC<br>ACCGAGTTCTCTGCTCTCAAGTCCGTGGTCATGGCCTCACCCAACGAGAAGATCAAGATGCCC<br>GTCAACGAGCCGGCCGTGGGCAAGAAAAAGTCCCAGATTGAGGAGTACATTGACTTTTACGA<br>CGGCCCCGGTATCCAGCACATTGCTCTGCGGACCGACTGTATCCTGGACACCGTCCGAGATCT<br>GCGGGCTCGTGGCGTGGAGTTCATTTCGGTCCCTGGATCTTACTACGAGAACATGAAGGAGC<br>GGCTGGCAAAGTCATCTCTGAAGCTCGAGGAGAAGTTTGAGGATATCCAGGCGCTCAACATT<br>CTGATTGATTTCGACGAGGGCGGATATCTGCTGCAGCTGTTCACCAAGCCTTTGATGGACCGG<br>CCCACCGTGTTCATTGAGATCATCCAGCGACGAAACTTTGAGGGTTTTGGCGCCGGCAACTTC<br>AAGTCTCTGTTTGAGGCCATTGAGCGGGAGCAGGCCAAGCGAGGCAACCTTTAA	
YALI1_B05024p	MKFTFAAVTAALASSAMALGGLGVDLGVKRESDGECKNAGDYKADLEALKGLTDTIRIYAAGDCD<br>ALRELGPVAEAANFKLMIGVWPNDDNHFASEQFALKSYLPWLSKSTVPYITVGSEALYRKDMTPQ<br>QLADKINDIKNQLKGIKDKNGQTFDVPVGTVDSWNVIVDGYSSPAVKAADVVFANAFSYWQGQ<br>TMANASYSFFDDIMQALQTIQTTKGTTDIDFWVGETGWPTDGGAFGDSQPGVKQAAQFWQEG<br>ICAIRAWGINTLVFEAFDETWKPDTKGDNGEEVSGVEKYWGVYDSNLKPKFDTTCKFD*	This study
YALI1_B05024g	ATGAAGTTCACATTTGCTGCCGTTACCGCCGCGCTGGCCTCGTCCGCCATGGCCCTCGGAGGC<br>CTCGGAGTCGACCTCGGAGTCAAGCGAGAGTCCGACGGAGAGTGCAAGAACGCCGGCGACT<br>ACAAGGCCGATCTTGAGGCTCTCAAGGGTCTCACCGACACCATCCGAATCTACGCCGCCGGCG<br>ATTGCGACGCCCTGCGAGAGCTCGGCCCTGTTGCTGAGGCCGCTAACTTCAAGCTCATGATTG<br>GTGTGTGGCCCAACGACGACAACCACTTTGCCTCCGAGCAGTTTGCTCTCAAGAGCTACCTGC<br>CCTGGCTCTCCAAGTCCACCGTGCCCTACATCACCGTGGGCTCCGAGGCTCTGTACCGAAAGG<br>ACATGACTCCCCAGCAGCTGGCCGACAAGATCAACGACATCAAGAACCAGCTCAAGGGCATC<br>AAGGACAAGAACGGCCAGACCTTTGACGTCCCCGTCGGTACCGTCGACTCCTGGAACGTGATT<br>GTCGATGGCTACTCCAGCCCCGCCGTCAAGGCCGCCGACGTTGTCTTCGCCAACGCCTTCTCCT<br>ACTGGCAGGGACAGACCATGGCTAACGCCTCTTACTCCTTCTTCGATGACATCATGCAGGCTCT<br>CCAGACCATCCAGACCACCAAGGGTACCACCGACATTGACTTCTGGGTTGGTGAGACCGGAT<br>GGCCCACCGATGGCGGTGCTTTCGGCGACTCTCAGCCTGGCGTGAAGCAGGCTGCTCAGTTCT<br>GGCAGGAGGGTATCTGTGCCATCCGAGCCTGGGGTATCAACACTCTGGTTTTCGAGGCTTTCG<br>ACGAGACCTGGAAGCCCGACACTAAGGGTGACAACGGTGAGGAGGTCTCCGGCGTTGAGAA<br>GTACTGGGGTGTTTACGACTCCAACCTCAAGCCCAAGTTCGACACCACTTGCAAGTTTGACTA<br>A	
YALI1_B18845p	MLAFVLLLTMLLAAALADPFSDKDAYKHSPPYYPAPEIGRVPTDLRWRAALKVAQGMVANMTLL<br>EKVNITTGTGWEMGPCVGNTGTVERLGIKSLCLQDGPLGIRFADLITTFPAGITIASTFSRQLVRER<br>GAAMGRENRRKGVDITLSPVVGPLGRHANGGRIWEGFSADPYLAGKLAAEAVTGIQGQNVMAV<br>VKHMVGNEQEHFRQLGEWQGFGFKDLKQPLSSNIDDRTLNEAYLWPFADAVRANVGSVMCSY<br>QQINGSQGCQNAHILNGKLKEEMGFQGFVMSDWLAQRSGVASVLAGLDMSMPGDGLVWAD<br>GVPLMGYELTRSVLNGTIDESRVDDMVTRILTPILYLSITPTDPNFSSWTNDTTSYKYYGAKAGGNV<br>TVNRHIDVRDQYTTKAALDGANAALVLLKNEKKTLPLNPTNIGNLNIFGIGSKTGPLGAVCGENM<br>QCSDGALIEGWGSGSVYPTDYQSPYDAIKERASKDNITIGGTTQSWGNLSNVEILSAAADASVVFV<br>LSDSGESTGIVDGNIGDRNNLTLWHNGDEVVKAVASKNPNTIVVVTTVGPVNLEKWIDNPNVTA<br>VLLTGPAGDFGGRAAASILFGDIAPSGKLPFTIAKNDTDYIPLTTKIPEDGLPQDYFTEGTLLDYKRFD<br>ENQVTPRFEFGYGLSYSNITVENLEARYAFPSIPEFLPTPFAPSNPNKPKNAFTPHANESVFPSDIDP<br>LNKYVYPYLNDTSEIFSNETHYPYPEGYSSEQSNSTNINGGAVGGNPALWLSAVYIVHSVSNYGPY<br>DTGVVTQMYIAFPQDNDDLKTAPRQLRGFERSELKVGERQGILYDVQWRDLAVWDVKLQSWRV<br>QRGEYKVYVGHSSRDFVLTTSFTLK*	This study
YALI1_B18845g	ATGCTCGCATTCGTCCTACTGCTGACGATGCTGCTCGCAGCAGCGCTTGCTGACCCGTTCTCAG<br>ATAAAGACGCTTACAAACACAGCCCTCCATACTACCCTGCTCCGGAGATTGGCAGAGTTCCCA<br>CCGACCTGCGATGGAGAGCTGCCTTGAAGGTGGCCCAGGGTATGGTCGCTAACATGACACTG<br>CTTGAGAAGGTGAACATCACCACCGGTACGGGCTGGGAGATGGGTCCTTGTGTTGGAAACAC<br>TGGTACCGTCGAACGACTGGGTATAAAATCGCTGTGCCTTCAAGACGGCCCTCTTGGGATTCG<br>ATTTGCTGACCTCATTACCACATTCCCTGCTGGTATCACTATTGCCTCTACCTTCTCTCGACAGC<br>TGGTTAGAGAGCGAGGTGCTGCTATGGGACGGGAGAATAGACGCAAGGGAGTGGATATCAC	

	TCTCAGCCCTGTGGTTGGACCACTGGGAAGACATGCTAACGGAGGTCGAATCTGGGAGGGCT<br>TCTCTGCTGACCCCTACCTTGCTGGAAAGCTCGCCGCCGAGGCCGTGACAGGTATACAGGGCC<br>AGAACGTCATGGCTGTGGTAAAGCATATGGTTGGAAACGAGCAGGAACATTTTCGACAACTT<br>GGCGAGTGGCAGGGATTCGGATTCAAGGATCTGAAGCAGCCCCTCTCTTCAAACATCGACGA<br>CCGAACTCTTAACGAAGCGTACCTTTGGCCCTTTGCTGATGCTGTTCGAGCCAATGTTGGATCT<br>GTCATGTGTTCCTATCAGCAGATCAATGGCTCTCAGGGTTGTCAAAACGCCCACATTCTGAAC<br>GGTAAGCTCAAGGAGGAAATGGGTTTCCAGGGCTTTGTCATGTCCGACTGGCTTGCCCAGCG<br>AAGTGGCGTGGCGTCTGTTCTTGCTGGTCTCGATATGAGCATGCCTGGAGACGGTCTTGTCTG<br>GGCGGACGGTGTTCCGCTCATGGGATACGAGTTGACCAGGAGTGTGCTGAATGGAACCATTG<br>ATGAAAGCCGAGTGGACGACATGGTTACCCGAATCCTTACCCCCATACTGTATCTCTCAATTAC<br>TCCGACCGACCCCAACTTCAGCTCTTGGACCAACGACACTACTAGCTACAAGTACTACGGAGC<br>CAAGGCTGGTGGAAACGTCACTGTTAACCGACATATTGATGTGAGAGACCAGTACACTACCAA<br>GGCTGCTCTTGATGGAGCAAACGCTGCGCTTGTTCTTCTCAAGAATGAGAAGAAGACTCTTCC<br>TCTGAACCCTACCAATATTGGAAACCTCAACATTTTCGGTATTGGTTCTAAAACCGGCCCACTT<br>GGAGCTGTCTGTGGAGAAAATATGCAGTGTAGTGATGGCGCCCTTATTGAGGGATGGGGGTC<br>CGGTTCCGTCTACCCTACCGATTATCAATCTCCTTACGACGCCATTAAGGAGAGAGCCTCCAAG<br>GACAACATCACCATCGGAGGCACTACGCAATCTTGGGGTAACCTGTCGAACGTTGAGATCCTT<br>TCAGCTGCTGCCGACGCCAGTGTCGTCTTTGTTCTTTCCGACTCCGGTGAGAGTACTGGTATTG<br>TTGACGGCAACATTGGGGATCGAAACAACTTGACGCTGTGGCACAATGGAGACGAGGTTGTC<br>AAGGCTGTGGCATCTAAGAACCCCAACACTATCGTTGTTGTTACCACTGTCGGCCCTGTGAAC<br>CTCGAAAAGTGGATCGACAACCCAAACGTCACTGCCGTGCTTCTCACTGGACCCGCTGGTGAC<br>TTTGGAGGAAGAGCTGCAGCCTCTATTCTCTTCGGCGACATCGCCCCTTCAGGAAAACTCCCTT<br>TCACTATTGCCAAGAATGACACCGACTACATTCCTCTTACTACTAAGATCCCTGAAGACGGCCT<br>TCCTCAAGACTATTTCACTGAGGGTACTCTTTTGGACTACAAACGGTTCGACGAGAACCAGGT<br>GACTCCTAGGTTTGAATTTGGCTACGGTCTGTCTTACTCTAACATTACGGTGGAGAATCTCGAA<br>GCCCGGTATGCTTTCCCTAGCATTCCTGAGTTCTTGCCCACTCCCTTTGCCCCTTCGAACCCCAA<br>CAAGCCTAAGAACGCATTTACTCCTCACGCCAATGAGTCCGTCTTCCCCAGTGACATTGATCCT<br>TTGAACAAGTACGTCTATCCATACCTGAACGATACCTCGGAGATCTTCTCTAACGAGACCCATT<br>ATCCCTATCCTGAGGGGTACTCCAGTGAGCAGTCCAACAGTACCAACATTAACGGCGGGGCT<br>GTCGGAGGCAACCCTGCTCTGTGGCTCTCTGCAGTCTACATTGTCCACAGCGTGTCTAACTATG<br>GTCCCTATGATACTGGAGTGGTCACCCAGATGTACATTGCCTTCCCTCAGGATAACGACGATCT<br>TAAAACCGCTCCTAGACAGCTTCGAGGATTCGAACGGTCCGAGCTCAAGGTGGGAGAACGGC<br>AGGGAATTCTATACGATGTTCAATGGCGAGATCTCGCGGTCTGGGATGTCAAACTTCAGAGCT<br>GGCGGGTCCAACGAGGAGAGTACAAGGTTTACGTAGGCCACAGTTCGCGAGACTTTGTTCTG<br>ACCACCAGCTTCACTCTCAAGTAA	
YALI1_B18887p	MRYTNTQALVGVCLLLLLACVAKAQREPPHPHPRYPMAPPPPPHYPYYYPNRHGAYRRTRQAAG<br>RSPVGLRNDRRPKARLDNHKKKTAQHHDGEKKAKPKSDTIIIRIASNVGGDIPKLSDPEPFDSNPFV<br>NLTNYMHSPTYYPTPMAGRINDHCWRSAFLKAKAFVTQLTNEEKANLTTDFSTADSPWYGETGEI<br>PRLNLSSLRLQWGLHGVGGDSHFTTFLPAGITTASTFNKGLMYSRGAIIGKEARKKGMDIVLGPLID<br>PTGRSAAGGRNWEGFGPDPYMAGVVATESVTGIQDQGVVATVMHYVGYSQEHFRNLEEWQA<br>HGYNNLTSSGSSFIDDRTMNEVYIWPFANAVKANAGAIMCAPQKLNNTQGCKNSYMMNYKLKR<br>ELGFQGFVLSAGMSQNEPPAALAGMDMSMPRLKASENKLREMMLRNYDKDGFPQSRLDDMA<br>TRVLTSYYYVTQHRNGHQSFVGDEENLSNKSAYDNILVDVRDGFHYRVALEIALEGIVMLKNDDSA<br>LPIEGMRTIGVLGAAANLGPSGSKCDDKFGCHDGAIFQGWGDGGVNPPFVVTPYEAVNARAAR<br>DRIVVRSNFDSWDLNQAEAVASSTDANIIFVAANSGEGNHVVDDNKGDRKNFTLWHNGDELIKK<br>AVEVNDHNIVVVTAVGPVDMEKWIEHPHVKAVLFTGPGGEEAGAALAHVLFGDFNPSGKLPFTI<br>ARNVNHYIPIETEVPRDGIPKAYFGEMSLTDYKWFDQNLISPRFEFGFGMSFSEFSYSDMKITTQR<br>NPSLTLGAPPDYWGKDGNATTAKAFQQEFKAASGDFQFPSGYNDDHNDDPVLNGGAVGGNP<br>MLWDVMYQVAVSVTNHGPFDGAVVSQLYVSFPQDDQALRTAPKQLRGFSKTSLVVGETANVLF<br>DLMWRDLAVWDVVKQTWVVQRGDYDVFIGGSSRQLETLGRITIA*	This study
YALI1_B18887g	ATGAGATACACACAAATACCCAAGCGCTGGTGGGTGTCTGTCTACTCCTGCTGCTGGCTTGCGTG<br>GCCAAAGCACAGAGAGAACCCCCTCACCCTCACCCCCGATACCCAATGGCTCCACCCCCGCCT<br>CCTCACTACCCTTACTACTACCCCAACCGTCATGGAGCGTACAGACGTACTCGTCAAGCAGCAG	

	GCAGATCCCCCGTGGGTCTCCGAAATGACCGACGTCCCAAAGCGCGACTCGATAACCACAAG<br>AAGAAGACCGCCCAGCACCACGACGGAGAGAAGAAGGCCAAGCCCAAGTCCGATACCATTAT<br>CATCCGAATTGCAAGCAATGTCGGTGGCGATATTCCCAAGCTCTCAGACCCGGAGCCCTTCGA<br>TTCCAACCCCTTTGTGAATCTCACAAACTACATGCATTCCCCCACCTATTACCCCACCCCCATGG<br>CTGGTCGGATCAATGATCACTGCTGGCGATCGGCGTTTCTCAAGGCCAAGGCGTTTGTCACCC<br>AACTCACGAACGAAGAGAAGGCTAACCTGACCACTGACTTCAGCACTGCAGATTCCCCTTGGT<br>ATGGCGAGACTGGCGAGATTCCCCGACTCAACTTGTCCTCTCTGCGTCTCCAATGGGGTCTCC<br>ATGGTGTCGGCGGCGACTCTCACTTCACCACCTTCCTTCCCGCCGGTATAACCACAGCATCTAC<br>CTTCAACAAAGGACTGATGTACTCTCGTGGTGCCATCATTGGGAAGGAGGCTCGGAAGAAGG<br>GCATGGACATCGTTCTCGGTCCTCTCATTGATCCCACTGGTCGAAGTGCCGCTGGCGGACGAA<br>ACTGGGAGGGATTCGGTCCTGATCCTTACATGGCTGGTGTCGTTGCAACTGAGTCCGTCACTG<br>GTATCCAAGATCAGGGAGTTGTTGCTACTGTGATGCACTACGTTGGGTACAGCCAGGAGCACT<br>TCCGAAACTTGGAAGAGTGGCAGGCCCACGGTTACAACAACCTCACATCCTCTGGCTCCTCAT<br>TCATCGATGACAGAACAATGAACGAGGTGTACATCTGGCCCTTCGCCAACGCTGTCAAGGCTA<br>ATGCCGGTGCTATCATGTGTGCCCCTCAAAAGCTCAACAACACCCAGGGCTGCAAGAACTCCT<br>ACATGATGAATTACAAGCTCAAAAGAGAGTTGGGATTCCAAGGGTTCGTCCTCTCCGCGGGTA<br>TGTCTCAGAACGAACCCCCTGCTGCGCTCGCCGGTATGGATATGTCAATGCCTCGGCTCAAGG<br>CGTCTGAAAACAAGCTGCGTGAAATGATGCTCCGAAATTATGACAAAGACGGATTCCCTCAAT<br>CACGCCTAGACGACATGGCTACACGAGTTCTCACCTCCTACTACTACGTTACACAGCACCGAAA<br>CGGCCACCAGTCCTTTGTCGGCGACGAGGAGAATCTCTCCAACAAGTCAGCCTACGACAACAT<br>CTTAGTTGATGTTCGTGATGGTTTTCACTACCGGGTCGCTCTCGAGATCGCTCTCGAAGGTATC<br>GTTATGCTAAAGAATGATGATAGTGCCCTTCCTATCGAGGGCATGAGAACCATTGGAGTGCTG<br>GGAGCCGCTGCCAACCTTGGACCCAGTGGCTCCAAGTGCGACGACAAGTTTGGGTGCCACGA<br>TGGTGCTATCTTCCAAGGATGGGGAGACGGTGGCGTCAACCCTCCGTTCGTTGTGACCCCCTA<br>CGAGGCTGTGAACGCCCGTGCTGCTCGAGACCGAATTGTTGTTCGGTCCAACTTCGATTCTTG<br>GGATCTCAACCAGGCCGAGGCCGTTGCCAGCAGCACTGATGCCAATATCATCTTCGTTGCCGC<br>CAACTCCGGCGAGGGTAACCACGTGGTGGATGATAACAAGGGCGACAGAAAGAACTTCACCC<br>TCTGGCACAATGGCGACGAGCTCATCAAGAAGGCTGTTGAGGTCAACGACCACAACATTGTC<br>GTTGTCACTGCTGTTGGTCCTGTTGACATGGAGAAGTGGATCGAGCACCCGCATGTCAAGGCA<br>GTTCTGTTCACTGGCCCTGGCGGCGAGGAGGCTGGGGCTGCTCTTGCTCACGTGCTCTTTGGA<br>GATTTCAACCCCAGTGGTAAGCTTCCCTTTACCATTGCACGGAACGTCAACCACTACATCCCCA<br>TTGAGACCGAGGTACCTCGGGATGGGATTCCCAAGGCTTACTTTGGTGAGATGAGCTTGACT<br>GATTACAAGTGGTTTGATCAGAACCTCATCTCTCCTCGATTCGAGTTTGGTTTCGGCATGTCCT<br>TCTCTGAGTTCTCATACTCTGACATGAAGATTACCACACAGAGAAACCCCAGTCTCACCCTTGG<br>AGCTCCCCCGGACTACTGGGGTAAAGACGGCAACGCGACTACTGCTAAGGCGTTCCAGCAAG<br>AGTTCAAGGCCGCTTCAGGGGACTTCCAGTTTCCTTCTGGCTATAATGATGACCACAACGACG<br>ATCCTGTCCTGAATGGTGGCGCTGTGGGTGGTAATCCCATGCTCTGGGATGTCATGTACCAAG<br>TTGCTGTGAGCGTGACCAACCACGGCCCCTTTGATGGTGCAGTCGTGTCACAGCTGTATGTCA<br>GCTTCCCTCAGGATGACCAGGCTCTCCGGACAGCCCCCAAACAGCTGCGAGGCTTCTCTAAGA<br>CTTCTCTTGTTGTTGGCGAGACAGCCAACGTTCTTTTTGATCTCATGTGGCGTGATCTCGCAGT<br>TTGGGATGTTGTCAAGCAGACCTGGGTGGTACAGCGGGGCGACTATGATGTGTTCATCGGTG<br>GTTCGAGTCGGCAGTTAGAAACCCTTGGTCGCATCACGATTGCTTAA	
YALI1_B23300p	MLQFLLLLNLALSASARVIHKKVTVVNDVVVTPSTTRYVTVTGGQSEHVTLDQTIESVETVFVAHTV<br>PTTVITGTLISTVQSIITANPVTQTDSYPTTADAPQININTLTEPKTLDPFEGVVTDAEPIPTEATVASS<br>ASATPTATPTQSSFSVSIRSSSTPGPVESTPGVVSIPDGPSTTSALISSSVVASSVASSVASSASAAASS<br>SAAAPKPKPNPPPAYNGDLFAAIDTSAPPGVFKQEPLNIQIPNGAVKELGQSPVHTNKFYFNMFLG<br>DRTMPVYTQPYSVWWSKTDKFPGIGVSYSQAKDKVYGPQKTNPMEYMLNPVGIMSFVFSAKEF<br>TTDNMEMQLSDPDTFSATTTVSTGDGSMELPLVQGMGFVTAKYNGLTPLLLTQVGFDSVVKGN<br>SPVQGTLKYVAKLFNGVTWTIYVTTSDPDFKFLFPDPHTIKTHSTKPCVIQMATTGDADSDAGSAV<br>SGFDKAAGSYPVSASLNGQANGGSAQYSIDYKTEGSSASGSTLLFALSHHKSSMVSQSGAVSGPKI<br>QSTNKGPMYAYVANSLTLAETLETDLQFLPWSQVSGFAGKATLTAAQQKLIAEVANSELKQDIGS<br>QTNLDTNYFSGKALDKFAYILLVLSDMIEDETTTKSVLDQLKKAFAVFTKNTQVSPFIYDTLFKGVTS<br>GAAQASGDSGADFGSPYYNDHHFHYGYFLHAAAVIGHVDAKYGDGQWVNENKDWVNSLIRDT<br>ANPSKDDSYFPVYRSFDWFSGHSWAKGLFPAADGKDEESTSEDYNHAYGMKLWGNVVGDKAM	This study

	EARGDLMLAVMKRSMNDYFYMKDDNKIQPEQLIGNKIPGITFENKLDYTTYFGTNPEYIHGIHMIP<br>VTPVSSLLRDPTFVQEEWEQNVSKFIGDVNSGWLGILHSNQALYDPKSAYEFFSQDNFQTLWLDG<br>GASRTWYLAYSAAVSA*	
YALI1_B23300g	ATGTTGCAGTTCCTCTTGCTGCTCAACCTCGCCCTGTCGGCATCTGCTCGAGTCATCCACAAAA<br>AGGTCACTGTGGTCAACGATGTGGTTGTCACTCCTTCTACAACCAGATACGTGACCGTTACTG<br>GCGGCCAGTCCGAGCACGTGACTCTTGATCAGACTATCGAGTCCGTCGAGACTGTGTTTGTGG<br>CCCACACCGTTCCCACAACCGTCATTACCGGCACTCTCATCTCCACCGTCCAGTCTATTATCACT<br>GCCAACCCGGTGACCCAGACTGATTCGTACCCCACCACCGCAGATGCTCCCCAGATCAACATC<br>AACACTCTGACCGAGCCCAAGACCCTGGATCCCTTTGAGGGAGTTGTAACTGATGCTGAGCCC<br>ATTCCCACAGAGGCCACCGTCGCTTCTTCCGCCTCTGCAACCCCCACCGCTACTCCTACTCAGTC<br>TTCCTTCTCGGTGTCTATCCGGTCTTCTTCTACTCCTGGTCCTGTTGAGTCCACTCCCGGTGTTG<br>TTTCCATCCCTGATGGTCCCTCCACCACTTCTGCTCTGATCTCTAGCTCCGTCGTTGCATCTTCC<br>GTTGCATCTTCCGTTGCCTCTTCTGCTTCCGCCGCTGCTTCGTCTTCCGCCGCTGCTCCCAAGCC<br>CAAGCCCAACCCCCCTCCTGCTTACAATGGCGACCTGTTCGCAGCCATTGACACCTCTGCCCCT<br>CCCGGAGTTTTCAAGCAGGAGCCTCTGAACATCCAGATCCCCAACGGCGCCGTCAAAGAGCTT<br>GGTCAGTCCCCCGTCCACACCAACAAGTTCTACTTCAACATGTTCCTCGGTGACCGAACCATGC<br>CCGTCTACACCCAGCCCTACTCGGTGTGGTGGTCCAAGACCGACAAGTTCCCCGGTATCGGTG<br>TCTCTTACTCCCAGGCTAAGGACAAGGTCTACGGCCCCCAGAAGACCAACCCCATGGAGTACA<br>TGCTGAACCCCGTTGGAATCATGTCCTTTGTCTTCTCCGCCAAGGAGTTCACCACCGACAACAT<br>GGAGATGCAGCTGTCTGACCCCGACACTTTCTCTGCCACCACCACTGTTTCTACCGGTGACGGC<br>TCCATGGAACTTCCTCTTGTTCAGGGAATGGGTTTTGTCACTGCCAAGTACAACGGTCTGACTC<br>CTCTTCTGCTGACCCAGGTCGGCTTTGACTCCGTCGTCAAGGGCAACTCCCCCGTGCAGGGCA<br>CCCTTAAGTACGTGGCTAAGCTCTTCAACGGAGTCACTTGGACCATCTACGTGACCACCTCTGA<br>TCCTGACTTCAAGTTCCTCTTCCCCGACCCTCACACCATCAAGACTCACTCCACCAAGCCCTGTG<br>TGATTCAGATGGCCACCACTGGTGACGCTGACTCTGATGCTGGCTCTGCTGTGTCTGGCTTCG<br>ACAAGGCAGCTGGTTCCTACCCCGTTTCTGCCTCTCTCAACGGCCAGGCTAACGGAGGTTCTG<br>CTCAATACTCCATCGACTACAAGACGGAGGGATCTTCTGCTTCTGGCTCAACTCTGCTGTTTGC<br>TCTGTCTCACCACAAGAGCTCCATGGTTTCTCAGTCCGGTGCTGTTTCCGGCCCTAAGATCCAG<br>TCCACCAACAAGGGTCCTATGTACGCCTACGTGGCCAACTCTCTGACCCTCGCTGAGACCCTCG<br>AGACTGATCTGCAGTTCCTTCCCTGGTCCCAGGTGTCTGGATTTGCTGGTAAGGCCACCCTGAC<br>TGCTGCGCAGCAGAAGCTGATTGCCGAGGTGGCCAACTCAGAGCTCAAGCAGGATATTGGAA<br>GCCAGACCAACCTGGACACGAACTACTTCTCCGGCAAGGCTCTGGACAAGTTCGCCTACATTC<br>TGCTGGTTCTGTCTGATATGATTGAGGATGAGACCACCACCAAGTCTGTTCTGGACCAGCTCA<br>AGAAGGCCTTCGCCGTCTTCACTAAGAACACTCAGGTATCTCCCTTCATCTACGACACTCTGTT<br>CAAGGGAGTCACCTCCGGTGCTGCCCAAGCCTCTGGCGACTCTGGTGCTGACTTTGGTTCTCC<br>TTACTACAATGACCACCACTTCCACTACGGCTATTTCCTTCATGCCGCAGCTGTGATTGGCCAT<br>GTTGATGCCAAGTACGGAGATGGTCAGTGGGTCAACGAGAACAAGGACTGGGTCAACTCTCT<br>TATCCGAGACACCGCCAACCCTTCCAAGGACGACTCTTACTTCCCTGTCTACCGATCTTTTGACT<br>GGTTCTCCGGCCACTCTTGGGCCAAGGGTCTCTTCCCTGCTGCCGATGGTAAGGACGAGGAGT<br>CAACCTCTGAGGACTACAACCACGCCTACGGAATGAAGCTGTGGGGTAACGTTGTCGGCGAT<br>AAGGCTATGGAGGCCCGAGGAGACCTGATGCTGGCTGTCATGAAGAGATCTATGAATGACTA<br>TTTCTACATGAAGGACGACAACAAGATCCAGCCCGAGCAGCTCATTGGCAACAAGATCCCCGG<br>TATCACCTTTGAGAACAAGCTGGACTACACCACCTACTTTGGCACCAACCCCGAGTACATCCAC<br>GGTATCCACATGATCCCCGTCACTCCTGTCTCTTCTCTGCTCCGAGACCCTACATTCGTCCAGGA<br>GGAGTGGGAGCAGAACGTGTCCAAGTTCATTGGAGACGTCAACTCTGGATGGCTCGGAATCC<br>TACACTCTAACCAGGCTCTTTACGACCCCAAGAGTGCCTACGAGTTTTTCTCTCAGGACAATTT<br>CCAGACTCTTTGGCTTGATGGAGGTGCTTCTCGAACCTGGTATCTCGCCTACTCTGCCGCAGTG<br>TCCGCCTAA	
YALI1_D22997p	MIAKIPLLPFVVGALAVSNSTDSTLTPTPSHQLNSHQTQLSPNSTLTNSTLTNFTNSTHVPFTSPDFY<br>PTPEIGTLDADKRWRAALNESLEILSQLTLVEKVNITTGLGWGGGTCVGNTGGVPRLGLKGLCLQD<br>GPLGIAQTDYVTVFPCGIAMAATFDRNLVHQRGTAIGQEAKAKGVDVHLGPVVGPLGRHATGGR<br>NWEGFSPDPYLAGKLVSEAIRGIQSENVMATVKHFIGNEQEHYRLYSEWARFGFDNLTTSVSSNID<br>DRTMHEAYLWPFADAVKAGVASVMCSYQQINGSSGCQNSATLNGKLKSELGFQGFVVSDWQA	This study

QLSGVSNALAGLDMSMPGNDVDGNIFWGPDLTKMVANGTLPESRLDDMVLRILTATIYTGIDER
EPTFSAFTTETFGNPNPVLMFNINYTSTLVNLHLDTRTAFSSRVALEGAEAAAVLLKNDGILPLQGP
ENVGVFGVGSQIGPKGAYCGFSMQCSDGALIEGWGSGTANPTEYTSPYEALRQRANQAGGHVI
GTTESWNMTLPLIMADNSDVNIIYVLSNSGEGGSTVLDNMGDRNNVSLWHNGDELITTLANSSR
NNIVVVTTVGQVDLEPWISHPNVSAVVLTGPAGAYGGKAMAEVLYGDVNPSGKLPYTIAKDPQD
YIPVVVDIPEDGAPQAYFDEGVYMDYKMFDKLNKTVRFEFGYGLSYSDFEFGDLDVVVDEFISELLP
SPPRPIIVDKPSSTSSNSSHGDLKAPKGFKPIRGVVYPWIDVNSLTAGEALSGVSPALATLLGLGKPD
TCAVPAKHETGDDDKDSKSDDKTKRDTESDAADDEIETVSTSNETAVETTIGMKNTSEPFHFANG
TGTSTNAAGGVGGNPSLWKTVATVSHTIRNLGPYPGAAVTQMYIAFPQDDIDSPLIQLRGFDKTR
VLDVGELETNSYDILWRDLAVWDVVIQSWRVQRGEYKIYVGNSSRDFVLIESFTLQ*

YALI1_D22997g

ATGATTGCAAAAATACCCCTACTTCCGTTCGTGGTAGGAGCTCTGGCTGTGTCCAACTCCACCG
ACTCCACTCTCACCCCGACTCCATCTCACCAACTCAACTCTCACCAAACTCAACTCTCACCAAAC
TCAACTCTCACCAACTCAACTCTCACCAACTTCACCAACTCTACCCATGTTCCCTTCACTTCTCCC
GATTTCTATCCTACTCCCGAAATTGGCACTCTGGACGCGGACAAGCGGTGGAGAGCTGCTCTG
AACGAGTCCCTGGAGATTCTGTCTCAGCTGACTCTTGTCGAAAAGGTCAACATCACCACAGGT
CTTGGTTGGGGAGGAGGCACCTGTGTCGGCAACACTGGCGGAGTTCCCAGACTCGGTCTTAA
GGGTCTCTGTCTCCAGGACGGACCTCTGGGAATTGCTCAGACCGACTACGTGACTGTTTTCCC
CTGCGGAATTGCCATGGCGGCCACCTTTGACAGAAACCTGGTCCACCAGAGAGGCACCGCCA
TTGGACAGGAGGCCAAGGCCAAGGGAGTCGATGTCCATCTGGGACCCGTCGTGGGACCTCTG
GGACGACACGCAACGGGCGGCCGAAACTGGGAGGGCTTTTCTCCGGACCCCTACCTGGCCGG
AAAGCTCGTGTCCGAAGCTATCCGAGGTATCCAGTCCGAGAACGTCATGGCCACCGTCAAACA
CTTCATTGGCAATGAGCAGGAGCACTACCGGCTCTACTCCGAGTGGGCTCGTTTCGGATTCGA
CAACCTCACCACTTCGGTATCCTCCAACATTGACGATCGAACCATGCACGAGGCCTACCTGTG
GCCCTTTGCCGACGCCGTCAAGGCCGGAGTGGCTTCAGTCATGTGTTCGTACCAGCAGATCAA
CGGCTCGTCGGGCTGCCAAAACAGCGCCACGCTCAACGGCAAGCTCAAGTCCGAGCTCGGGT
TCCAGGGCTTTGTGGTGTCGGACTGGCAGGCCCAGCTCAGCGGAGTGTCCAATGCTCTAGCC
GGACTGGACATGAGCATGCCTGGTAACGACGTGGACGGAAACATCTTCTGGGGACCGGACTT
GACCAAAATGGTGGCTAACGGCACTCTTCCCGAGTCCAGACTCGACGATATGGTGCTGCGAAT
TCTCACAGCCACCATCTACACAGGCATCGACGAAAGAGAGCCCACCTTCTCCGCCTTCACTACT
GAAACCTTTGGCAACCCCAACCCCGTGCTCATGTTCAACATCAACTACACCTCCACCTTGGTCA
ACCTCCATCTGGACACCCGAACCGCCTTTTCTTCCCGTGTGGCGCTGGAGGGAGCCGAGGCTG
CAGCAGTCCTTCTCAAGAACGACGGAATTCTGCCTCTGCAGGGCCCCGAGAATGTCGGAGTGT
TTGGAGTCGGCTCACAAATCGGGCCAAAGGGAGCCTACTGCGGGTTCTCCATGCAATGCTCG
GATGGAGCTCTCATCGAGGGATGGGGAAGTGGAACTGCCAACCCAACAGAGTACACTTCTCC
GTACGAAGCACTCCGACAACGAGCCAACCAAGCTGGAGGCCACGTGATTGGAACCACCGAGT
CCTGGAACATGACGCTGCCTCTGATCATGGCTGACAACTCGGACGTTAACATCATCTATGTGCT
GTCCAACTCCGGAGAGGGAGGTTCGACCGTTCTGGACAACATGGGAGACAGAAACAACGTGT
CTCTGTGGCACAATGGCGACGAACTGATCACCACTCTTGCCAACAGCTCCAGAAACAACATTG
TCGTCGTTACCACCGTTGGTCAGGTTGATTTGGAGCCTTGGATCTCGCATCCCAACGTATCGGC
CGTGGTTCTCACTGGTCCTGCTGGTGCCTATGGAGGTAAAGCCATGGCCGAGGTTCTCTATGG
AGACGTCAACCCATCGGGAAAGCTGCCTTATACCATTGCCAAGGACCCCCAAGACTACATTCC
GGTGGTTGTGGACATTCCCGAAGATGGAGCTCCTCAGGCCTACTTTGATGAGGGTGTCTACAT
GGACTACAAGATGTTTGACAAGCTCAACAAGACAGTTCGGTTCGAGTTTGGTTACGGGCTGTC
TTATTCGGATTTCGAGTTTGGAGACCTGGATGTGGTTGTAGACGAGTTCATTTCCGAGCTTCTT
CCTTCTCCTCCTCGGCCTATCATTGTTGACAAGCCCAGCAGCACCAGCTCCAACTCGTCCCACG
GCGATCTCAAGGCTCCCAAGGGTTTCAAGCCCATTCGAGGAGTTGTCTACCCGTGGATTGACG
TTAATTCCCTGACTGCCGGCGAAGCTCTTTCGGGAGTGAGTCCTGCTCTTGCCACTTTGCTGGG
TCTAGGTAAACCTGATACCTGTGCTGTTCCCGCCAAGCACGAGACTGGCGATGATGACAAGG
ACTCCAAGTCTGACGATAAGACCAAGAGAGACACCGAGTCGGATGCTGCCGATGATGAAATT
GAAACAGTCAGCACCTCCAACGAAACCGCCGTTGAAACCACCATTGGAATGAAGAACACCTCC
GAGCCCTTCCACTTTGCCAACGGAACAGGGACGTCAACAAACGCCGCCGGAGGAGTTGGAGG
AAACCCATCTCTCTGGAAAACCGTCGCCACCGTGTCTCACACCATCCGAAACCTCGGTCCCTAT
CCAGGAGCCGCAGTCACCCAGATGTACATTGCCTTCCCTCAGGACGACATTGATTCGCCTCTCA
TTCAACTTCGGGGATTTGACAAGACTCGGGTTCTGGACGTCGGAGAACTCGAAACTAATTCCT

	ACGACATCTTGTGGAGAGACCTGGCTGTTTGGGACGTGGTCATCCAGAGCTGGAGAGTCCAG<br>AGAGGAGAGTACAAGATCTACGTCGGCAACTCCTCCAGAGACTTTGTTCTCATCGAGTCCTTT<br>ACTCTCCAGTAA	
YALI1_E23994p	MEELSEAERNQIGQLFICGLRNAELDEDIAELVRTFKVGSIQVSIKNLPSVDQARELIRGVQQLAYEA<br>GHEQPMAICIDQEGGILNNLKGVTQFPCQMALAASKDTDLVEAVAEAAAKELLACGINFMFAPC<br>MDVLKSTSASADFLLGTRAFGDDHEIVSQFGCAFLRGLQKQNMMACGKHFPGYGTASLDSILGG<br>VPVVSDNELQMQAQAFVPFKAIIDMDCDAIMVGGCSAPGLDPTEAHACLSWKICTDILREQLGFK<br>NVIVSECLEMEALYQQVGVKQGTVSAKLAGCDMILCCSSFKLQKQALEGLSGAYLDGLVEPESIMA<br>SAERVRHMKSSRNLTWQSILGERPFVAELLQEHKLLSARAYESCVAIGRDYLHMIPFSLKRSDTILLL<br>TPLCESDDIHSGRCTPAQTNGNGNPSNVRLLPGEATFQHLGKTIASYHTGKTLHTSYSQNGIIKLHE<br>QLIARATVVILVNTTAYSNMYQTSITKYVQLLCDQRRIPFVLVAASSPHDMASSNHTSDTKTYVCAF<br>EFTPEMQVTCAKVMFGKLPALGTIPFSHFWGRQRSGSKSLSTTSTRKTWMVETYAGENVQPLW<br>DLCFPKRKLPTEAFSVLNDIDCYVVKNSSTGKLYGFCAAHKTQVALLMVDPSRRKMGIAVSLYKRV<br>QRAKGRKDPSAAQLTSFGSVLPAYFAGATPESLAWLQKVDRAMIFRDNVVVLVNQRLQDFRVDA<br>AVIQELQQNNLRFSIASVAETDAKDLPEELRESDAYVAQCLKDGSVVGSVVLFNKGSSIARWLPWI<br>YEFGPSVGGMTSLRCDSDLIMQGLVCCAMRSFRIEGFQTVVLDHMSVAQRDSLSGLGFSVWRVE<br>EKFSR*	This study
YALI1_E23994g	ATGGAGGAATTATCGGAGGCGGAACGAAACCAAATCGGCCAGCTATTCATCTGTGGATTACG<br>TAATGCGGAGCTCGACGAGGACATTGCCGAGCTAGTGCGCACCTTCAAGGTTGGCTCCATCCA<br>GGTGAGCATCAAAAACTTACCTTCGGTGGACCAGGCACGCGAGCTCATCCGCGGCGTGCAAC<br>AGCTGGCCTATGAAGCCGGTCACGAACAGCCCATGGCGATTTGCATCGACCAAGAGGGCGGC<br>ATTCTCAACAACCTGAAAGGCGTGACCCAATTCCCATGCCAAATGGCTCTGGCAGCCTCCAAA<br>GACACCGATCTAGTGGAGGCGGTGGCCGAGGCGGCGGCTAAGGAGCTCCTAGCCTGTGGAA<br>TCAACTTCATGTTTGCACCGTGTATGGACGTGCTCAAGTCGACGTCGGCCAGTGCGGACTTCTT<br>GCTGGGGACCCGTGCCTTTGGAGACGATCACGAAATTGTGTCTCAATTCGGATGTGCTTTCCT<br>TCGTGGACTGCAGAAACAAAACATGATGGCCTGTGGAAAACACTTTCCGGGCTACGGAACAG<br>CCTCGCTGGACTCCATCTTGGGGGGAGTGCCTGTGGTCTCGGATAATGAGCTCCAAATGCAG<br>GCACAGGCGTTTGTGCCGTTTAAGGCGATCATCGATATGGATTGTGATGCCATCATGGTGGGA<br>GGATGTTCTGCTCCAGGTTTGGATCCTACGGAAGCTCATGCTTGTTTGTCCTGGAAGATATGC<br>ACTGATATTCTTAGAGAACAGCTGGGATTCAAGAACGTTATTGTTTCAGAGTGTCTGGAGATG<br>GAGGCTCTTTACCAACAGGTTGGAGTGAAACAAGGAACCGTCTCAGCAAAGCTGGCTGGCTG<br>TGACATGATTTTGTGCTGCTCTTCGTTCAAGTTGCAAAAGCAAGCCCTGGAAGGTCTATCAGG<br>CGCCTATCTCGACGGACTTGTGGAGCCCGAAAGCATCATGGCATCTGCTGAACGCGTTAGACA<br>CATGAAGTCTAGCAGGAATCTCACCTGGCAGAGTATTCTGGGAGAGAGACCGTTTGTGGCAG<br>AGCTGTTGCAAGAACACAAACTGCTGTCTGCACGAGCGTACGAGTCGTGTGTGGCTATTGGC<br>AGAGACTATCTTCACATGATTCCTTTTTCCTTGAAACGCTCAGACACGATTCTACTGCTGACCCC<br>GTTATGTGAATCCGACGACATTCACAGCGGCAGATGCACTCCAGCGCAAACAAACGGCAACG<br>GAAACCCATCCAACGTGCGCTTGCTTCCTGGCGAAGCGACTTTTCAACATCTGGGAAAGACCA<br>TTGCCTCATATCATACTGGAAAGACACTGCACACTTCGTATTCCCAAAACGGAATTATCAAGTT<br>ACACGAACAGCTGATAGCCAGAGCAACGGTTGTGATTCTGGTCAACACAACAGCCTACTCGAA<br>CATGTATCAAACCTCGATAACAAAGTACGTGCAGCTTCTTTGTGACCAAAGACGTATTCCGTTT<br>GTACTGGTGGCTGCTTCAAGCCCCCATGACATGGCGTCCTCCAACCATACATCGGATACAAAG<br>ACGTATGTCTGTGCGTTTGAATTTACCCCAGAAATGCAGGTCACATGTGCAAAGGTCATGTTT<br>GGTAAGCTTCCTGCGCTGGGAACTATCCCATTCTCTCACTTCTGGGGTAGACAAAGGTCTGGT<br>TCCAAGTCACTGAGTACTACGTCTACCAGAAAGACGTGGATGGTGGAAACGTATGCTGGAGA<br>GAACGTGCAACCTCTTTGGGATCTATGTTTCCCGAAACGAAAGCTGCCAACCGAGGCGTTCTC<br>CGTGTTAAATGATATCGACTGCTATGTGGTGAAAAACTCGTCTACTGGAAAGCTGTATGGGTT<br>CTGTGCGGCTCACAAGACCCAGGTTGCACTACTCATGGTGGACCCGTCCAGACGGAAAATGG<br>GCATAGCTGTGAGCCTGTACAAGCGAGTACAAAGAGCCAAGGGCAGAAAGGACCCCTCTGCG<br>GCTCAGCTGACCTCGTTTGGGTCGGTGCTTCCCGCGTACTTTGCTGGTGCTACACCCGAGAGT<br>CTGGCGTGGCTACAGAAGGTGGACCGGGCCATGATCTTTAGAGACAATGTGGTTGTTCTAGT<br>GAACCAGAGGCTGCAGGACTTCCGAGTCGATGCGGCTGTGATCCAGGAGCTGCAGCAGAAC<br>AACCTCCGGTTCTCCATAGCCAGTGTTGCAGAAACAGACGCAAAAGACCTTCCGGAGGAGCT	

	GAGAGAGTCGGATGCCTATGTGGCGCAGTGTCTCAAGGATGGTTCGGTGGTGGGCTCGGTG<br>GTGCTGTTCAATAAGGGTTCTTCCATAGCCCGGTGGCTGCCTTGGATCTATGAGTTTGGTCCGT<br>CGGTGGGTGGCATGACCAGTTTGCGGTGCGACTCTGATTTGATAATGCAAGGGCTTGTTTGCT<br>GTGCTATGAGAAGCTTCCGCATTGAGGGCTTTCAGACGGTGGTATTGGATCACATGAGTGTTG<br>CTCAACGCGACAGTCTTAGTGGGCTTGGCTTTTCTGTCTGGCGGGTGGAAGAGAAGTTCAGCC<br>GGTAA	
YALI1_E40502p	MKFTTLLSMASVAMSAAVTVEIHSTVLETVFETASETIISDAATPEPSPTVRYVTILPKPFPAFNGVA<br>LSGTVHNKRDTVETSVDCNLGLASASAAALREQSSAGTSSAAYTATLTSGSNLSSETHTIEVSSSTSSI<br>PEPATASIETEPNPSLIPLFIPVHQEDSPPDTTTDTHNMGLGTKIRDMVNNVGPVAAADLSKPNQA<br>PNDRTIFQSRNNHGPNIGSMFLLEKWLTPGMFPDSAKGDAELDAVTALVAEQGAGGAQKKFED<br>HWNTWITDDDFSYLQSVGANAIRVPMGYWTINGGAFTQGTPFQQYQSVYQNAWSIFKTNILDK<br>ARAANIAVLIDLHAVPGGANGDAHSGTSSGKVEFWDSRSDQKIAIDALQWVAKDVLSYDNVLGIE<br>VVNEAVYDASTSKEGSYYLRALEAIRQVNPDVPVYISDGWAPTEWNEWVQEQNQKLSAGQNTG<br>FVVDSHVYKAFSEQDKGNSPQQNIANVPAYLNVGKAQADSIVGEFSCVFSEETWAKAGGEDREQ<br>LAIKYGQVQIESFNQNARAGWFFWTYKFQYGDGGDWGFKPMTQKGALPTFSNGGSNKDPKAG<br>IKEAATKATEEHSAYWDQHQGNYEHWRYADGYVSGWQDAAAFYEFHGSTIGRINAWQDARRA<br>QHIAAKGNSGAIWEWDQGFDAGIQGLLNYMRS*	This study
YALI1_E40502g	ATGAAGTTCACAACTCTCCTTTCTATGGCCTCTGTGGCCATGTCTGCCGCCGTCACTGTGGAGA<br>TTCACTCTACGGTGCTAGAAACCGTGTTCGAAACAGCCTCTGAGACGATAATTTCCGACGCCG<br>CAACCCCCGAACCCTCCCCCACAGTCAGATATGTTACCATTCTGCCCAAACCCTTTCCTGCATTC<br>AACGGCGTTGCCCTGAGCGGCACTGTCCACAACAAGCGAGACACCGTCGAGACTTCTGTCGA<br>CTGCAATCTCGGACTGGCCTCTGCCTCTGCTGCTGCTCTCCGGGAGCAAAGTTCAGCTGGTAC<br>CTCGTCCGCAGCATATACCGCAACCTTAACTTCTGGTAGCAATCTTAGCTCCGAGACCCACACC<br>ATTGAGGTTTCTTCTTCTACTTCTTCCATTCCCGAACCTGCCACCGCATCAATTGAGACCGAGCC<br>TAACCCCAGTCTCATCCCCCTCTTTATCCCCGTTCACCAGGAAGACTCCCCCCCCGACACTACCA<br>CCGACACTCACAACATGGGACTTGGAACCAAAATCAGAGACATGGTCAACAACGTTGGCCCC<br>GTGGCCGCTGCCGACCTCAGCAAGCCCAACCAGGCTCCCAATGACCGAACCATCTTCCAGTCG<br>CGAAACAACCATGGCCCCAACATTGGCTCCATGTTCCTTCTCGAGAAGTGGCTGACCCCTGGC<br>ATGTTCCCCGACTCCGCCAAGGGAGACGCTGAGCTCGACGCTGTGACCGCTCTCGTTGCCGAA<br>CAGGGTGCCGGCGGCGCCCAGAAGAAGTTTGAGGACCACTGGAACACCTGGATCACCGACG<br>ACGACTTCTCGTACCTGCAGTCTGTGGGAGCCAACGCCATCCGAGTGCCCATGGGCTACTGGA<br>CCATCAACGGAGGCGCCTTCACCCAGGGCACCCCCTTCCAGCAGTACCAGTCCGTCTACCAGA<br>ACGCCTGGTCCATTTTCAAGACCAACATTCTCGACAAGGCCCGAGCCGCCAACATTGCTGTTCT<br>GATTGATCTGCATGCCGTTCCCGGCGGCGCCAACGGAGACGCTCACTCTGGAACCTCTTCCGG<br>TAAGGTCGAGTTCTGGGACTCTCGAAGCGACCAGAAGATTGCTATTGACGCTCTCCAGTGGGT<br>CGCCAAGGACGTGCTTTCATACGACAACGTTCTCGGTATCGAGGTCGTCAACGAGGCCGTCTA<br>CGACGCTTCCACTTCCAAGGAGGGCTCTTACTACCTGCGAGCTCTCGAGGCCATCCGACAGGT<br>GAACCCTGACGTTCCCGTCTACATCTCCGATGGCTGGGCTCCTACCGAATGGAACGAGTGGGT<br>CCAGGAGCAGAACCAGAAGCTTTCTGCCGGTCAGAACACTGGTTTTGTTGTCGACTCGCACGT<br>CTACAAGGCCTTCTCCGAGCAGGACAAGGGCAACTCTCCCCAGCAGAACATTGCCAACGTTCC<br>CGCCTACCTCAATGTTGGAAAGGCCCAGGCTGATTCTATTGTCGGCGAGTTCTCCTGTGTCTTC<br>TCCGAGGAGACCTGGGCTAAGGCCGGCGGTGAGGACCGAGAGCAGCTTGCCATCAAGTACG<br>GCCAGGTTCAAATTGAGTCCTTCAACCAGAATGCCCGAGCCGGATGGTTCTTCTGGACTTACA<br>AGTTCCAGTACGGCGATGGAGGCGATTGGGGCTTCAAACCCATGACCCAGAAGGGCGCCCTC<br>CCGACCTTCTCCAACGGAGGCTCCAACAAGGATCCCAAGGCCGGCATCAAGGAGGCAGCCAC<br>CAAGGCTACCGAGGAGCACTCTGCCTACTGGGACCAGCATCAGGGTAACTACGAGCACTGGC<br>GATACGCCGATGGATACGTTTCGGGATGGCAGGACGCTGCCGCCTTCTACGAGTTCCACGGCT<br>CCACCATTGGCCGAATCAACGCCTGGCAGGACGCCCGACGAGCCCAGCACATTGCTGCTAAG<br>GGTAACTCCGGTGCCATCTGGGAGTGGGACCAGGGCTTCGACGCTGGTATTCAGGGTCTGCT<br>CAACTACATGCGATCTTAA	
YALI1_F02592p	MFGGGEKKDDQIYKEGRGLFHKSTHSQQTTSNMPIDVQNTLEQLELSEKIALTAGVDIWHTVPIER<br>LGVPSARTSDGPNGIRGTQFFNSEPAACLPASLGLGATWDQDLLYQVGELLAEESRAKSAHVVLA	This study

	PTINIQRSPLGGRSFESFSEDPLLSGKLATQYVKGLQDNKVAACIKHFVTNDQENGRMGSNSVVTD<br>RALREIYLKPFEIAVREANPKAFMTAYNKLNGTHVSEHDILGSVLRGEWKWRGLVMSDWFGTYST<br>SDAVNAGLDLEMPGPPRWRGEQLTHAVLSNKVTTETLDERVTNVLELVKYAQESGIPFNGPESTN<br>NTPKTRALLRKLVADSTVLLKNDANILPLDKTKKVAVIGPNAKATAFCGGGSASLRPYYTVSPFEGIT<br>SKTGSEPDYAVGAYAHKELPDFASYLKSESGDEGIWDVRFYNDKRGAQDRKCFDQLTIEQTKLFLF<br>DYSHKDIPKNKIFYVDATATLKVPKDGVYDFGMTLLGTAKFFVDDKCVLDCTKDQENGNSFFGEG<br>TKEKIGSIELKANKDYAIRLEFGSEATSPRERGGLVSNGGGAVQAGMARQAAAEDTIAEAVELAKR<br>SEQVILFSGLNMAWESEGFDRPNLSLPPHNDALIEAVLDANPNTVIVIQSGAPVEMPWASTAKTIV<br>HATFGGNETGNGIADVLFGDVNPSAKLPITYPLKVQHTPSYYNFGHPKRTLYGEDVFVGYRHYEKV<br>DREVLFPFGHGLSYTSFELSNLSVSKKDDTVTVTVNVKNTGSKTGAEIVQVYVSQEKPSIVRPVKELR<br>GFSKVELDAGKSDTVTVELDVDQATSFWNEYISKWTSEADKYHIHVGTSSAGKHLEGEFEVKKTKN<br>WLGL*
YALI1_F02592g	ATGTTTGGAGGGGGTGAAAAAAAAGACGATCAAATATATAAAGAAGGGCGGGGGCTCTTTC<br>ACAAATCTACACACTCTCAACAAACAACCTCAAACATGCCAATCGACGTGCAAAACACCCTGG<br>AACAGCTCGAGCTGTCGGAGAAAATCGCACTCACCGCTGGAGTCGACATCTGGCATACCGTCC<br>CTATCGAACGGCTTGGCGTACCTTCTGCCCGAACCTCTGATGGCCCCAATGGAATCAGAGGTA<br>CCCAGTTCTTCAACTCCGAGCCCGCAGCCTGTCTTCCGGCCTCCCTAGGCCTCGGTGCTACGTG<br>GGATCAGGATCTCTTGTACCAGGTAGGAGAGCTGCTGGCCGAGGAGTCGCGTGCCAAAAGC<br>GCCCATGTCGTGCTTGCGCCCACAATTAACATCCAGCGGTCCCCTCTAGGAGGTCGCTCCTTCG<br>AGTCTTTCTCTGAAGATCCGCTTTTGTCTGGAAAGCTGGCTACTCAGTACGTCAAGGGTCTACA<br>GGACAACAAGGTGGCAGCTTGTATTAAGCACTTTGTGACCAACGACCAGGAGAATGGCCGCA<br>TGGGATCCAACTCAGTCGTAACAGATCGAGCTTTGCGAGAAATCTATCTCAAGCCCTTTGAGA<br>TCGCAGTCAGAGAAGCCAACCCTAAGGCCTTCATGACCGCCTACAACAAGCTCAATGGCACCC<br>ATGTTAGCGAGCACGACATCTTGGGCAGTGTGCTGCGAGGCGAGTGGAAATGGCGAGGGTT<br>GGTGATGAGTGACTGGTTCGGCACTTACTCCACCAGTGACGCTGTTAATGCCGGTCTGGACCT<br>TGAGATGCCAGGTCCTCCTCGATGGAGAGGCGAGCAGCTCACCCATGCTGTTCTCAGCAACAA<br>GGTGACCACTGAGACGCTTGATGAGCGGGTCACAAATGTTCTTGAGCTCGTGAAGTACGCCC<br>AAGAGTCTGGAATCCCGTTCAATGGTCCCGAATCCACCAACAACACGCCTAAGACTCGGGCTC<br>TGCTACGGAAGCTGGTGGCAGACTCCACTGTCCTGCTCAAAAACGATGCAAACATCTTGCCTC<br>TCGACAAGACCAAGAAAGTGGCCGTGATTGGCCCCAACGCAAAGGCCACTGCTTTCTGTGGA<br>GGTGGTTCAGCCTCTCTCAGACCCTACTATACTGTTTCTCCGTTTGAGGGAATCACTTCCAAGA<br>CTGGCAGCGAACCTGATTACGCCGTAGGAGCCTACGCTCATAAGGAGCTGCCTGACTTCGCAT<br>CATACTTGAAGTCAGAGTCTGGAGACGAGGGAATTTGGGACGTTCGATTCTACAACGACAAG<br>AGGGGTGCTCAGGATCGAAAGTGCTTTGACCAATTGACTATCGAGCAGACCAAACTGTTTCTG<br>TTCGATTACAGTCACAAGGATATCCCCAAGAACAAGATCTTTTATGTGGACGCCACTGCCACCC<br>TCAAGGTTCCTAAGGACGGAGTGTACGACTTTGGAATGACCCTTCTGGGTACTGCCAAGTTCT<br>TTGTCGATGACAAGTGCGTGCTGGACTGCACTAAGGACCAGGAGAACGGCAATTCGTTTTTCG<br>GCGAAGGCACTAAGGAGAAGATTGGGTCTATTGAGCTCAAGGCCAACAAGGACTATGCCATT<br>CGCCTCGAGTTTGGATCCGAGGCCACTTCCCCTCGAGAGCGAGGAGGACTTGTTTCCAACGGT<br>GGAGGCGCTGTTCAGGCCGGCATGGCTCGACAGGCTGCCGCCGAAGATACGATCGCCGAGG<br>CTGTCGAGCTGGCCAAACGTTCCGAACAGGTAATTCTCTTTTCGGGACTCAACATGGCGTGGG<br>AGTCTGAGGGATTCGACCGACCGAACTTGTCGCTTCCTCCTCACAATGATGCACTCATCGAGG<br>CCGTGCTCGACGCGAATCCCAATACCGTTATTGTCATTCAGAGTGGAGCTCCAGTGGAGATGC<br>CCTGGGCATCCACGGCCAAGACCATCGTGCATGCCACCTTTGGAGGCAACGAGACTGGAAAC<br>GGTATTGCCGATGTGCTATTTGGCGACGTGAACCCTAGCGCTAAGCTGCCCATCACCTACCCTC<br>TCAAGGTTCAACATACTCCCTCTTACTACAACTTCGGCCATCCTAAACGAACTTTGTATGGAGA<br>AGATGTGTTTGTGGGCTACCGACACTACGAGAAGGTTGATCGAGAAGTGTTGTTTCCCTTTGG<br>CCATGGTCTTTCATACACTTCATTTGAGCTGTCTAACCTGAGTGTTTCCAAGAAGGATGATACT<br>GTCACTGTAACTGTGAACGTCAAGAACACCGGATCCAAAACGGGAGCTGAAATCGTCCAGGT<br>TTACGTTTCTCAAGAGAAACCATCCATCGTGCGGCCTGTAAAGGAGCTCCGGGGCTTCTCCAA<br>GGTCGAGCTTGATGCTGGCAAGTCCGATACCGTCACTGTGGAACTGGATGTCGATCAGGCCA<br>CCTCATTCTGGAACGAGTATATCAGCAAGTGGACCAGTGAAGCTGACAAGTACCATATTCATG<br>TCGGTACCAGCAGTGCAGGTAAGCATCTTGAGGGTGAGTTTGAAGTCAAGAAGACTAAGAAT<br>TGGTTAGGTCTTTAA

YALI1_F08075p	MKLTKLVALAGAALASPIQLVPREGSFLGFNYGSEKVHGVNLGGWFVLEPFITPSLFEAFGNNDAN<br>VPVDEYHYTAWLGKEEAEKRLTDHWNTWITEYDIKAIAENYKLNLVRIPIGYWAFSLLPNDPYVQG<br>QEAYLDRALGWCRKYGVKAWVDVHGVPGSQNGFDNSGLRDHWDWPNADNVQHSINVINYIA<br>GKYGAPEYNDIVVGIELVNEPLGPAIGMEVIEKYFQEGFWTVRHAGSDTAVVIHDAFQEKNYFNN<br>FMTTEQGFWNVVLDHHQYQVFSPGELARNIDQHIAEVCNVGRQASTEYHWRIFGEWSAALTDC<br>THWLNGVGKGPRLDGSFPGSYYQRSCQGRGDIQTWSEQDKQESRRYVEAQLDAWEHGGDGWI<br>YWTYKTENALEWDFRRLVDNGIFPFPYWDRQFPNQCGF*	This study
YALI1_F08075g	ATGAAACTAACCAAACTTGTAGCTCTGGCAGGAGCCGCTCTGGCTTCGCCTATCCAGCTGGTC<br>CCTCGAGAAGGCTCTTTCCTGGGATTCAACTACGGCAGCGAAAAGGTGCACGGCGTCAACCTT<br>GGCGGCTGGTTTGTGCTGGAGCCCTTCATCACGCCCTCTCTGTTCGAAGCGTTTGGCAACAAC<br>GACGCCAACGTGCCCGTCGACGAGTACCACTATACCGCCTGGCTGGGCAAGGAGGAGGCCG<br>AAAAGCGACTGACGGACCACTGGAACACGTGGATCACCGAGTACGACATCAAGGCCATTGCC<br>GAAAACTACAAGCTCAACCTGGTGCGAATACCCATTGGCTACTGGGCCTTTTCGCTTCTCCCAA<br>ACGACCCCTACGTCCAGGGCCAGGAGGCATACCTCGACCGAGCCCTGGGCTGGTGTAGAAAG<br>TACGGAGTCAAGGCGTGGGTCGATGTCCATGGAGTCCCCGGCTCCCAGAACGGCTTCGACAA<br>CTCCGGACTGCGAGACCACTGGGACTGGCCCAACGCAGACAATGTGCAGCATTCCATCAACGT<br>GATCAACTACATTGCTGGCAAGTACGGCGCTCCCGAGTACAACGACATTGTGGTGGGTATCG<br>AGCTGGTCAACGAGCCTCTGGGCCCCGCCATTGGCATGGAGGTGATTGAGAAGTACTTCCAG<br>GAGGGCTTCTGGACCGTGCGACACGCGGGCTCCGACACTGCCGTGGTCATCCACGACGCCTTT<br>CAGGAGAAGAACTACTTCAACAACTTTATGACCACCGAGCAGGGTTTCTGGAACGTGGTTCTG<br>GACCATCACCAGTACCAGGTCTTTTCTCCCGGCGAGCTGGCCCGGAACATTGACCAGCACATT<br>GCCGAGGTGTGCAACGTGGGTCGACAGGCTTCCACAGAATACCACTGGCGAATCTTTGGCGA<br>GTGGTCCGCTGCTCTGACCGATTGTACCCATTGGCTCAACGGTGTGGGCAAGGGCCCTCGTCT<br>GGACGGCTCCTTCCCCGGCTCGTACTACCAGCGATCGTGCCAGGGTCGAGGCGACATTCAGA<br>CGTGGTCCGAGCAGGACAAGCAGGAGTCTCGACGGTACGTGGAGGCCCAGCTCGATGCGTG<br>GGAGCATGGAGGTGATGGCTGGATCTACTGGACTTACAAGACGGAGAACGCCCTTGAGTGG<br>GACTTCCGACGGCTCGTGGACAATGGCATTTTCCCCTTCCCATACTGGGACCGACAGTTCCCCA<br>ACCAGTGTGGTTTTTAA	
YALI1_F17788p	MFPIGRKGNSQDKGRKRPLLRSHLLPIGTSRDLPDAANTASTGMATATAPSAVASDLSSSMFVRK<br>AVPLRYGTAPNTGVSSVHRSNTVPAHAVSSRAPSVHPDKERSKSAEMAAAVAGIRPTPPPGRRLL<br>PVPAHTPAPVRSHSRVQPQTPRIDFLPQKNPLRRAATDTWDTRTSTQLSTMRSSSFGSVQRTEDD<br>EDNDNDNSNDNDDDGEDSDTTPQPTPIANKYLNTAVPLGDVATAVAPNKNANSNPGFDIKFSSP<br>TFDPKNSSTEKDVEKGEGGEDDADGNESTDDKNEVEMASRTANIHSAQPTFSSNPEYLWELEDF<br>QKRRDERNNNARHLIRKTEDEKRHAERRNKLLLVICCVLAAVLIGITVGMTSTIHRLERSNQKQGLV<br>NQNKQHNDTQRTDGGMLPIGIDTLTDAAKLASILSDTSLHNVLYGIGYSPQNVMTSQCGVNQTE<br>VTLDVAALSRVTKRIRLYGTSCDQALYVLRAIDSLKVDMKVTLGVWINGNPVVCAAQIAEAITAAE<br>QFPHLIESIMVGNEVFFRGELAPAKLVGYMHEVRTELQLRNLTIPVGTSELGSQWSPYMASNVDIL<br>GANIHPFFGGLEVSEATTWTLNFLESFVVNEITEAEKIPQIVISEIGWPSGGGQHGAAKSGVAQQQ<br>RLLQDWVCTAKDLPQIGWYWFEAFDEPWKRVFDTPTEKWESQWGILTPDRRLKKGLSLKISCN*	This study
YALI1_F17788g	ATGTTCCCCATAGGAAGGAAGGGCAATAGCCAGGACAAGGGGCGCAAGAGACCCCTGCTAC<br>GGAGCCACCTGTTGCCCATAGGAACTTCGCGTGATCTCCCCGATGCTGCCAACACGGCCTCCA<br>CCGGCATGGCCACGGCCACGGCGCCGTCTGCGGTCGCTTCCGACCTCTCAAGCTCCATGTTTG<br>TGCGCAAGGCCGTGCCGCTACGCTACGGAACCGCCCCCAACACGGGCGTTTCTAGCGTACAC<br>AGATCCAACACGGTGCCCGCCCATGCCGTCTCCAGCAGAGCGCCATCGGTGCACCCAGACAA<br>GGAGCGCTCCAAGAGCGCAGAGATGGCAGCCGCAGTAGCTGGAATCAGACCTACCCCGCCGC<br>CCGGGCGTCGGCTGCTTCCCGTACCTGCCCACACTCCTGCCCCCGTCCGTTCCCATTCTCGCGT<br>CCAGCCGCAGACACCCCGAATCGACTTTCTGCCACAAAAGAACCCGCTGCGAAGAGCAGCAA<br>CTGATACCTGGGATACCCGCACGTCGACACAACTGTCGACAATGCGCTCGTCGTCGTTCGGGT<br>CGGTGCAAAGGACTGAAGACGACGAAGACAACGACAACGACAACAGCAACGACAACGACGA<br>CGACGGCGAAGACAGCGACACGACGCCCCAGCCCACGCCCATCGCCAACAAGTACCTCAACA<br>CTGCTGTTCCTCTGGGCGACGTGGCAACTGCAGTGGCCCCCAATAAGAATGCAAACTCCAACC	

	CGGGGTTCGACATCAAATTCAGCTCTCCCACGTTCGACCCCAAGAACAGCAGCACCGAAAAGG<br>ACGTGGAAAAGGGCGAGGGTGGAGAAGACGATGCAGATGGCAACGAAAGCACTGACGACA<br>AGAACGAGGTGGAAATGGCCTCTCGCACTGCCAACATCCACTCCGCACAGCCCACCTTTAGCT<br>CCAACCCCGAGTACTTGTGGGAGCTCGAAGACTTTCAGAAACGACGAGACGAGCGCAACAAC<br>AACGCCAGACACCTGATACGAAAGACAGAGGACGAGAAGCGACATGCCGAACGACGCAACA<br>AACTCCTACTAGTTATTTGTTGCGTGCTTGCAGCGGTGCTGATTGGCATCACTGTAGGTATGAC<br>TTCGACCATCCATCGGCTTGAAAGATCAAATCAAAAACAAGGACTGGTCAACCAGAACAAACA<br>GCACAACGATACCCAAAGAACCGACGGCGGCATGTTACCCATTGGAATCGATACCCTGACAG<br>ATGCAGCCAAGCTCGCAAGCATTCTGTCGGATACCTCTTTGCACAACGTTCTCTACGGCATTGG<br>CTACTCGCCCCAGAACGTCATGACATCCCAATGTGGAGTCAACCAGACGGAAGTGACCTTGGA<br>TGTTGCTGCATTGTCTCGTGTTACAAAGCGGATCCGACTTTACGGCACCAGCTGTGACCAAGC<br>CCTGTACGTTTTACGGGCGATTGATTCCCTCAAGGTAGATATGAAAGTGACTCTCGGAGTGTG<br>GATCAACGGCAACCCTGTAGTTTGCGCTGCGCAGATTGCTGAAGCAATCACTGCTGCTGAGCA<br>GTTCCCCCATTTGATTGAATCCATCATGGTGGGTAATGAGGTGTTTTTCCGCGGCGAGCTCGC<br>GCCAGCCAAACTTGTGGGCTACATGCATGAAGTGCGGACGGAGCTACAGCTTCGCAACCTCA<br>CAATCCCCGTGGGCACCTCCGAGCTGGGCTCTCAATGGTCTCCGTACATGGCCTCGAATGTGG<br>ATATTCTAGGAGCCAACATCCATCCATTCTTTGGAGGCCTTGAAGTGTCTGAGGCGACCACCT<br>GGACACTCAATTTCCTCGAGTCATTTGTCGTCAACGAGATTACAGAAGCCGAAAAAATACCCC<br>AAATTGTGATATCCGAGATTGGATGGCCCAGTGGAGGTGGTCAACACGGCGCTGCAAAGTCG<br>GGTGTAGCGCAACAGCAGCGACTCCTACAAGACTGGGTGTGCACGGCCAAGGACTTGCCGCA<br>GATTGGATGGTACTGGTTCGAGGCGTTTGACGAGCCGTGGAAGCGTGTGTTTGACACCCCGA<br>CTGAAAAGTGGGAGTCTCAGTGGGGTATTTTGACCCCTGACCGGCGTCTAAAAAAGGGGTTG<br>TCGCTGAAAATCAGCTGTAATTAA	
YALI1_F21504p	MIFSLQLLLTTALAASSPDWYPTPEIGSITSDWADALGESMDILAQLTLPEKVNITTGTGWRGGQC<br>VGNTGAVPRLGIKGLCLQDGPLGVRFADFVNVFPCQNAMAATFDRILVHQRGTAIGRQSRLKGV<br>DVHLGPVVGPLGRHATGGRNWEGFSPDPYLSGKLAFEAILGVQEEGVLATIKHFIGNEQEHYRRA<br>EEWRDGFGFKDLKDAVSSNIEDRALHELYMWPFADAVRANVGSVMCSYNYVNGTQACQNSDLL<br>NGKLKSELGFQGFVMSDWFAQGSGVSNALAGMDMSMPGNDVDELETVFWGEQLTRMVANG<br>TLPEARLDDMVLRILTPLIYFGIDDRTPNFSSFVDTTVGSPHPAAKHSKKVKDVITNYHLDVRDQFA<br>ANVALDSARGAVVLLFNDGILPLKNISAIGVFGVGSRLGPNGAVCGENMQCSDGALIEGWGSGT<br>AYPTEYESPYEALHKKASLLEVSVTGTTESWDMRLPLELAGDTDVNIVYVLANSGESTANVDKNLG<br>DRRNVSLWHNGDELINTVASQGQTVVVVTTVGQVDMTAWLNHPNISAVLLTAPAGDYGGKAIA<br>DVLFGEVNPSGKLPYTIAANTSDYIPIVTKIPRDGAPQSDFVEGIYLDYKWYDKFERTPLYEFGYGLS<br>YTTYSFSNLHLDVKEISEFLPPRPVPVQVTKPKMTNIDIEDLYVPNDFKMIDGLVYPWILNASAPLA<br>DSQTQFPFANGAGHVSDASGGVGGHPWLWSNAVTVTHNTTNCGDVAGRVVSQLYVAFPETLI<br>DSPPVQLRGFDKSKLLNPGESQMTEYNLNWRDLAIWDVELQSWRVQRGEYSVYIGHSSREFELCE<br>TFTL*	This study
YALI1_F21504g	ATGATCTTCTCTCTGCAACTACTACTGACGACGGCACTAGCGGCCTCTTCGCCTGACTGGTACC<br>CCACACCCGAAATCGGTTCAATTACCAGTGATTGGGCCGATGCTCTTGGTGAGTCCATGGACA<br>TCCTGGCCCAATTGACTCTTCCCGAAAAGGTCAACATCACCACCGGTACCGGATGGAGAGGTG<br>GGCAGTGTGTTGGAAACACAGGGGCTGTTCCTCGTCTCGGAATCAAGGGCCTGTGTCTCCAA<br>GACGGTCCTCTGGGCGTCCGTTTTGCCGACTTTGTCAATGTCTTCCCTTGCCAGAACGCAATGG<br>CCGCCACCTTTGATCGTATCCTAGTTCACCAACGAGGAACCGCTATTGGACGTCAGTCTAGACT<br>CAAGGGAGTCGATGTTCATCTCGGACCAGTGGTGGGACCTCTTGGACGACACGCTACCGGCG<br>GAAGAAACTGGGAAGGTTTCTCCCCCGACCCATATCTGTCTGGAAAGCTCGCCTTCGAAGCAA<br>TTCTTGGAGTCCAGGAAGAGGGAGTTCTTGCAACCATCAAGCACTTCATTGGAAACGAACAAG<br>AACATTACCGGCGAGCCGAAGAGTGGAGAGACGGCTTTGGATTTAAAGACCTGAAGGACGCC<br>GTCTCTTCCAACATTGAAGACAGAGCTCTACATGAGTTGTACATGTGGCCGTTTGCCGATGCT<br>GTTAGGGCTAATGTCGGCTCAGTCATGTGCTCCTACAACTACGTGAACGGAACCCAGGCTTGC<br>CAGAACAGTGACTTGCTCAACGGAAAGCTCAAGTCCGAGCTCGGTTTCCAGGGCTTTGTCATG<br>TCCGACTGGTTTGCTCAGGGAAGCGGAGTGTCTAACGCTCTGGCTGGAATGGACATGAGTAT<br>GCCTGGAAATGACGTTGATGAGTTAGAAACTGTCTTCTGGGGAGAACAGCTGACCCGAATGG<br>TTGCCAACGGTACCCTTCCAGAAGCCCGTCTCGATGATATGGTTCTGCGAATTCTGACCCCTCT	

	CATCTACTTTGGGATCGACGATCGAACACCCAACTTCTCCTCCTTTGTCGACACTACAGTGGGA<br>AGTCCCCACCCCGCTGCTAAGCACTCCAAGAAAGTCAAGGATGTTATCACCAACTACCATCTCG<br>ATGTGCGAGACCAGTTTGCAGCCAATGTTGCTCTTGATAGTGCTCGAGGAGCTGTTGTTCTGC<br>TTTTCAATGACGGTATTCTTCCCCTGAAGAACATTTCCGCTATTGGAGTATTTGGAGTCGGCTC<br>TAGACTTGGCCCCAATGGAGCTGTTTGTGGGGAAAACATGCAGTGCTCAGACGGAGCTCTTAT<br>CGAAGGATGGGGAAGTGGAACTGCTTACCCTACGGAGTACGAAAGCCCTTACGAAGCTCTCC<br>ACAAAAAGGCTTCTCTACTTGAAGTGTCAGTGACAGGAACCACCGAGTCATGGGATATGAGA<br>CTTCCTCTTGAGCTGGCTGGAGACACTGACGTGAATATTGTTTATGTATTGGCAAATTCAGGA<br>GAGTCTACTGCCAACGTTGATAAGAACCTTGGGGATCGCCGAAATGTCAGTCTATGGCACAAT<br>GGCGATGAACTTATTAATACAGTTGCCAGTCAGGGACAGACTGTCGTTGTTGTCACTACGGTT<br>GGACAAGTTGACATGACCGCTTGGCTCAACCACCCCAACATCAGTGCCGTTCTTCTGACTGCTC<br>CTGCTGGTGATTACGGAGGAAAAGCCATCGCTGATGTGTTATTTGGAGAGGTTAATCCCTCAG<br>GAAAGCTGCCTTATACTATCGCAGCAAATACTTCTGATTATATTCCTATTGTCACCAAGATCCCT<br>CGAGATGGAGCTCCCCAGTCCGACTTTGTGGAGGGAATCTATCTTGACTACAAGTGGTACGAC<br>AAGTTTGAAAGGACTCCCCTCTACGAATTTGGTTACGGTCTGTCATACACCACCTACTCCTTCA<br>GCAATCTGCATCTTGATGTTAAGGAGATTAGTGAGTTCCTTCCTCCCCGGCCTGTTCCTGTACA<br>GGTTACTAAACCCAAGATGACCAATATCGACATTGAGGACCTTTATGTCCCCAATGACTTTAAA<br>ATGATTGACGGTCTTGTTTACCCTTGGATTCTCAACGCCAGTGCGCCCCTCGCGGACTCTCAGA<br>CTCAGTTCCCCTTTGCAAATGGAGCTGGACATGTCAGTGACGCTTCTGGAGGAGTGGGTGGTC<br>ACCCCTGGCTTTGGTCTAACGCTGTTACTGTCACTCACAACACCACCAACTGCGGTGATGTTGC<br>TGGACGAGTAGTTTCTCAGCTGTACGTTGCCTTCCCTGAAACCCTTATCGACTCTCCTCCGGTG<br>CAGCTTCGAGGATTCGACAAGTCCAAGCTCCTGAACCCTGGTGAGTCTCAGATGACCGAGTAC<br>AACCTCAACTGGCGAGATCTGGCCATTTGGGACGTGGAACTGCAGAGCTGGAGAGTGCAGCG<br>AGGCGAGTATTCCGTGTACATTGGTCATTCCAGCCGAGAATTCGAGCTATGTGAGACTTTCAC<br>TTTGTAA	
YALI1_E25163p	MPLLPPSCPPAPQTTSDAAPSPQPAPTTFSTSVIEPSSTSSSAAEATPTFTPNGILPYSLTYSPYNDDS<br>SCKTMDEVMKDLKEIVAKGIKVIRVYGTDCGSVQTIEPAAKQLGLKINQGFWIGPDGVDSIDSGVQ<br>EFINWVQQNQAWGMIDSITVGNEAIIAGYVSPQQLLGKIGQVKSQLKAAGYQGQVTTAEPAVSY<br>TTHPELCTGPELDYVGINSHAYFNPQQSPETAGQFALDEMALTQKTCNNKVVFVTETGYPSAGNT<br>NGNNVPTPQNQEIAINSLLKALNGYGTFFTMYNDFWKAPGPYNVEQHFGIINILQ*	This study
YALI1_E25163g	ATGCCCCTCCTGCCCCCTTCCTGCCCTCCTGCCCCTCAGACTACCTCTGACGCTGCCCCCTCTCC<br>CCAGCCTGCCCCTACCACCTTTTCCACTTCTGTTATCGAGCCCTCTTCCACCTCTTCTTCTGCTGC<br>GGAGGCCACTCCCACTTTCACGCCCAACGGAATTCTGCCCTACTCGCTCACCTACTCGCCCTAC<br>AACGACGACTCGTCCTGCAAGACTATGGACGAGGTCATGAAGGACCTCAAGGAGATCGTCGC<br>CAAGGGTATCAAGGTGATCCGTGTCTACGGCACTGACTGCGGCTCTGTCCAGACCATTGAACC<br>TGCCGCTAAGCAGCTTGGTCTCAAGATCAACCAGGGTTTCTGGATCGGACCCGACGGAGTTG<br>ACTCCATTGACTCTGGTGTCCAGGAGTTCATTAACTGGGTCCAGCAGAACCAGGCCTGGGGTA<br>TGATTGACTCTATCACTGTTGGAAATGAGGCTATCATTGCCGGCTACGTCAGCCCCCAGCAACT<br>CCTTGGAAAGATCGGCCAGGTCAAGAGTCAGCTCAAGGCTGCTGGATACCAGGGACAGGTCA<br>CCACCGCCGAGCCTGCTGTCTCCTACACCACCCACCCCGAGCTGTGCACTGGCCCTGAGCTCG<br>ACTACGTTGGGATCAACTCTCACGCCTACTTCAACCCCCAACAGTCTCCTGAGACCGCCGGCCA<br>GTTTGCTCTGGACGAGATGGCTCTTACTCAGAAGACTTGTAACAACAAGGTTGTTTTTGTCACC<br>GAGACAGGCTACCCTTCTGCTGGTAACACCAACGGCAACAATGTTCCTACTCCCCAGAACCAG<br>GAAATTGCCATCAACTCTTTGCTCAAGGCCCTCAATGGCTACGGCACCTTTTTCACCATGTACA<br>ACGACTTCTGGAAGGCCCCCGGTCCTTACAACGTTGAGCAGCATTTTGGTATCATTAACATTCT<br>TCAGTAA	
YALI1_E39796p	MKFSTLALAAMAAIGFAAPATPDADCNEEKEVRDFHAHHQHKRAVQVEYVYVTVLVDGNGNTIE<br>QQQIATTELQEAPSPAPATSTQEAAPAPSSTLVAAFAAKEPVTQEASSAASSAAPSSTSAPKSSSAP<br>SKPSNGGSGGITGDLAAFSGPSEKFQDGTIDCGDFPSGQGVIALNQLGFGGWSGIYHPGSTATGG<br>NCAEGAYCSYACQSGMSKTQWPDEQPANGVSVGGLLCKGGKLYRSNQQSDYLCEWGTQSAIVT<br>SSLDKEVAICRTDYPGTENMVIPTVVEAGSSGVPLAVVNQDAYYTWMGKPTSAQYYVNNAGVSY	This study

	QDGCLWGTSSGDVGNWAPLNFGAGAAGGISYLSIIPNPNNRNSANFNVEIIATNGATVNGKCVY<br>ENGKYNGGDSNGCTVAVTGGVAAFHLY*	
YALI1_E39796g	ATGAAGTTTTCCACTCTTGCTCTGGCCGCAATGGCCGCCATTGGCTTTGCCGCCCCCGCCACTC<br>CCGACGCTGACTGCAACGAGGAGAAGGAGGTCCGAGACTTCCACGCTCACCACCAGCACAAG<br>CGAGCTGTGCAGGTCGAGTACGTCTACGTCACTGTCCTTGTTGACGGTAACGGAAACACCATT<br>GAGCAGCAGCAGATTGCCACCACCGAGCTCCAGGAGGCTCCCTCCCCTGCTCCCGCTACCTCC<br>ACCCAGGAGGCTGCTCCCGCTCCTTCCAGCACCCTTGTTGCTGCTTTTGCCGCCAAGGAGCCCG<br>TCACCCAGGAGGCTTCTTCTGCCGCTTCTTCCGCCGCTCCTTCCTCCACCTCTGCCCCTAAGTCT<br>TCTTCTGCTCCTTCCAAGCCTTCCAACGGAGGCTCCGGAGGCATCACTGGTGACCTCGCTGCCT<br>TCTCTGGTCCTTCCGAGAAGTTCCAGGACGGTACCATTGACTGTGGAGATTTCCCCTCCGGCCA<br>GGGTGTCATTGCCCTGAACCAGCTTGGCTTCGGCGGCTGGTCCGGTATCTACCACCCCGGTTC<br>CACTGCCACCGGTGGCAACTGTGCCGAGGGTGCTTACTGCTCTTACGCTTGCCAGTCTGGTAT<br>GTCCAAGACCCAGTGGCCTGATGAGCAGCCCGCCAACGGTGTTTCCGTTGGTGGTCTTCTCTG<br>CAAGGGCGGTAAGCTTTACCGATCCAACCAGCAGTCTGACTACCTCTGCGAGTGGGGCACCC<br>AGTCCGCCATTGTGACCTCTTCTCTCGATAAGGAGGTTGCTATCTGCCGAACCGATTACCCCGG<br>TACCGAGAACATGGTTATCCCCACTGTCGTTGAGGCCGGTTCCTCCGGTGTTCCTCTTGCCGTC<br>GTCAACCAGGATGCTTACTACACCTGGATGGGCAAGCCTACCTCCGCTCAGTACTACGTCAAC<br>AACGCTGGTGTTTCTTACCAGGATGGCTGTCTCTGGGGTACTTCTTCCGGTGATGTCGGTAACT<br>GGGCTCCTCTGAACTTCGGTGCTGGCGCTGCCGGCGGTATCTCTTACCTTTCCATCATTCCTAA<br>CCCCAACAACCGAAACTCCGCTAACTTCAACGTTGAGATCATCGCTACTAACGGTGCTACTGTC<br>AACGGTAAGTGTGTCTACGAGAACGGTAAGTACAACGGTGGTGACTCCAACGGTTGCACTGT<br>TGCTGTTACCGGCGGTGTTGCTGCCTTCCACCTTTACTAA	
YALI1_F03075p	MMSYQSTKAPPPFRRTPTKSPVRSSFESVSSNTPLDFSLPSYHSPSEKLQPPQKGSKSKRYVLVLAT<br>AALCCFMYILAMSAPEKTGSDALVALKNKVSQSTETLKQLIPAQPFVWSEPTPNFHSYAIAYLPLNK<br>NSKCRRQSQITADFASLEASGRVEAIRLFSTDCKVIPAYLDYLKNRDSKKRSVDSALKLVLGIEPSALE<br>TTSDLDSEATSLELLTRSVDAQLMDIIDSLDESSSATEAFLRQLDLIVLGSEGLANQNYHPKELVEVLA<br>HIRQRLDTLSSDIRIPVTTSEPLGIWDVLTPKERRDNVQEMLARQDGVHSVTPDDIEGPTGLFCDSV<br>DVIGLSVSPFNNPTVDAAHAGAEVEESAYLASLLCNKPVLALEVGWPSAGSDNGNAKTGKDEQLQ<br>AVSSITAAVERISGKPLRSVLYQYFDDEWLSADEDFARHFGLKRLF*	This study
YALI1_F03075g	ATGATGTCTTACCAATCGACCAAGGCCCCACCGCCTTTTCGACGGACCCCTACAAAGTCGCCG<br>GTACGATCCTCGTTTGAGTCAGTGTCTTCGAATACCCCACTGGACTTTTCTCTGCCGTCATACCA<br>CTCGCCTTCGGAGAAGCTCCAACCACCACAGAAGGGATCCAAGAGCAAACGATATGTCCTTGT<br>TCTGGCCACAGCAGCACTATGTTGCTTCATGTACATACTGGCCATGTCTGCGCCTGAAAAGACT<br>GGAAGTGATGCTCTGGTGGCCCTCAAGAACAAGGTCTCGCAGTCAACAGAGACTCTCAAGCA<br>GCTTATCCCCGCCCAGCCGTTCGTATGGTCCGAACCCACACCCAACTTCCACAGCTACGCCATC<br>GCCTACCTGCCACTCAACAAGAACAGCAAGTGCCGCCGACAGAGCCAGATCACTGCCGACTTT<br>GCATCTCTGGAGGCTTCAGGCCGCGTAGAGGCCATCCGGCTCTTTTCTACCGACTGCAAGGTC<br>ATTCCGGCCTATCTGGACTACCTCAAGAATAGAGACTCGAAGAAGCGAAGCGTGGACTCCGCT<br>CTCAAGCTAGTACTTGGAATCGAGCCCAGTGCACTGGAAACCACTTCGGATCTGGACTCCGAG<br>GCCACGTCTCTCGAGCTGCTGACCAGATCTGTCGACGCCCAGCTCATGGACATCATCGACTCTC<br>TGGACGAGTCTTCTTCTGCCACCGAGGCCTTTCTTCGACAGCTGGATCTGATCGTGTTGGGTTC<br>CGAGGGCCTGGCCAACCAAAATTACCATCCCAAGGAGCTCGTCGAGGTCCTGGCTCACATCCG<br>ACAGCGTCTCGACACTCTGTCTTCCGACATTCGAATCCCAGTCACCACCTCCGAGCCTCTGGGC<br>ATCTGGGACGTGCTGACACCCAAGGAGCGACGAGACAACGTCCAGGAAATGCTTGCACGACA<br>GGACGGAGTGCACTCTGTCACTCCCGACGACATTGAAGGCCCCACGGGTCTGTTTTGCGACTC<br>CGTCGATGTGATTGGTTTGTCTGTCTCTCCCTTCAACAACCCTACTGTCGACGCTGCCCATGCC<br>GGTGCCGAGGTCGAGGAGTCTGCCTACCTTGCCTCGCTTCTGTGTAACAAGCCTGTGCTCGCC<br>CTGGAGGTGGGATGGCCCTCGGCTGGTTCGGATAACGGCAACGCCAAGACCGGCAAGGACG<br>AACAGCTACAAGCCGTGTCTAGTATCACTGCTGCTGTTGAGCGAATCTCCGGCAAGCCCCTGA<br>GATCCGTTTTGTATCAGTACTTTGACGACGAGTGGCTGTCCGCAGACGAGGACTTTGCCCGCC<br>ATTTCGGCCTGAAGCGGTTGTTCTAA	

2. Overview of the sustainability assessment methodology applied to the betanin production

A stepwise sustainability assessment methodology was applied to estimate the environmental and sustainability performance of a new fermentation-based betanin production. The methodology combines experimental data, process model, market analysis, Life Cycle Assessment (LCA), and Techno-economic Assessment (TEA) into a single framework. The supporting data on this methodology is described below in this supplementary file. The overview of the methodology is presented in Figure S1.

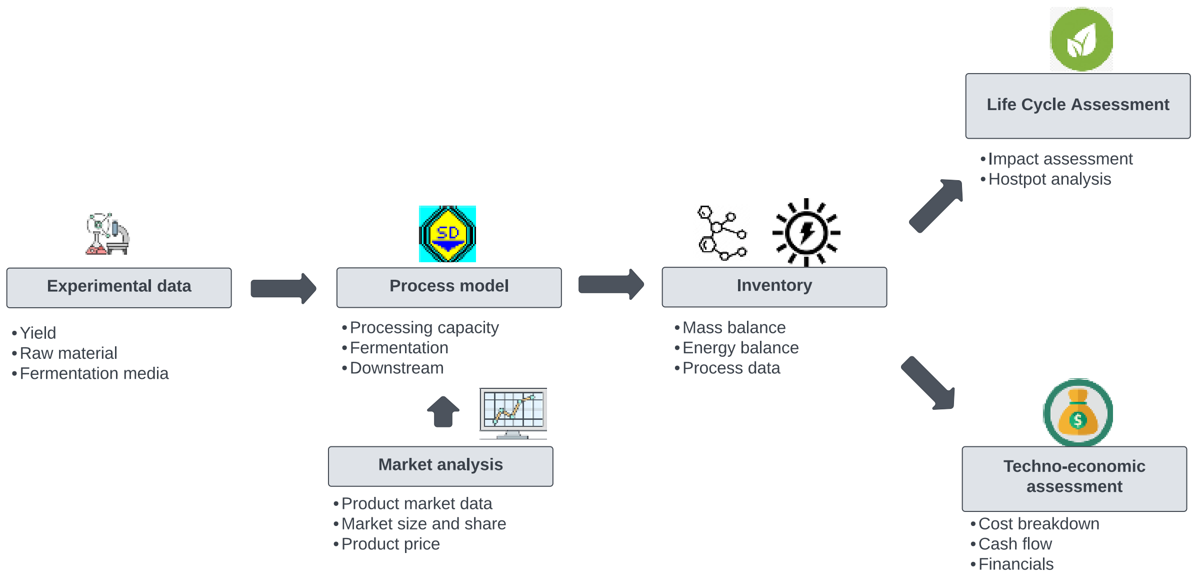

Supplementary Figure S1. Methodology applied for the sustainability assessment of betanin-based colorant

3. Market data and analysis of betanin production

Betanin is a red colorant extracted from beetroot, and it is often commercialized as the food additive E162 with a betanin concentration ranging from 0.4% to 1.2%[8]. The global production volume of colorant E162 is unknown, and so is the current demand. We had to proceed with an estimate of the global capacity of E162 production, starting from the global food colorant market size[9,10], reported at 1,75 Billion USD (2022 estimate). Assuming optimistically that the betalains cover around the same market share of the carotenoids (30% of the total volume[10]), we calculated the expected market share as in Table S3.

Supplementary Table S3. Summary of market data, estimated from the aggregated food colorant market.

Aspect	Value	Unit
**Total food colorant market**	1,75	Billion USD
**Assumed market share betalains**	30	%

**Threshold betalains market**	525	M USD

As the next step, we investigated the price of E162 on the market. For the TEA, it was assumed that the E162 manufactured in the production plant would be produced and sold to other businesses. Both business-to-customer (B2C or retail) and business-to-business (B2B or wholesale) prices were found. The price varies greatly depending on the quantity purchased. For wholesale purchases, and a traded quantity of 25 kg or more, prices range from 8,5 to 25 USD/kg, as reported in Table S4. This is more representative of a B2B case, but since very little data was found, retail prices were also investigated. For retail purchases, the range is higher but to make the prices comparable, it was assumed that the corresponding B2B price is 33% of the prices found, reported in Table S5. This yields to higher estimated B2B prices, ranging from 20,4 to 61,3 USD/kg. In this analysis, aspects related to the traded amounts were not considered and only the normalized prices were used.

Supplementary Table S4. Wholesale price of E162, or "B2B" price.

Traded amount [kg]	Normalized wholesale price [USD/kg]
25	8,5[11]
25	15[12]
200	25

Supplementary Table S5. Retail price of E162, or “B2C”, and the calculated B2B price assumed as one third of B2C.

Traded amount [kg]	Normalized retail price [USD/kg]	Calculated B2B price [USD/kg]
0.50	61.10[13]	20.40
0.50	184.00[14]	61.30
1.00	172.20[14]	57.40
2.00	138.20[15]	46.10
10.00	99.50[16]	33.20
5.00	132.70[17]	44.20

The range for the market price assumed for the analysis is therefore ranging from 8.5 to 61 USD/kg of E162 colorant (the numbers were rounded off). Three prices were analyzed: one for the pessimistic scenario, one for the baseline scenario, and one for the optimistic scenario, shown in Table S6. The baseline scenario price is the arithmetic average of the upper and lower boundaries. From here, the market volume upper boundaries were calculated according to Eq (s1).

$$Market\ volume\ \left[\frac{t}{y}\right] = Market\ size\ \left[\frac{USD}{y}\right] \times Price\ \left[\frac{USD}{t}\right] \qquad Eq\ (s1)$$

Following this baseline, a target market share for the company was selected. Since fermentative production constitutes an innovation in E162 manufacturing, and more in general in the food colorant market, a conservative approach suggested keeping the target market share between 1 to

5%, assuming one single factory to be built to penetrate the market. This leads to the plant capacity boundaries calculated using Eq (s2).

$$Plant\ capacity\ \left[\frac{t}{y}\right] = \ Market\ volume\ \left[\frac{t}{y}\right] \times Target\ market\ share\ [\%]\quad Eq\ (s2)$$

The results, summarized in Table S6, were used to evaluate the sensitivity of the Pay Back Period at different plant capacities and prices. It must be emphasized that the pessimistic scenario (8.5 USD/kg) did not converge for any of the plant capacities selected; therefore, it was necessary to recalculate the minimum acceptable selling price. The new minimum selling price was recalculated by targeting a Pay Back Period of 3 years in the glucose scenario. This led to a recalculated pessimistic price of 21,5 USD/kg. The plant capacities were kept the same as the previous pessimistic selling price.

Supplementary Table S6. B2B prices, market volume boundaries and upper and lower plant capacities assumed for the analysis.

Scenario	Selling price [USD/kg]	Market volume boundaries [t/y]	Capacity at 1% market share [t/y]	Capacity at 5% market share [t/y]
Optimistic	61.00	8607	86	430
Baseline	34.75	15108	151	755
Pessimistic	8.50	61765	618	3088
Pessimistic, recalculated	21.50	61765	618	3088

Unfortunately, there is no specific data to give an educated guess on the real market size. However, it seems reasonable to represent a share of up to 30% of the Natural Colorants market size, given that the carotenoids only represent a market share of 30%. Hence, 525 USD million is the market size range that appears realistic. In the context of trade analysis, it was possible to estimate food colorants and dyes only at a general level since there is a lack of disaggregated data. The HS code used for trade analysis is HS320300 (Description: *Coloring matter of vegetable/animal origin (incl. dyeing extracts. excl. animal black),* whether/not chemically defined*.* Figure S2 shows the aggregated amounts of Imports & Exports at a global level in terms of value and weight.

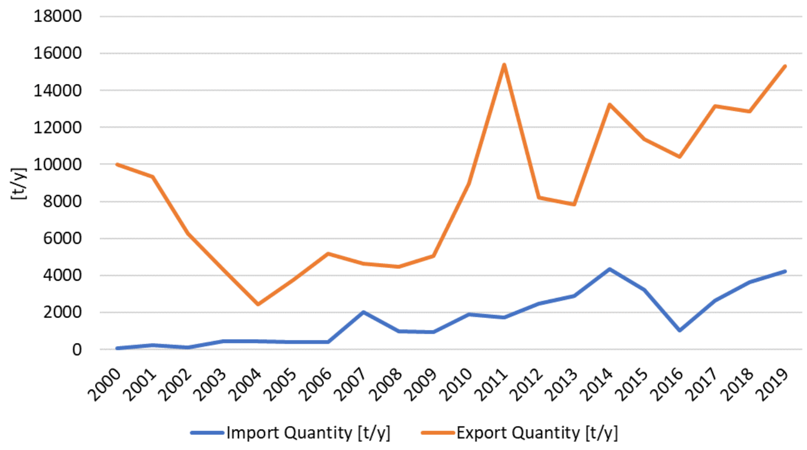

(a)

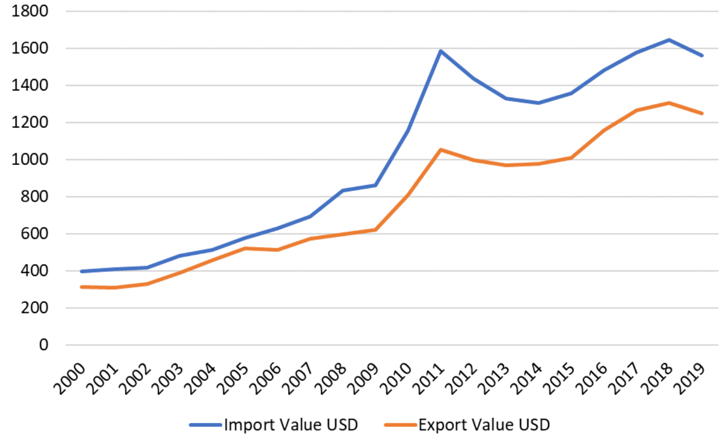

(b)

Supplementary Figure S2. Global Import-export quantities in t/y (a) and Value in Million USD (b) from 2000 to 2019.

The graphs above suggest that the global trade of natural colorants has grown in the last 20 years, mirroring the growing consumers and industry shift to natural food solutions. The time series has been tested for "seasonality" to infer future growth with negative results. Therefore, the lack of seasonality in the series prevents the creation of statistically significant results regarding forecasts. Table S7 shows the top exporter and importer countries in 2018.

Supplementary Table S7. Top Exporter and Importer countries in terms of value in year 2018

Exporter	Value (USD Million)	Importer	Value (USD Million)
**China**	198.27	USA	198.03
**USA**	123.68	Japan	137.63
**Netherlands**	123.45	Germany	107.35
**Denmark**	110.71	Spain	91.52
**Spain**	100.40	UK	75.28

4. Techno-economic assessment supporting data

4.1. Description of the extraction-based and fermentation-based betanin production

The TEA methodology involved the analysis and simulation of both traditional betanin production and innovative production through fermentation. The analysis of the extraction-based process was fundamental for benchmarking the fermentation-based process. The scope of analyzing such technology was to provide fact-based inventory to the LCA and compare the fermentation-based products on the same capacity scales. It should be remembered that the traditional process, being a highly mature technology, provides more data-driven results for the fermentation-based process, built upon low-maturity information and several assumptions.

As described, the extraction-based process serves as a reference to set up the calculations and process design for the fermentation-based process. It is recognized in the current industry that one of the major problems related to colorant production is the purification step. The betanin purification methods in the literature include chromatography and aqueous two-phase systems extraction. Therefore, the modeling of the downstream process for the fermentation-produced betanin was based on the current traditional method, adopting the steps which occur after the enzymatic digestion step, for which data is available in Wiley[18] and von Elbe and Amundson[19], and recently confirmed by the European Food Safety Authority (EFSA)[8]. Moreover, it is assumed that recombinant DNA techniques can achieve a high degree of selectivity during the production of betanin, resulting in a fermentation broth with few impurities, and no toxic compounds present at the time of harvest.

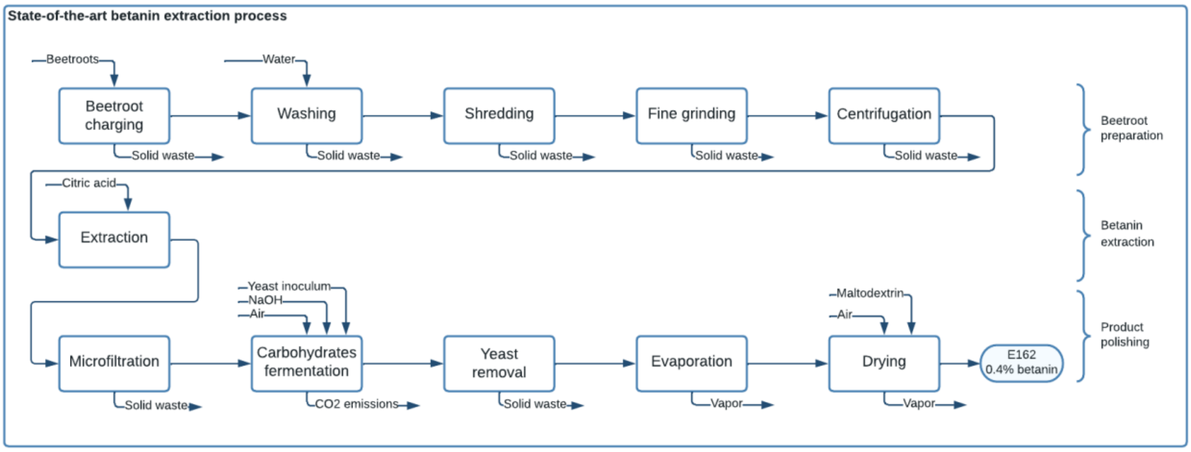

Supplementary Figure S3. Block Flow Diagram of the traditional betanin production process. Minor streams, utilities and CIP, SIP waste is not reported.

4.2. Process description – extraction process

The beetroots are charged and transported by a conveyor belt for washing to remove dirt and any eventual residues from the fields. They are then shredded and ground into a pulp. The bulk solids (fibers) are removed through a basket centrifuge to collect the pulp, rich in betalains. The betanin is later extracted in a continuous extractor (simulated using a stirred tank) with a citric acid solution. The juice containing extracted betanin is filtered in a microfiltration unit and then processed into a fermenter to remove the soluble carbohydrates, nitrates, nitrites, and proteins. To achieve this, the yeast, *Candida utilis*, is inoculated into the reactor and fermented for several days until the carbohydrates and proteins are converted into insoluble products. After fermentation, the yeast and the insoluble products are removed with a basket centrifuge and then concentrated into a thin film evaporator. The concentrated juice is sprayed into a carrier agent in a spray dryer. The carrier agent used is maltodextrin (powder form)[20]. The product is finally formulated into a powder with a net content of betanin of 0.4%, following the minimum content indicated by the EU standard for the E162 food colorant[8]. The extraction process was neither optimized nor subject to sensitivity analysis since it was assumed that the process is the current one used in the industry, and for the same reason, a TEA was not necessary to be performed other than for the scope to generate an inventory for the LCA. Moreover, it is mentioned by EFSA[8] that this is still the technology in use, up to date. The design parameters of the equipment are found in Table S5, while the design specifications are in Table S6.

4.3. Process description – fermentation-based betanin production

The fermentation process comprises two steps, the upstream and downstream. The upstream was modeled according to the experimental conditions used in the laboratory, where available. In contrast, as mentioned, the downstream is modeled using the downstream steps described in the extraction process after the fermentation of the carbohydrates.

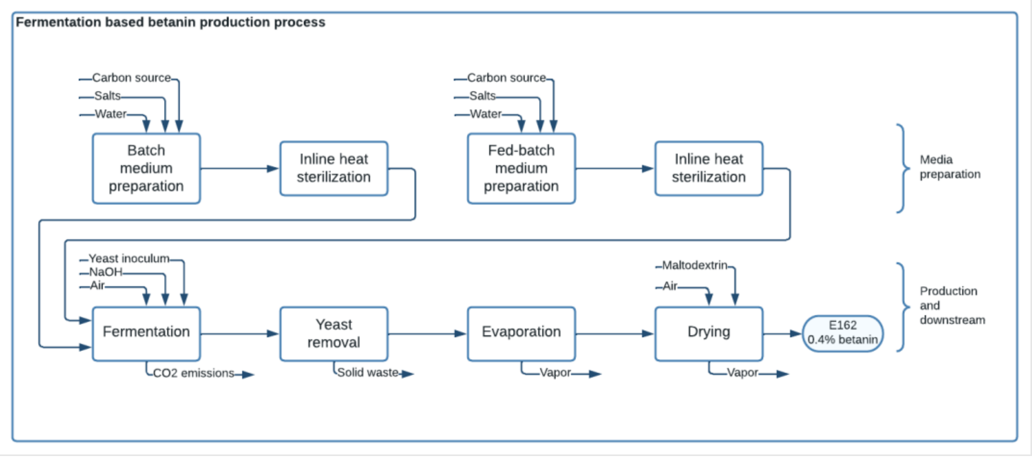

Supplementary Figure S4. Block Flow Diagram of the fermentation based betanin production process. Minor streams, utilities and CIP (Cleaning-In-Place), SIP (Steam-in-Place) waste flows are not reported.

Batch and fed-batch media are prepared and sterilized separately to ensure no cross-contamination. The media are sent to a fermenter in which the aerobic fermentation of a GMO strain of *Yarrowia lipolytica* transforms a carbon source into betanin. Sterile air is supplied through a gas compressor and an air microfilter. After a target titer of the product is achieved, the biomass is separated from the broth through a basket centrifuge. The filtrate is then concentrated in a thin film evaporator until a 10-fold concentration is reached and spray dried onto maltodextrin until a final concentration of 0.4% betanin in the powder product is achieved. Each equipment at the end of the main operation is cleaned-in-place (CIP). The fermenter comprises several steps of cleaning, as described in Figure S5. The cleaning steps for the betanin colorant were taken as modeled in McCarthy et al.[21].

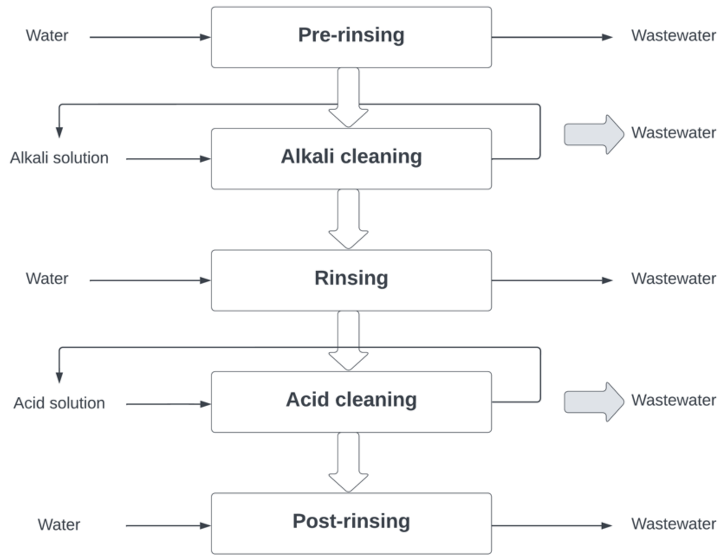

Supplementary Figure S5. Cleaning steps in the food industry

The model is then adapted to compare the economic performance using four different carbon sources: glucose, sucrose, molasses from sugar beet production, and glycerol. Experimental data on the titer were available for the glucose and glycerol feedstocks. In the sucrose and molasses case, the fermentation scientists provided an educated guess about the titer achieved. The assumptions and data of the fermentative process are described according to Table S9 to Table S11.

Supplementary Table S8. Equipment design parameters for the extraction-based process.

Unit operation	Selected equipment in SuperPro	SuperPro design parameter	Value	Unit
Charging of the beetroots	Belt conveyor	Intensive energy per throughput per length of the belt	0.012	W/kg m/h
Beetroot washing	Washer	Volume per mass of feed	7.728	L/kg
Shredding	Shredder	Intensive power per throughput	0.0009	kW/(kg/h)
Grinding	Grinder	Intensive power per throughput	0.0009	kW/(kg/h)
Pulp removal	Basket centrifuge	Intensive power per area	4.95	kW/m^2
Extraction	Stirred reactor	Intensive power per throughput	2.41	kW/(kg/s)
Filtration	Microfilter	Intensive power per area	0.173	kW/m^2
Fermentation	Stirred reactor	Intensive power per volume	3	kWh/m^3
Yeast removal	Basket centrifuge	Intensive power per area	4.95	kW/m^2
Concentration	Thin film evaporator	Evaporation rate for volatile components	90	% of water evaporated
Drying	Spray dryer	Evaporation rate for volatile components	99	% of water evaporated

4.4. Process design parameters used in the extraction-based process

The beet root composition was assumed to be 0,07% betanin, 10 % biomass, and 89.93% water remaining. The process design parameters for the extraction-based process are described in Table S9.

Supplementary Table S9. Process design parameters for the extraction-based process

Unit operation	Process design parameter	Value	unit
Charging of the beetroots	Losses	1%	%
Beetroot washing	Washing water recycling rate	0%	%
Shredding	Losses	1%	%

Grinding	Losses	1%	%
Pulp removal	Biomass removal	100%	%
Extraction	Extraction solvent	Citric acid 0.09% in water	%
	mass ratio of Extraction solvent vs beetroot juice	1.5	
	Juice retention (agitation duration)	51	min
Filtration	Recovery percentage (filtrate/feed)	96%	
Fermentation	Duration	608	min
	Ratio of Inoculum volume to inlet juice stream	1.38%	%
	Temperature	30	°C
	Host	*Candida utilis*	
	Oxygen uptake	0.52	mol CO_2/mol *C. utilis* biomass
	CO2 emissions	0.55	mol CO_2/mol *C. utilis* biomass
	pH adjustment medium (5M NaOH) volume ratio	0.084%	% vol/vol
Yeast removal	Solids removal	100%	%
Concentration	Water removal	90%	%
Drying	Water removal	99%	%
	Air temperature	70	°C
	Ratio of Maltodextrin added vs. final product	98%	%

Supplementary Table S10. Equipment design parameters for the fermentation-based process, valid for all the different feedstock scenarios

Unit operation	Selected equipment in SuperPro	SuperPro design parameter	Value	Unit
Media preparation	Blending tanks (2)	Intensive energy per volume	1.7	kW/m^3
Media sterilization	Heat sterilizer	Temperature	140	°C
Fermentation	Batch stoichiometric fermenter	Intensive energy per volume	1.7	kW/m^3
		CIP duration	30	min
		SIP duration	60	min
		Total turnover time	52.8	h
Biomass removal	Basket centrifuge	Intensive energy per area	4.95	kW/m^2
		Particulate component retention %	100	% yeast removed
		Loss on Drying (LOD)	30	%
		Maximum cake thickness	15	cm
		Maximum diameter	2.5	m
Concentration	Thin film evaporator	Vapor fraction	90	% of water evaporated
		Product concentration	10	Fold increase in concentration
		Temperature (isothermal operation)	50	°C
		Heat transfer efficiency	90	%
Drying	Spray dryer	Maltodextrin carrier addition	98.6	% to total E162 composition
		Specific evaporation rate	100	(kg/h)/m3

	Vapor fraction	90	% of water evaporated
	Temperature	70	°C

Table S11. Process design parameters for the fermentation-based process, valid for all the different feedstock scenarios

Unit operation	Process design parameter	Value	unit
Media preparation	Agitation duration	1	h
Media sterilization	Sterilization duration	1	h
Fermentation	Temperature	30	°C
	Pressure	1	atm
	Oxygen concentration	1	vvm
Biomass removal	Duration	4	h
	Losses	1	%
Concentration	Duration	18	h
	Losses	1	%
Drying	Duration	24	h
	Losses	1	%

The process reactions were calculated using the standard baker's yeast formation reaction, adding betanin as a product and adapting the biomass elemental composition known for the yeast *Yarrowia*. The ammonium sulfate dissociation reaction provides nitrogen as NH_4+ ions for biomass and product formation. The reactions enable automation of the consumption of carbon source and other raw materials, formulated in a stock recipe, and enabled with the "auto-adjust" function in SuperPro. Additionally, they enable the estimation of CO_2 emissions to the atmosphere, which is relevant economically and environmentally. The brute formula for betanin is: $C_{24}H_{26}N_2O_{13}$. The brute formula for *Y. lipolitica* is: $C_{10}H_{18}NO_5$ (the formula was rounded from $C_{10}H_{18.2}O_{5.1}N$ to allow the convergence of the reaction stoichiometry). The reactions were balanced using Wolphram Alpha's online calculator[22]. Table S12 contains the balanced reactions used in the fermenter.

Supplementary Table S12. Process reactions used as input in the stoichiometric reactor

Feedstock	Reaction stoichiometry
Any – salt dissociation	$(NH_4)_2SO_4$ = 2 NH_4+ + 2 $SO_{4(2-)}$
Glucose	8 $C_6H_{12}O_6$ + 2 NH_4+ 3 O_2 = $C_{24}H_{26}N_2O_{13}$ + 4 CO_2 + 2 $C_{10}H_{18}NO_5$ + 29 H_2O
Sucrose	3 $C_{12}H_{22}O_{11}$ + 3 NH_4+ 3 O_2 = $C_{24}H_{26}N_2O_{13}$ + 2 CO_2 + 1 $C_{10}H_{18}NO_5$ + 17 H_2O
Glycerol	16 $C_3H_8O_3$ + 2 NH_4+ + 11 O_2 = $C_{24}H_{26}N_2O_{13}$ + 4 CO_2 + 2 $C_{10}H_{18}NO_5$ + 39 H_2O

For molasses, the sugar beet molasses is simulated using a user-defined stock mixture composed of 43% sucrose and the remaining water. Therefore, the equation is the same as sucrose. Table S13 and Table S14 summarize the components concentration in the batch and fed-batch media. The trace elements were neglected because of the presence in quantities less than the 0,15% (all trace elements summed up) by weight.

Supplementary Table S13. Batch medium composition, valid for all the four feedstock scenarios

Component	Value	Unit
Ammonium Sulfate	10	g/L
Potassium Di-hydrogen Phosphate	6	g/L
Magnesium Sulfate	1	g/L
Yeast extract	10	g/L

Supplementary Table S14. Batch and Fed-Batch medium composition of the main carbon source for each scenario

Component	Value	Unit
Glucose, batch	40	g/L
Glucose, fed-batch	600	g/L
Sucrose, batch	40	g/L

Sucrose, fed-batch	600	g/L
Molasses, batch	40	g/L[1]
Molasses, fed-batch	600	g/L[2]
Glycerol, batch	56	g/L
Glycerol, fed-batch	600	g/L

[1]Based on the net content of sucrose in the molasses, simulated as 43% sucrose and 57% water.
[2]Same as note [1].

4.5. Prices (for all processes)

The prices presented here were retrieved from the online subscription-based database Import Genius[23]. The scope of using an import-export bulk database is to find commodity prices for the raw materials used, to reflect the case of a real trade. The data is filtered in the Import Genius(https://www.importgenius.com/) search engine according to the HS code of each compound, to ensure consistency. For each compound, the first and third percentile are extracted, along with the median. The median was used in the base case scenarios as the baseline price, while the distribution of the prices was used to illustrate the uncertainty in the production cost due to feedstock price fluctuations.

Supplementary Table S15. Prices of the raw materials used in the TEA

Component	25th percentile [USD/kg]	Median [USD/kg]	75th percentile [USD/kg]	Standard deviation	Standard Error
Ammonium sulphate	0.15	0.170	0.20	0.167	0.002
Potassium phosphate	0.62	0.681	0.92	25.038	0.464
Magnesium sulphate	0.47	0.480	4.27	21.792	1.090
D-glucose monohydrate	0.17	0.177	0.22	0.046	0.002
Sucrose	0.30	0.35	0.37	38.124	0.381
Glycerol	1.15	1.525	1.88	0.642	0.007
Sugar beet molasses	0.031	0.03	0.031	58.962	0.541
NaOH pure, flakes	0.49	0.760	8.19	12.450	0.259
Water	0.0020	0.0033	0.0040	-	-
Maltodextrin	2.07	2.3	2.36	4.846	0.031
Yeast extract	3.7	5.63	8.6	18.205	0.063

Supplementary Table S16. Cost of other utilities and waste treatment for Germany

Aspect or item	Price	Unit
CO_2 emitted, cost (Germany)	0.062927	USD/m^3(STP)
GMO waste disposal (Belgium)	13	USD/m^3

Disposal cost of failed product	0.01	USD/kg
Electricity	0.179	USD/kWh
CIP medium - acid	0.004005	USD/m^3(STP)
CIP medium - basic	0.004005	USD/m^3(STP)

4.6. Financial parameters and their baseline values for Germany

Table S17. Financial parameters and their baseline values for Germany

Aspect	Value	Unit
Inflation rate (2021)	3.1[1]	%
Business loan interest rate	3.45[2]	
NPV interest (medium)	9	%
Debt percentage	90	%
Loan period	10	years
Working capital	10	%
Upfront R&D	10	%
Upfront royalties	0	%
Depreciation period	10	years
Direct Financed Capital (DFC) imbursed (1styear, 2nd year, 3rd year)	30,40,30	%
Salvage value	5	%
Corporate taxation rate on revenue	29.8	%
Advertising	0.5	% of final selling price
Royalties	0.5	% of final selling price

4.7. Process plant parameters and their baseline values for Germany

Table S18. Process plant parameters and their baseline values

Item	Value	Unit
Construction period	30	months
Startup period	6	months
Plant lifetime	20	years
Operating capacity 1st year	30	%
Operating capacity 2nd year	50	%
Operating capacity 1st year	80	%
Product failure rate	15	%

4.8. Sensitivity analysis methodology

A sensitivity analysis was applied by varying process parameters like titer, plant capacity, product selling price, and carbon sources (feedstocks). Firstly, a feedstock sensitivity was performed assessing the process using glucose, glycerol, sucrose, and molasses were compared based on (i) the same payback period, 3 years; (ii) experimental titer, where available, or assumed experimental

titer. The capacity of the plant varied until the target was achieved. Secondly, titer was varied between 50% to +400% with respect to the baseline scenarios for each feedstock and price scenario. The third sensitivity parameter was the plant capacity, which varied between the boundaries set for the market volume for each product price, as reported in Table S6. The analysis was performed for each feedstock. Finally, different product selling prices were assessed (values ranging as given in Table S4), where the minimum price is recalculated at 21.5 USD/kg.

5. Techno-economic assessment results

5.1. Base case scenarios

Supplementary Table S19. Summary of results for the economic analysis – baseline scenarios

Parameter	Glucose	Sucrose	Molasses	Glycerol
Annual Operating time, net [h]	7905	7902	7885	7895
Number of batches	149	149	144	216
Plant throughput [t/y]	550	491.7	504	688
Plant throughput [t/batch]	3.7	3.3	3.5	3.2
Product titer at harvest g/L	1.10	1.00	1.00	0.62
Yield [kg product/kg sucrose]	1.82%	1.36%	1.81%	0.65%
Fermentation cycle time (Including cleaning operations) [h]	52	52	52	34
Fermentation duration[h]	42	42	42	24
Fermentation failure rate	15%	15%	15%	15%
**Major equipment**				
Fermentation reactors [number of units]	1	1	1	1
Fermentation reactors size, each, total volume [m3]	17.5	17.6	18	25.5
Centrifuge [number of units]	2	2	2	3
Centrifuge [m2]	9.6	9.3	9.3	9.3
Evaporator [number of units]	1	1	1	1
Evaporator [m2]	1.73	1.69	1.77	2.45
Spray dryer [number of units]	1	1	1	1
Spray dryer [m3]	0.56	0.53	0.56	0.77
**Resume of principal financial indexes outcome**				
NPV (at 7%)	50,554,467	39,016,051	38,106,254	60,476,113
IRR (%)	52.74	40.53	38.82	51.27
ROI (%)	33.4	33.16	32.24	32.33
PBP (years)	2.99	3.02	3.1	3.09
Total capital investment (USD)	29,036,000	27,753,000	28,553,000	36,286,000

5.2. Sensitivity scenarios results

The sensitivity analysis was performed to assess key parameters of the economic model that might affect processes performance. This assessment also allows predicting performances under hypothetical scenarios to provide insights into the system's elasticity to hold variations and

uncertainty. Based on the market data, the payback period (PBP) was assessed at different prices by simulating variations in the fermentation titer (see Figure S6). The results indicate that glycerol fermentation gives the lowest payback period at base case titer, while molasses shows the highest (however, by a small margin). Increasing the price from 21.50 USD/kg to 34.75 USD/kg results in a drastic improvement of the PBP in all cases, while the change in the PBP is not as dramatic from the case of 35.75 USD/kg to 61 USD/kg.

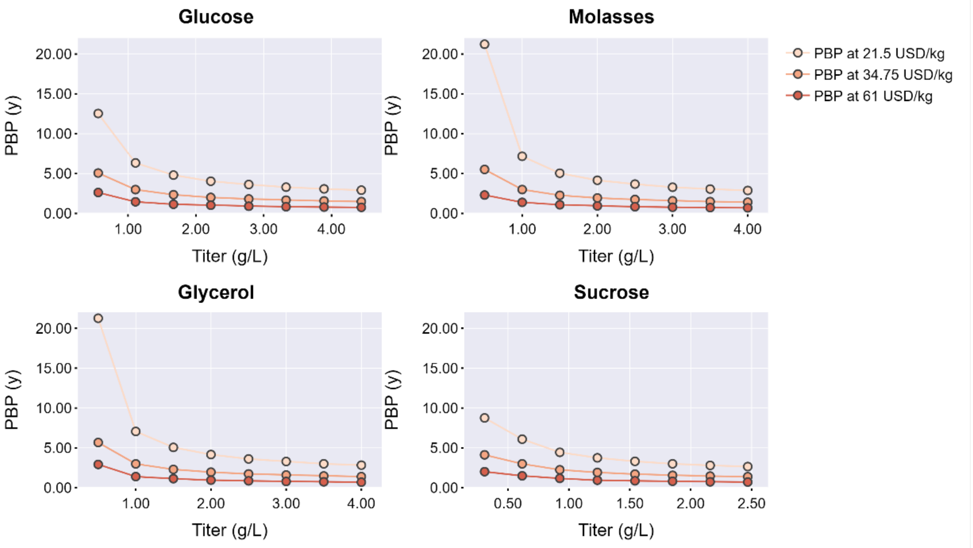

Supplementary Figure S6. Payback period (PBP) at three prices (high, mid, and low) by changes in fermentation titer for different feedstocks.

As we wanted to analyze the effects of titer variations further, we performed a sensitivity of the production cost. This sensitivity provides insights into how cheap the process could become by improvements in the fermentation process (see Figure S7).

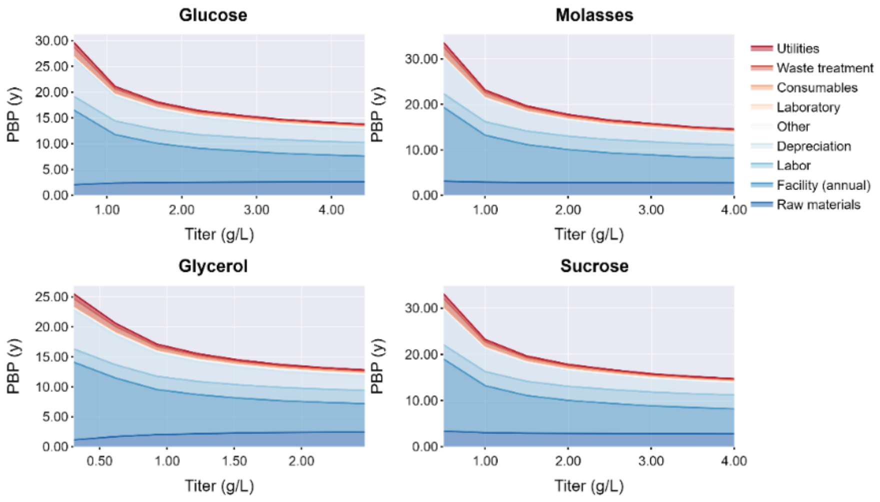

Supplementary Figure S7. Sensitivity of payback period to feedstock and titer variations

A final sensitivity was performed to analyze the effects of production capacity variations in the production cost of the plant (see Figure S8). Production cost was evaluated as a key parameter in the

decision-making of a production plant since this parameter gives a direct metric to benchmark process performance against other alternatives, market/ industrial standards, or decisions based on stakeholders' expectations[24]. Results indicate that the four feedstock cases behave similarly with the same trend for all costs. This trend is expected since the main difference between these processes is the feedstock, whereas the process setting remains the same. The major cost-driver is facility cost, followed by labor and raw material cost. The obtained cost contributions are an expected outcome considering the requirement for specialized equipment to produce betanin in fermentation cycles. The results indicate that the different fermentation processes have a similar production cost, which is highly sensitivity to the facility size. This means that to reach lower production costs, the production should be operated at higher capacities (greater than 500 t/y).

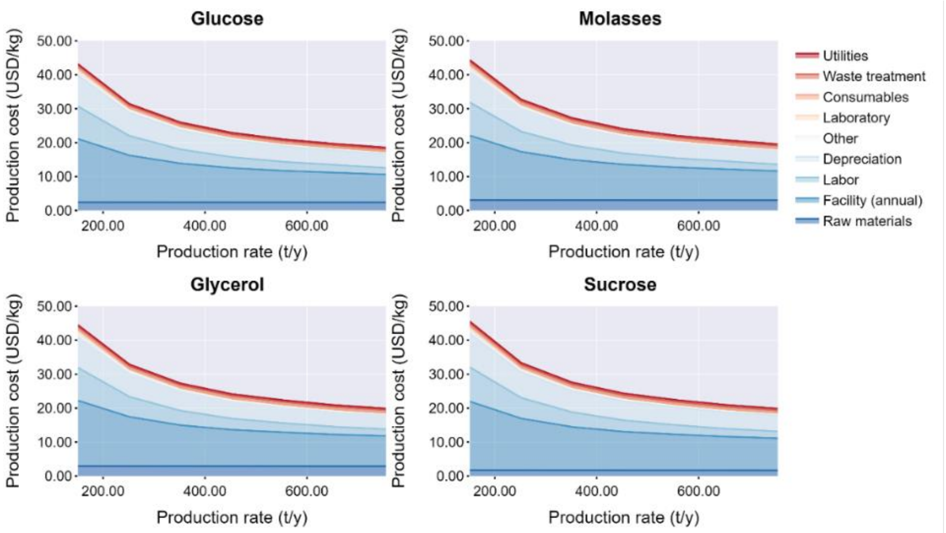

Supplementary Figure S9. Sensitivity of production cost by changes in production rate for different feedstocks.

5.3. Uncertainty analysis results

The uncertainty analysis becomes a relevant tool to estimate or predict expected variations in production cost because of market dynamics. This study performed a sensitivity analysis to assess such variations in the product cost of glucose, sucrose, and molasses cases (see Figure S9). The procedure was as follows: (i) the raw data was taken from Import genius and curated to get bulk quantity prices, (ii) a statistical analysis was performed to estimate the minimum, mode, and maximum values. Evaluation methods were applied to create a distribution that represents realistic price fluctuations. The reason to do this was that the data used was not completely independent. Treating it as an independent variable would have led to unreasonable estimates. Hence, the copula probability theory was applied. In python, there is a library that transforms real dependent data into synthetic samples, while preserving the relationships/dependencies. This is how the synthetic data was generated. The analysis only works with three parameters per time, and three sets of data were

generated, for glucose, sucrose, and molasses, because these were the top three expensive compounds in the raw materials. There were not enough data points for glycerol therefore it was skipped. The combinations of the data were inputted to each single simulation to generate production cost results as shown in Figure S9.

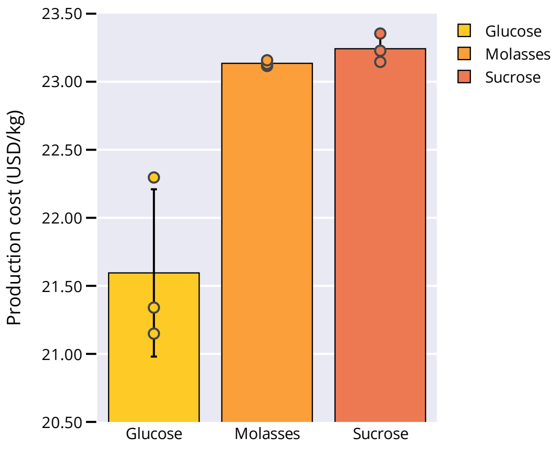

Supplementary Figure S9. Uncertainty analysis of operating product cost, bars and error bars represent the mean of production cost (USD/kg) and standard deviation of each feedstock scenario, and the overlaying dots (n=3) represent the cost datapoints.

6. Life Cycle Assessment supporting data

A life cycle assessment was performed for the new fermentation-based betanin production. The analysis also included the assessment of the extraction-based betanin production. The data was generated through process simulation in both cases (using the setup described in the previous sections). As described in section 1 of this supplementary file, we applied a methodology (see Figure S1) to assess the sustainability performance of the betanin-based colorant production.

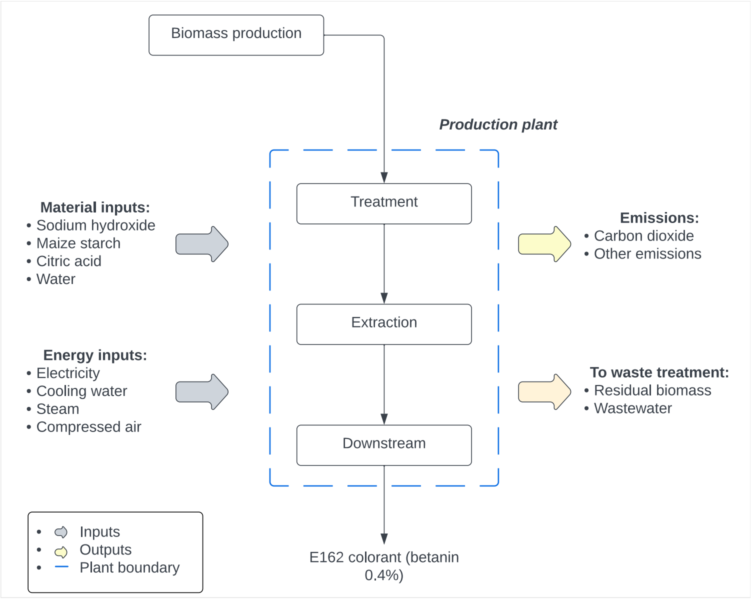

Supplementary Figure S10. System boundaries of betanin-colorant production

The methodology involved five major stages, namely, (i) experiments, (ii) process model, (iii) inventory, and (iv) Techno-economic assessment, in parallel with (v) Life Cycle Assessment. This section will describe the supporting data on the Life Cycle Assessment phase. The functional unit was defined as 1 kg of E162 colorant (0.4% betanin content), covering a cradle-to-gate system boundary, as shown in Figure S10.

6.1. Life Cycle inventory

The Life Cycle Inventory (LCI) was modeled using process simulation results that contained the required mass and energy balance of extraction- and fermentation-based betanin production. The input and output flows provided in Table S20 describe the foreground system data used to model the base cases. Sources for the background system are given in the following sections. Some assumptions were applied in the modeling of LCI considering data limitations:

- Monopotassium sulfate is modeled as sodium phosphate since this component is missing in Ecoinvent 3.8 database.
- Cooling water is modeled assuming the operation of cooling towers allowing for recirculating water streams curtailing the requirement for process-water. A loss of 1% is assumed in the cooling towers.
- Maltodextrin is modeled as maize starch since this component is missing in Ecoinvent 3.8 database.

Supplementary Table S20. Process inventory for extraction- and fermentation-based processes for 1 kg of product (base cases)

Component/flow	Extraction	Glucose	Glycerol	Molasses	Sucrose	Unit
			Inputs (material)			
Beetroot	11.23	0.00	0.00	0.00	0.00	kg
Molasses	0.00	0.00	0.00	0.61	0.00	kg
Glucose	0.00	0.26	0.00	0.00	0.00	kg
Glycerol	0.00	0.00	0.72	0.00	0.00	kg
Sucrose	0.00	0.00	0.00	Note[1]	0.35	kg
Citric acid	0.01	0.00	0.00	0.00	0.00	kg
Water	26.03	15.76	27.07	17.43	17.89	kg
Ammonium sulfate	0.00	0.04	0.06	0.04	0.04	kg
Monopotassium phosphate	0.00	0.02	0.04	0.02	0.03	kg
Magnesium sulfate	0.00	3.71×10^{-3}	6.2×10^{-3}	3.57×10^{-3}	4.20×10^{-3}	kg
Sodium hydroxide	4.21	0.02	0.05	0.05	0.03	kg
Maize starch (Carrier)	0.45	0.89	0.40	1.13	1.13	kg
			Input (utilities)			

Cooling water	277	648.09	1009.96	723.59	724.94	kg
Steam	7.39	5.66	10.03	6.43	6.32	kg
Electricity	1.022	1.80	1.93	1.96	2.02	kWh
Compressed air	24.84	14.90	16.06	16.23	16.75	m^3
		Output (material)				
Biomass	11.36	0.16	0.28	0.54	0.66	kg
Carbon dioxide	0.21	7.60×10^{-4}	2.8×10^{-4}	7.85×10^{-4}	7.86×10^{-4}	kg
Wastewater	36.99	8.30	17.70	13.13	9.41	kg
Colorant E162	1.00	1.00	1.00	1.00	1.00	kg

Uncertainty LCI input data

To implement uncertainty analysis, statistical input data was provided to model the product system flows and perform Monte Carlo simulations. The background system used the distributions and uncertainty data given by Ecoinvent 3.8 database, while the foreground systems (betanin production) were assigned the lognormal distribution since, this approach models LCI data more appropriately and does not allow for negative (which would be unrealistic) data points. The geometric mean for each flow is assigned based on the Pedigree matrix approach (see Table S21), which assesses data based on quality and representativeness[25].

Supplementary Table S21. Pedigree matrix criteria for assigning geometric means.

Indicator score	Reliability	Completeness	Temporal correlation	Geographical correlation	Further technological correlation
1	1.00	1.00	1.00	1.00	1.00
2	1.54	1.03	1.03	1.04	1.18
3	1.61	1.04	1.10	1.08	1.65
4	1.69	1.08	1.19	1.11	2.08
5	1.69	1.08	1.29	1.11	2.80

Product system assemblies

Assemblies were built using the foreground data from Table S20 to model the product system. The assemblies comprise feedstock production, processing material flows (without utilities), and processing utilities as product systems assessed in this work, considering different routes (fermentation and extraction) and feedstocks. Tables S22 to S26 provide the details of product system assemblies. Similarly, sources of background data and corresponding geometric mean values (to flows) are described in Table S27. As we conducted a sensitivity analysis to interpret results, details of the normalized inventories to the functional unit are given in Tables S28 to S31.

Supplementary Table S22. Process assembly inventory for betanin production via extraction

Products	Amount	unit	Distribution	Geometric mean
E162 {DE}, betanin 0.4%, extraction from beet	1	p		N/A
**Materials**				
Sugar beet {DE}| sugar beet production to betanin | Cut-off, U	11.23	kg	Lognormal	1.08
Colorant E162 {DE}, extraction - Betanin Processing (without utilities)	1	kg	Lognormal	1.08
Colorant E162 {DE}, extraction - Utilities	1	kg	Lognormal	1.08

Supplementary Table S23. Process assembly inventory for betanin production via glucose fermentation

Products	Amount	unit	Distribution	Geometric mean
E162 {DE}, betanin 0.4%, fermentation from glucose	1	p		N/A
**Materials**				
Glucose {DE}| glucose production | Cut-off, U	0.1875	kg	Lognormal	1.08
Colorant E162 {DE}, fermentation from glucose - Betanin Processing (without utilities)	1	kg	Lognormal	1.08
Colorant E162 {DE}, fermentation from glucose - Utilities	1	kg	Lognormal	1.08

Supplementary Table S24. Process assembly inventory for betanin production via glycerol fermentation

Products	Amount	unit	Distribution	Geometric mean
E162 {DE}, betanin 0.4%, fermentation from glycerol	1	p		N/A
**Materials**				
Glycerol {DE}| market from glycerin | Cut-off, U	0.64	kg	Lognormal	1.08
Colorant E162 {DE}, fermentation from glycerin - Betanin Processing (without utilities)	1	kg	Lognormal	1.08
Colorant E162 {DE}, fermentation from glycerin - Utilities	1	kg	Lognormal	1.08

Supplementary Table S25. Process assembly inventory for betanin production via molasses fermentation

Products	Amount	unit	Distribution	Geometric mean
E162 {DE}, betanin 0.4%, fermentation from molasses	1	p		N/A
**Materials**				
Molasses {GLO}| market from sugar beet| Cut-off, U	0.32	kg	Lognormal	1.08
Colorant E162 {DE}, fermentation from molasses - Betanin Processing (without utilities)	1	kg	Lognormal	1.08
Colorant E162 {DE}, fermentation from molasses - Utilities	1	kg	Lognormal	1.08

Supplementary Table S26. Process assembly inventory for betanin production via sucrose fermentation

Products	Amount	unit	Distribution	Geometric mean
E162 {DE}, betanin 0.4%, fermentation from sucrose	1	p		N/A
**Materials**				
Sugar {GLO}| from sugar beet| Cut-off, U	0.186	kg	Lognormal	1.08
Colorant E162 {DE}, fermentation from sugar- Betanin Processing (without utilities)	1	kg	Lognormal	1.08
Colorant E162 {DE}, fermentation from sugar - Utilities	1	kg	Lognormal	1.08

Supplementary Table S27. Sources of background system data

Model in Ecoinvent	Substance	Geometric mean
Ammonium sulfate {RER}| market for ammonium sulfate | Cut-off, U	Ammonium sulfate	1.08
Sodium chloride, powder {RER}| production | Cut-off, U	Sodium chloride	1.08
Magnesium sulfate {RER}| production | Cut-off, U	Magnesium sulfate	1.08
Calcium chloride {RER}| market for calcium chloride | Cut-off, U	Calcium chloride	1.08
Sodium hydroxide, without water, in 50% solution state {GLO}| market for | Cut-off, U	Sodium hydroxide	1.08

Maize starch {DE}| production | Cut-off, U	Carrier agent (assumed as maize starch since maltodextrin is missing in Ecoinvent 3.8)	1.08
Sodium phosphate {RER}| market for sodium phosphate | Cut-off, U	Sodium phosphate is used instead of potassium phosphate	1.57
**Model in ecoinvent**	**Substance/Utility**	**Geometric mean**
Water, cooling, surface	Cooling water	Undefined
Electricity, medium voltage	Electricity for operation	1.08
Steam	Steam for heating	1.08
Electricity, medium voltage	Electricity consumption of cooling towers/suppliers for cooling	1.08
Compressed air, 600 kPa gauge	Compressed air	1.08
Tap water, market for | cut-off, U	Water intake	1.08
**Emissions model in Ecoinvent**	**Emissions**	**Geometric mean**
Carbon dioxide, biogenic	Carbon dioxide emissions	1.5
**Waste to treatment model in Ecoinvent**	**Waste flows**	**Geometric mean**
Biowaste {GLO}| treatment of biowaste, municipal incineration | Cut-off, U	Residual biomass	1.5
Wastewater, average {Europe without Switzerland} | market for wastewater, average | Cut-off, U	Wastewater	1.5

Supplementary Table S28. normalized inventory of sensitivity scenarios for glucose process

Substance	Input or output	Titer (g/L)							
		**0.56 g/L**	**1.11 g/L**	**1.67 g/L**	**2.22 g/L**	**2.78 g/L**	**3.33 g/L**	**3.89 g/L**	**4.4 g/L**
**Ammonium Sulfate**	In	0.07	0.04	0.02	0.02	0.01	0.01	0.01	0.01
**Monopotassium phosphate**	In	0.04	0.02	0.01	0.01	0.01	0.01	0.01	0.01
**Magnesium Sulfate**	In	0.01	0.00	0.00	0.00	0.00	0.00	0.00	0.00
**Maltodextrin**	In	0.60	0.89	0.99	1.03	1.06	1.08	1.10	1.11

**Nitrogen**	In	22.85	11.43	7.62	5.71	4.57	3.81	3.27	2.86
**Oxygen**	In	6.94	3.47	2.31	1.73	1.39	1.16	0.99	0.87
**Sodium Hydroxide**	In	0.04	0.02	0.01	0.01	0.01	0.01	0.01	0.01
**Glucose**	In	0.52	0.26	0.17	0.13	0.10	0.09	0.07	0.06
**Water**	In	26.75	15.76	9.80	8.43	7.58	7.04	6.63	6.31
**Sulfuric acid**	In	4.38E-04	2.18E-04	1.45E-04	1.09E-04	8.73E-05	7.27E-05	6.18E-05	5.45E-05
**Yeast extract**	In	0.07	0.04	0.02	0.02	0.01	0.01	0.01	0.01
**Steam**	In	11.35	5.66	3.77	2.82	2.25	1.87	1.60	1.40
**Cooling Water**	In	1295.82	648.09	432.18	324.23	259.46	216.28	185.43	162.30
**Chilled Water**	In	71.12	35.56	23.71	17.78	14.23	11.86	10.16	8.89
**Electricity**	In	3.61	1.80	1.20	0.90	0.72	0.60	0.52	0.45
**Carbon Dioxide**	Out	7.61E-04	7.61E-04	7.61E-04	7.61E-04	7.61E-04	7.61E-04	7.61E-04	7.61E-04
**Biomass**	Out	0.31	0.16	0.10	0.08	0.06	0.05	0.04	0.04
**Water**	Out	12.36	8.30	6.88	6.16	5.71	5.42	5.20	5.02
**Electricity (cooling towers)**	Out	0.01	0.01	0.01	0.01	0.01	0.01	0.01	0.01
**Betanin**	Out	1.00	1.00	1.00	1.00	1.00	1.00	1.00	1.00

Supplementary Table S29. normalized inventory to 1 kg of product for glycerol sensitivity scenarios

Substance	Unit	Input or output	Titer (g/L)							
			**0.31**	**0.62**	**0.93**	**1.23**	**1.54**	**1.85**	**2.16**	**2.47**
**Ammonium sulfate**	kg	In	0.09	0.06	0.04	0.03	0.02	0.02	0.02	0.02
**Monopotassium phosphate**	kg	In	0.06	0.04	0.02	0.02	0.01	0.01	0.01	0.01
**Magnesium sulfate**	kg	In	9.36E-03	6.21E-03	4.12E-03	3.09E-03	2.47E-03	2.06E-03	1.77E-03	1.55E-03
**Maltodextrin**	kg	In	0.00	0.40	0.66	0.79	0.87	0.92	0.96	0.98
**Nitrogen**	kg	In	18.64	12.32	8.21	6.16	4.93	4.11	3.52	3.08
**Oxygen**	kg	In	5.66	3.74	2.49	1.87	1.50	1.25	1.07	0.94
**Sodium Hydroxide**	kg	In	0.08	0.05	0.03	0.03	0.02	0.02	0.01	0.01
**Glycerol**	kg	In	1.10	0.72	0.48	0.36	0.29	0.24	0.21	0.18
**Water**	kg	In	26.75	15.76	9.80	8.43	7.58	7.04	6.63	6.31
**Sulfuric acid**	kg	In	5.80E-04	3.85E-04	2.56E-04	1.92E-04	1.54E-04	1.28E-04	1.10E-04	0.00

**Yeast extract**	kg	In	37.40	27.07	18.79	16.47	11.12	10.17	9.47	8.93
**Steam**	kg	In	0.09	0.06	0.04	0.03	0.02	0.02	0.02	0.02
**Cooling Water**	kg	In	15.13	10.03	6.65	4.98	3.98	3.31	2.83	2.48
**Chilled Water**	kg	In	1528.26	1009.96	673.50	505.28	404.34	337.05	288.98	252.93
**Electricity**	kWh	In	57.96	38.29	25.53	19.15	15.32	12.77	10.94	9.58
**Carbon Dioxide**	kg	Out	2.90	1.93	1.28	0.96	0.77	0.64	0.55	0.48
**Biomass**	kg	Out	0.42	0.28	0.18	0.14	0.11	0.09	0.08	0.07
**Water**	kg	Out	0.00	0.00	0.00	0.00	0.00	0.00	0.00	0.00
**Electricity (cooling towers)**	kWh	Out	24.01	17.70	13.53	11.42	10.13	9.28	8.66	8.17
**Betanin**	kg	Out	0.01	0.01	0.01	0.01	0.01	0.01	0.01	0.01

Supplementary Table S30. normalized inventory of sensitivity scenarios for molasses process

Substance	Input or output	Titer (g/L)							
		**0.50**	**1.00**	**1.50**	**2.00**	**2.50**	**3.00**	**3.50**	**4.00**
**Ammonium Sulfate**	In	0.07	0.04	0.02	0.02	0.01	0.01	0.01	0.01
**Monopotassium phosphate**	In	0.04	0.02	0.01	0.01	0.01	0.01	0.01	0.01
**Magnesium Sulfate**	In	7.10E-03	3.57E-03	2.37E-03	1.77E-03	1.42E-03	1.18E-03	1.01E-03	8.85E-04
**Maltodextrin**	In	1.09	1.13	1.14	1.15	1.15	1.16	1.16	1.16
**Nitrogen**	In	24.91	12.45	8.30	6.22	4.97	4.14	3.55	3.11
**Oxygen**	In	7.56	3.78	2.52	1.89	1.51	1.26	1.08	0.94
**Sodium Hydroxide**	In	0.09	0.05	0.03	0.02	0.02	0.02	0.01	0.01
**Sucrose**	In	0.52	0.26	0.17	0.13	0.10	0.09	0.07	0.06
**Sulfuric acid**	In	4.94E-04	2.48E-04	1.65E-04	1.23E-04	9.92E-05	8.33E-05	7.14E-05	6.15E-05
**Water**	In	32.52	17.43	10.95	9.38	8.44	7.81	7.37	7.01
**Yeast extract**	In	0.07	0.04	0.02	0.02	0.01	0.01	0.01	0.01
**Steam**	In	12.79	6.43	4.25	3.18	2.54	2.11	1.80	1.58
**Cooling Water**	In	1447.06	723.59	482.44	361.86	289.51	241.28	206.83	180.99
**Chilled Water**	In	77.83	38.90	25.92	19.43	15.54	12.95	11.09	9.70
**Electricity**	In	3.92	1.96	1.31	1.75	0.78	0.65	0.56	0.49

**Carbon Dioxide**	Out	7.81E-04	7.86E-04	7.80E-04	7.79E-04	7.79E-04	7.79E-04	7.78E-04	7.78E-04
**Biomass**	Out	1.10	0.54	0.36	0.26	0.21	0.17	0.15	0.13
**Water**	Out	21.54	13.13	10.24	8.77	7.88	7.29	6.87	6.54
**Electricity (cooling towers)**	Out	0.01	0.01	0.01	0.01	0.01	0.01	0.01	0.01
**Betanin**	Out	1.00	1.00	1.00	1.00	1.00	1.00	1.00	1.00

Supplementary Table S31. normalized inventory of sensitivity scenarios for sucrose process

Substance	Input or output	Titer (g/L)							
		**0.50**	**1.00**	**1.50**	**2.00**	**2.50**	**3.00**	**3.50**	**4.00**
**Ammonium sulfate**	In	0.08	0.04	0.03	0.02	0.02	0.01	0.01	0.0
**Monopotassium phosphate**	In	0.05	0.03	0.02	0.01	0.01	0.01	0.01	0.0
**Magnesium sulfate**	In	8.42E-03	4.21E-03	2.80E-03	2.10E-03	1.68E-03	1.40E-03	1.20E-03	1.05E-03
**Maltodextrin**	In	1.09	1.13	1.14	1.15	1.15	1.16	1.16	1.2
**Nitrogen**	In	25.70	12.85	8.56	6.42	5.13	4.28	3.66	3.2
**Oxygen**	In	7.80	3.90	2.60	1.95	1.56	1.30	1.11	1.0
**Sodium Hydroxide**	In	0.05	0.03	0.02	0.01	0.01	0.01	0.01	0.0
**Sucrose**	In	0.69	0.35	0.23	0.17	0.14	0.11	0.10	0.1
**Sulfuric acid**	In	4.88E-04	2.44E-04	1.63E-04	1.22E-04	9.76E-05	8.14E-05	6.91E-05	6.10E-05
**Water**	In	30.42	17.89	11.15	9.58	8.60	7.98	7.50	7.1
**Yeast extract**	In	0.08	0.04	0.03	0.02	0.02	0.01	0.01	0.0
**Steam**	In	12.67	6.32	4.20	3.15	2.51	2.09	1.79	1.6
**Cooling Water**	In	1449.76	724.94	483.33	362.52	290.04	241.72	207.21	181.3
**Chilled Water**	In	80.78	40.37	26.90	20.17	16.13	13.44	11.51	10.1
**Electricity**	In	4.04	2.02	2.41	1.01	0.81	0.67	0.58	0.5
**Carbon Dioxide**	Out	7.87E-04	7.86E-04	7.86E-04	7.86E-04	7.85E-04	7.85E-04	7.84E-04	7.84E-04
**Biomass**	Out	1.33	0.66	0.43	0.32	0.25	0.21	0.18	0.2
**Water**	Out	14.05	9.41	7.79	6.97	6.45	6.12	5.87	5.7
**Electricity (cooling towers)**	Out	0.01	0.01	0.01	0.01	0.01	0.01	0.01	0.01
**Betanin**	Out	1.00	1.00	1.00	1.00	1.00	1.00	1.00	1.00

5.2. Life Cycle Impact Assessment results

Midpoint results

The midpoints provide insights into the environmental impacts somewhere in the cause-effect chain once the burden is emitted or released from the product system. The midpoints of the evaluated processes were estimated based on the ReCiPe 2016 methodology, which includes 18 categories. Table S32 reports the midpoint impact results of fermentation-based betanin production for each feedstock at base case titer.

Supplementary Table S32. Midpoint results of fermentation-based betanin production

Category	Unit	Glucose	Molasses	Glycerol	Sucrose
Fine particulate matter formation	kg PM2.5 eq	1,91E-03	2,10E-03	3,09E-03	2,15E-03
Fossil resource scarcity	kg oil eq	0,88	0,98	1,35	0,98
Freshwater ecotoxicity	kg 1,4-DCB	0,09	0,10	0,11	0,10
Freshwater eutrophication	kg P eq	1,89E-03	2,05E-03	2,18E-03	2,11E-03
Global warming	kg CO2 eq	2,97	3,29	4,43	3,33
Human carcinogenic toxicity	kg 1,4-DCB	0,15	0,17	0,20	0,17
Human non-carcinogenic toxicity	kg 1,4-DCB	2,44	2,65	3,01	2,74
Ionizing radiation	kBq Co-60 eq	0,30	0,33	0,35	0,34
Land use	m2a crop eq	0,47	0,46	0,78	0,54
Marine ecotoxicity	kg 1,4-DCB	0,12	0,13	0,15	0,14
Marine eutrophication	kg N eq	2,60E-04	2,97E-04	3,95E-04	2,93E-04
Mineral resource scarcity	kg Cu eq	4,04E-03	4,21E-03	6,04E-03	4,56E-03
Ozone formation, Human health	kg NOx eq	2,74E-03	3,69E-03	4,03E-03	3,92E-03
Ozone formation, Terrestrial ecosystems	kg NOx eq	2,98E-03	4,36E-03	4,15E-03	4,72E-03
Stratospheric ozone depletion	kg CFC11 eq	1,66E-06	2,01E-06	2,29E-06	2,18E-06

Terrestrial acidification	kg SO2 eq	5,55E-03	6,07E-03	9,10E-03	6,24E-03
Terrestrial ecotoxicity	kg 1,4-DCB	5,13	5,55	8,06	5,77
Water consumption	m3	0,72	0,80	1,10	0,81

Endpoint results

The endpoint, as opposed to midpoints, provides insights into the environmental impacts of the cause-effect chain. So, they are related to the impacts on the areas of protection (Human health, Ecosystem quality, and Resources). The endpoints were calculated based on the ReCiPe 2016 methodology. This methodology can provide results for both midpoint and endpoint impacts. Table S33 reports the endpoint impact results of fermentation-based betanin production for each feedstock at base case titer. Figure S11 shows endpoint results of different feedstock scenarios including uncertainty values. At the endpoint level, fermentative betanin processes have a superior environmental sustainability performance compared to the extraction-based process, even when accounting for uncertainties.

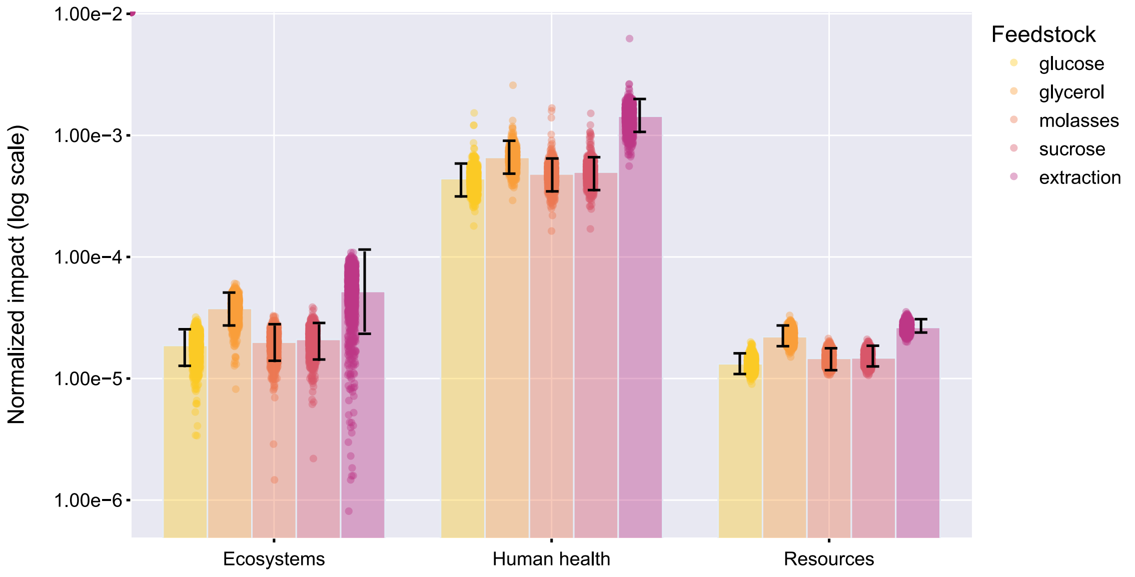

Supplementary Figure S11. Normalized midpoint impacts and uncertainty of fermentation-based scenarios varying feedstock (glucose, molasses, glycerol, and sucrose) compared with the extraction-based process. Data given in bars and error bars represent the mean and standard deviation of evaluated scenarios, dots represent the normalized impact points with n=1,000 simulations generated in the built-in Monte Carlo algorithm in SimaPro software.

Supplementary Table S33. Midpoint results of fermentation-based betanin production

Category	Unit	Glucose	Molasses	Glycerol	Sucrose
Human health	DALY	6,61E-06	7,29E-06	9,85E-06	7,42E-06
Ecosystem quality	species.yr	2,51E-08	2,74E-08	3,83E-08	2,84E-08
Resources	USD2013	0,24	0,27	0,40	0,27

Country sensitivity

Impacts on Human Health would increase moderately if the plant were built in China, mainly associated with a less environmentally friendly maltodextrin production (corn starch) and the extensive use of non-renewable energy sources in this country. Impacts on ecosystems quality are almost the same for Brazil and China in case glycerol is used as feedstock, most notably for impacts from burdens in marine eutrophication and land use associated with upstream corn starch. Conversely, impacts on the resources category became higher for the Germany location, followed by Brazil. In this case, the impact on resources is mostly associated with a lower resource scarcity impact since this category measures the cost for the extraction of fossil resources that for China's energy mix includes coal.

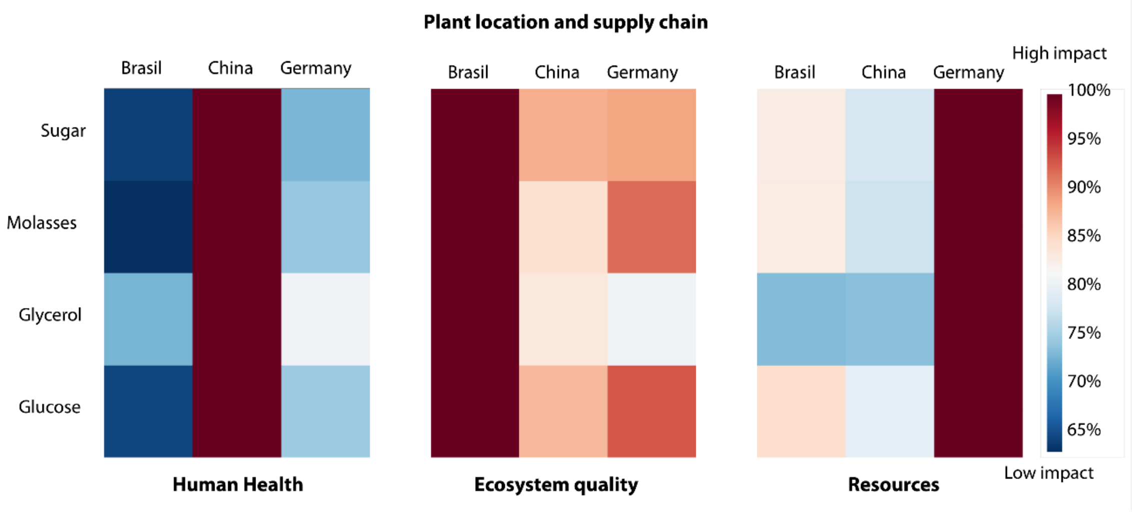

Supplementary Figure S11. Heatmaps of endpoint impacts by country sensitivity for different feedstocks.

References

1. Deloache, W. C. *et al.* An enzyme-coupled biosensor enables (S)-reticuline production in yeast from glucose. *Nat. Chem. Biol.* **11**, 465–471 (2015).

2. Babaei, M. *et al.* Combinatorial engineering of betalain biosynthesis pathway in yeast

Saccharomyces cerevisiae. *Biotechnol. Biofuels Bioprod.* 1–16 (2023) doi:10.1186/s13068-023-02374-4.

3. Sasaki, N. *et al.* Detection of DOPA 4,5-dioxygenase (DOD) activity using recombinant protein prepared from Escherichia coli cells Harboring cDNA encoding DOD from *Mirabilis jalapa*. *Plant Cell Physiol.* **50**, 1012–1016 (2009).

4. Vogt, T., Grimm, R. & Strack, D. Cloning and expression of a cDNA encoding betanidin 5-O-glucosyltransferase, a betanidin- and flavonoid-specific enzyme with high homology to inducible glucosyltransferases from the Solanaceae. *Plant J.* **19**, 509–519 (1999).

5. Sáez-Sáez, J. *et al.* Engineering the oleaginous yeast *Yarrowia lipolytica* for high-level resveratrol production. *Metab. Eng.* **62**, 51–61 (2020).

6. Gu, Y., Ma, J., Zhu, Y., Ding, X. & Xu, P. Engineering *Yarrowia lipolytica* as a Chassis for de Novo Synthesis of Five Aromatic-Derived Natural Products and Chemicals. *ACS Synth. Biol.* **9**, 2096–2106 (2020).

7. Larroude, M., Onésime, D., Rué, O., Nicaud, J. M. & Rossignol, T. A *Yarrowia lipolytica* strain engineered for pyomelanin production. *Microorganisms* **9**, (2021).

8. European Food Safety Authority (EFSA). Scientific Opinion on the re-evaluation of beetroot red (E 162) as a food additive. *EFSA Journal* vol. 13 at https://doi.org/10.2903/j.efsa.2015.4318 (2015).

9. Mordor Intelligence. Natural Food Colorants Market Analysis - Industry Report - Trends, Size & Share. https://www.mordorintelligence.com/industry-reports/global-natural-food-colorants-market (2022).

10. Polaris Market Research. Natural Food Colors Market Size Global Report, 2022 - 2030. https://www.polarismarketresearch.com/industry-analysis/natural-food-colors-market (2022).

11. Made-in-China. Natural Food Colorants Color Pigments Red Beet Red Powder - China Beet Red and Beet Red Powder. https://recedar.en.made-in-china.com/product/gZMtnVwJafpW/China-Natural-Food-Colorants-Color-Pigments-Red-Beet-Red-Powder.html (2023).

12. Made-in-China. Seeds Dried Juice Extract Fresh Jordan Bulk Hybrid Beetroot Juice Powder Supplement Beetroot Powder - China Beetroot Powder E162 Beetroot Powder and Beetroot Juice Powder Red Beetroot Powder. https://wellgreenxa.en.made-in-

china.com/product/NnaYDuCdYJcU/China-Seeds-Dried-Juice-Extract-Fresh-Jordan-Bulk-Hybrid-Beetroot-Juice-Powder-Supplement-Beetroot-Powder.html (2023).

13. Meilleur du Chef. Colorante alimentare in polvere rosso barbabietola - idrosolubile - 500 g - Selectarôme. https://www.meilleurduchef.com/it/comperare/pasticceria/ingredienti/aro-colorante-alimentare-in-polvere-rosso-barbabietola-idrosolubile-500-g.html (2023).

14. Cartrite. 500 grams Betanin Beetroot Red E162 natural water soluble food dye colour colouring powder. https://www.cartrite.co.uk/product/500-grams-betanin-beetroot-red-e162-natural-water-soluble-food-dye-colour-colouring-powder/ (2023).

15. Cartrite. 1kg Betanin Beetroot Red E162 natural water soluble food dye colour colouring powder. https://www.cartrite.co.uk/product/1kg-betanin-beetroot-red-e162-natural-water-soluble-food-dye-colour-colouring-powder/ (2023).

16. Cartrite. Betanin Beetroot Red – E162 Natural Red 33 7659-95-2 dye – 10 kilograms. https://www.cartrite.co.uk/product/betanin-beetroot-red-e162-natural-red-33-7659-95-2-dye-10-kilograms/ (2023).

17. Cartrite. Betanin Beetroot Red – E162 Natural Red 33 7659-95-2 dye – 5 kilograms. https://www.cartrite.co.uk/product/betanin-beetroot-red-e162-natural-red-33-7659-95-2-dye-5-kilograms/ (2023).

18. Wileu, R. C. & Lee, Y. Recovery of Betalaines From Red Beets By a Diffusion-Extraction Procedure. *J. Food Sci.* **43**, 1056–1058 (1978).

19. Quintero, J., Moncada, J. & Cardona, C. A. Techno-economic analysis of bioethanol production from lignocellulosic residues in Colombia: A process simulation approach. *Bioresour. Technol.* **139**, 300–307 (2013).

20. Ciriminna, R. *et al.* Betanin: A Bioeconomy Insight into a Valued Betacyanin. *ACS Sustain. Chem. Eng.* **6**, 2860–2865 (2018).

21. McCarthy, W. P. *et al.* Chlorate and Other Oxychlorine Contaminants Within the Dairy Supply Chain. *Compr. Rev. Food Sci. Food Saf.* **17**, 1561–1575 (2018).

22. Wolfram. Wolfram|Alpha: Inteligencia Computacional. https://www.wolframalpha.com/ (2023).

23. Genius, I. International Trade Databases for Import-Export Businesses | ImportGenius. https://www.importgenius.com/ (2023).

24. Panjapakkul, W. & El-Halwagi, M. M. Technoeconomic Analysis of Alternative Pathways of Isopropanol Production. *ACS Sustain. Chem. Eng.* **6**, 10260–10272 (2018).

25. Owsianiak, M., Ryberg, M. W., Renz, M., Hitzl, M. & Hauschild, M. Z. Environmental Performance of Hydrothermal Carbonization of Four Wet Biomass Waste Streams at Industry-Relevant Scales. *ACS Sustain. Chem. Eng.* **4**, 6783–6791 (2016).
