# Supplementary material for: Beet red food colourant can be produced more sustainably with engineered Yarrowia lipolytica
Source: Nat Microbiol. 2023 Nov 29;8(12):2290–303. doi: 10.1038/s41564-023-01517-5 (PMC10686825; doi:10.1038/s41564-023-01517-5)
Supplement: Supplementary file 2 — Reporting Summary [file 41564_2023_1517_MOESM2_ESM.pdf]

nature portfolio

Corresponding author(s): Irina Borodina
Sumesh Sukumara

Last updated by author(s): Sep 13, 2023

Reporting Summary

Nature Portfolio wishes to improve the reproducibility of the work that we publish. This form provides structure for consistency and transparency in reporting. For further information on Nature Portfolio policies, see our Editorial Policies and the Editorial Policy Checklist.

Statistics

For all statistical analyses, confirm that the following items are present in the figure legend, table legend, main text, or Methods section.

n/a	Confirmed	
☐	☒	The exact sample size (*n*) for each experimental group/condition, given as a discrete number and unit of measurement
☐	☒	A statement on whether measurements were taken from distinct samples or whether the same sample was measured repeatedly
☐	☒	The statistical test(s) used AND whether they are one- or two-sided<br>*Only common tests should be described solely by name; describe more complex techniques in the Methods section.*
☒	☐	A description of all covariates tested
☐	☒	A description of any assumptions or corrections, such as tests of normality and adjustment for multiple comparisons
☐	☒	A full description of the statistical parameters including central tendency (e.g. means) or other basic estimates (e.g. regression coefficient) AND variation (e.g. standard deviation) or associated estimates of uncertainty (e.g. confidence intervals)
☐	☒	For null hypothesis testing, the test statistic (e.g. *F*, *t*, *r*) with confidence intervals, effect sizes, degrees of freedom and *P* value noted<br>*Give P values as exact values whenever suitable.*
☒	☐	For Bayesian analysis, information on the choice of priors and Markov chain Monte Carlo settings
☒	☐	For hierarchical and complex designs, identification of the appropriate level for tests and full reporting of outcomes
☒	☐	Estimates of effect sizes (e.g. Cohen's *d*, Pearson's *r*), indicating how they were calculated

Our web collection on statistics for biologists contains articles on many of the points above.

Software and code

Policy information about availability of computer code

Data collection	The Chromeleon 7 software (Thermo Fisher Scientific, US) was used to collect the HPLC data. UvVis absorbance and fluorescence data was acquired by plate reader (BioTek Elx 8089, US). Trading data was acquired from Import Genius, the EcoInvent database 3.8 was used for collecting the emissions inventory, and Textbook rules of thumbs were used for collecting equipment costs.
Data analysis	The Chromeleon 7 software (Thermo Fisher Scientific, US) was used to analyze the HPLC data and generate standard curves. The fermentation process was modeled using the SuperPro Designer v11 software. The Life Cycle Assessment (LCA) was performed using SimaPro v9.3.0.3. Microsoft applications (Excel and VBA) were used for analysis of economic and environmental assessment results. Open-source python libraries (Plotly (5.16.1), Seaborn (0.11.2), Matplotlib (3.5.1), Pandas (1.4.2), and Numpy (1.21.5)) were used for data analysis and plotting.

For manuscripts utilizing custom algorithms or software that are central to the research but not yet described in published literature, software must be made available to editors and reviewers. We strongly encourage code deposition in a community repository (e.g. GitHub). See the Nature Portfolio guidelines for submitting code & software for further information.

Data

Policy information about availability of data

All manuscripts must include a data availability statement. This statement should provide the following information, where applicable:

- Accession codes, unique identifiers, or web links for publicly available datasets
- A description of any restrictions on data availability
- For clinical datasets or third party data, please ensure that the statement adheres to our policy

Data used, generated, or analyzed is available in the supplementary files. The source data used to generate the graphs for this study can be found in the 'Source data' files. The nucleotide and amino acid sequences for all heterologous and native Y. lipolytica genes used for engineering can be found in Supplementary file 1. A list of all biobricks, plasmids, strains, and oligonucleotides (primers) used and/or generated in this work can be found in Supplementary data 1. The medium composition for the fermentations, as well as the operational parameters can be found in Supplementary data 2 – along with the raw online data. The Emissions inventory collected from the EcoInvent database 3.8 used for the LCA can be found in Supplementary data 3. All other data collected and used for the LCA and TEA can be found in Supplementary file 1.

Research involving human participants, their data, or biological material

Policy information about studies with human participants or human data. See also policy information about sex, gender (identity/presentation), and sexual orientation and race, ethnicity and racism.

Reporting on sex and gender	N/A
Reporting on race, ethnicity, or other socially relevant groupings	N/A
Population characteristics	N/A
Recruitment	N/A
Ethics oversight	N/A

Note that full information on the approval of the study protocol must also be provided in the manuscript.

Field-specific reporting

Please select the one below that is the best fit for your research. If you are not sure, read the appropriate sections before making your selection.

☒ Life sciences ☐ Behavioural & social sciences ☐ Ecological, evolutionary & environmental sciences

For a reference copy of the document with all sections, see nature.com/documents/nr-reporting-summary-flat.pdf

Life sciences study design

All studies must disclose on these points even when the disclosure is negative.

Sample size	Small-scale cultivations were carried out in triplicate, with the exception of the L-tyrosine supplementation cultivation, the shakeflask buffer test cultivations, and the betanin deglycosylation assay which was carried out in duplicate. Fed-batch fermentations in bioreactor were carried out in duplicate. No sample-size calculation was performed, rather sample-size was typically set at three (3) for primary experiments to achieve acceptable statistical power in a cost- and time-efficient manner.
Data exclusions	Throughout the design-build-test metabolic engineering cycle, strains were tested against each other iteratively to determine the impact of the genetic modifications or cultivation conditions on betalain production. Data from all cycles were not included in the manuscript, rather all strains were cultivated in biological triplicates anew at the end of the strain engineering campaign at identical conditions to ensure a fair comparison. No other data were excluded from analysis.
Replication	The strains performed identically relative to each other throughout the metabolic engineering cycles (5), where they were typically tested against each other in biological duplicate or triplicate to identify beneficial modifications.
Randomization	Strain position in deep-well plates can somewhat impact growth due to slight variations in oxygen availability/transfer, however the well positions were not randomized as this significantly confuses the investigators ability to carry out the experiment correctly. On the other hand, strain position in deep-well plates were also not fixed and changed randomly for each strain engineering / cultivation round.
Blinding	Investigators were not entirely blinded during data collection and analysis, however only internal non-informative strain numbers were used for the strain engineering, cultivation, and analysis.

Reporting for specific materials, systems and methods

We require information from authors about some types of materials, experimental systems and methods used in many studies. Here, indicate whether each material, system or method listed is relevant to your study. If you are not sure if a list item applies to your research, read the appropriate section before selecting a response.

Materials & experimental systems

n/a	Involved in the study
☒	☐ Antibodies
☐	☒ Eukaryotic cell lines
☒	☐ Palaeontology and archaeology
☒	☐ Animals and other organisms
☒	☐ Clinical data
☒	☐ Dual use research of concern
☒	☐ Plants

Methods

n/a	Involved in the study
☒	☐ ChIP-seq
☒	☐ Flow cytometry
☒	☐ MRI-based neuroimaging

Eukaryotic cell lines

Policy information about cell lines and Sex and Gender in Research

Cell line source(s)	While eukaryotic cell lines were used in this study, they were of the type yeast Yarrowia lipolytica. All Yarrowia lipolytica strains generated in this study were derived from W29/CLIB89 (NRRL Y-63746).
Authentication	None of the cell lines used were authenticated as this is not relevant for Yarrowia lipolytica.
Mycoplasma contamination	None of the cell lines were tested for mycoplasma contamination as this is typically not an issue in yeast cultivation.
Commonly misidentified lines (See ICLAC register)	No commonly misidentified cell lines were used in this study.
